# Two Diterpene Synthases from *Chryseobacterium*: Chryseodiene Synthase and Wanjudiene Synthase

**DOI:** 10.1002/anie.202004691

**Published:** 2020-05-18

**Authors:** Lukas Lauterbach, Bernd Goldfuss, Jeroen S. Dickschat

**Affiliations:** ^1^ Kekulé-Institute for Organic Chemistry and Biochemistry University of Bonn Gerhard-Domagk-Straße 1 53121 Bonn Germany; ^2^ Institute for Organic Chemistry University of Cologne Greinstraße 4 50939 Cologne Germany

**Keywords:** biosynthesis, enzyme mechanisms, isotopes, NMR spectroscopy, terpenes

## Abstract

Two bacterial diterpene synthases (DTSs) from *Chryseobacterium* were characterised. The first enzyme yielded the new compound chryseodiene that closely resembles the known fusicoccane diterpenes from fungi, but its experimentally and computationally studied cyclisation mechanism is fundamentally different to the mechanism of fusicoccadiene synthase. The second enzyme produced wanjudiene, a diterpene hydrocarbon with a new skeleton, besides traces of the enantiomer of bonnadiene that was recently discovered from *Allokutzneria albata*.

## Graphical abstract

**Two diterpene synthases** from the bacterial genus *Chryseobacterium* making the new compounds chryseodiene and wanjudiene are reported. The enzyme mechanisms were investigated in detail through a combination of experimental and computational approaches.

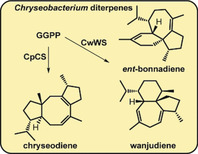

Terpene synthases (TSs) are remarkable enzymes that convert the oligoprenyl diphosphates geranyl (GPP), farnesyl (FPP), geranylgeranyl (GGPP) and geranylfarnesyl diphosphate (GFPP) into complex, usually polycyclic terpenes with multiple stereocentres.^1^ For type I TSs, these astonishing reactions are initiated by diphosphate abstraction from the substrate. Depending on the conformation of the resulting cation provided by the enzyme's active site architecture, a cascade reaction involving ring closures by the attack of π‐bonds to cationic centres, hydride shifts and Wagner–Meerwein rearrangements, proton transfers, and a deprotonation or attack of a nucleophile for termination leads to the terpene product.^2^ These multi‐step reactions and its intermediates cannot be observed directly, but the complex reaction mechanisms can be studied experimentally using isotopically labelled substrates,^3^ or theoretically by quantum chemical calculations.^4^ Several bacterial mono‐, sesqui‐ and diterpene synthases have been reported,^1^, ^5^, ^6^ mostly from actinobacteria and in particular from the genus *Streptomyces*. A few examples from cyanobacteria,^7^ chloroflexi,^6^, ^8^ deltaproteobacteria^6^, ^9^ and chitinophagia are also known.^8^, ^10^ A phylogenetic analysis of bacterial TS homologs extracted from the available genome sequences by BLAST search (Figure S1 in the Supporting Information) demonstrated the presence of TSs in various so far untapped phyla. In particular, many presumptive TSs were observed in the genus *Chryseobacterium* (flavobacteria). Here we report on two new diterpene synthases (DTSs) from *C. polytrichastri* DSM 26899 and *C. wanjuense* DSM 17724, structure elucidation of their products and investigations on the enzyme mechanisms by labelling experiments and computational chemistry.

Two TS genes were cloned into the expression vector pYE‐Express^11^ giving access to the recombinant purified proteins (Figure S2). Both enzymes exhibited the highly conserved motifs of type I TSs, including the aspartate‐rich motif, the NSE triad, the pyrophosphate sensor and the RY pair (Figure S3). The first enzyme from *C. polytrichastri* (WP_073290622) did not yield a product from GPP, FPP and GFPP, but efficiently converted GGPP into a diterpene (Figure S4). The compound was isolated for structure elucidation by NMR spectroscopy (Table S2, Figures S5–S11), resulting in the new compound chryseodiene (**1**, Scheme 1). Thus, the DTS is a *Chryseobacterium polytrichastri* chryseodiene synthase (CpCS).

**Scheme 1:**
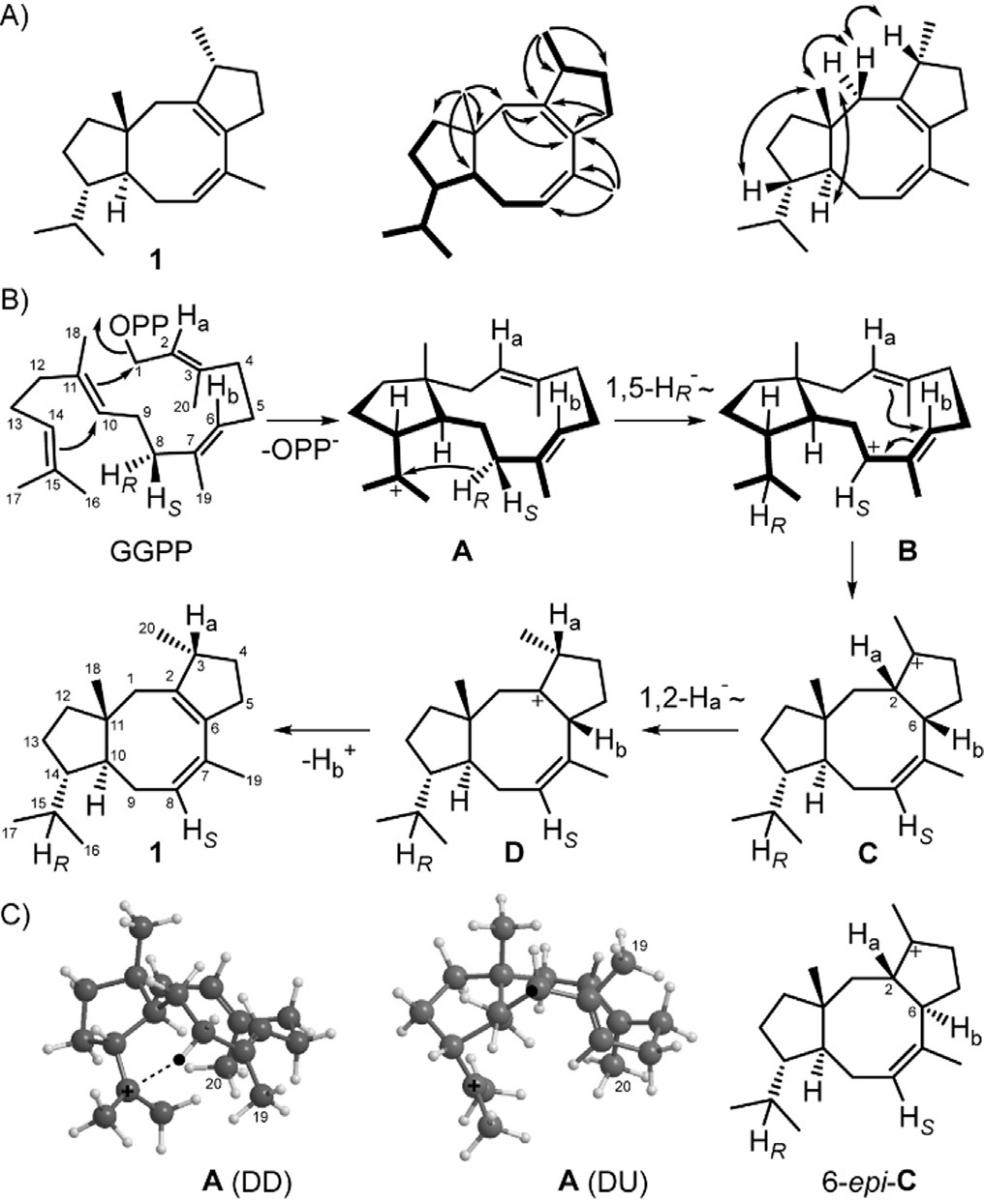
A) Structure of **1**. Bold lines: ^1^H,^1^H‐COSY, single‐headed arrows: HMBC, double‐headed arrows: NOESY correlations. Carbon numbering indicates the origin of each carbon from GGPP by same number. B) Cyclisation mechanism from GGPP to **1** by CpCS. C) Conformers of **A** with Me19 and Me20 pointing down (DD) and with Me20 down and Me19 up (DU). The migrating H_*R*_ at C8 is shown by black dot. Structure of the hypothetical intermediate 6‐*epi*‐**C**.

The absolute configuration of **1** was determined using stereoselectively deuteriated terpene precursors. Incubation of (*E*)‐ and (*Z*)‐(4‐^13^C,4‐^2^H)isopentenyl diphosphate^12^ (IPP) and dimethylallyl diphosphate (DMAPP) with GGPP synthase (GGPPS) from *Streptomyces cyaneofuscatus* 13 and CpCS resulted in the formation of labelled **1** with stereoselective deuteriation at C4 and C12 (Figure S12). The additional ^13^C‐label at these carbons allowed for a sensitive analysis by HSQC spectroscopy. The introduced stereochemical anchors of known configuration gave access to the absolute configuration of **1** as shown in Scheme 1, based on the full relative assignment of all hydrogen atoms from its NOESY spectrum (Figure S11). The absolute configuration of **1** was confirmed by similar experiments with (*R*)‐ and (*S*)‐(1‐^13^C,1‐^2^H)IPP,^14^ IPP isomerase (IDI) from *Serratia plymuthica*,^15^ GGPPS and CpCS for stereoselective deuteriation at C1, C5, C9 and C13 (Figure S13).

The proposed cyclisation mechanism for **1** starts from GGPP by a 1,11‐10,14‐cyclisation to cation **A**, followed by a 1,5‐hydride shift to **B** and a third cyclisation to **C**. A subsequent 1,2‐hydride migration to **D** and deprotonation yields **1**. This mechanism was experimentally supported by twenty ^13^C‐labelling experiments with introduction of labelling into each carbon of **1**, using the corresponding synthetic (^13^C)GGPP isotopomers (Figure S14).^13^ The 1,5‐hydride shift from **A** to **B** was evident from the incubation experiments with DMAPP and (*E*)‐ and (*Z*)‐(4‐^13^C,4‐^2^H)IPP, resulting in a stereoselective deuterium labelling at C8. The hydride migration from C8 into the *i*Pr group of **1** can be followed by its cleavage during EI‐MS fragmentation, demonstrating the specific migration of the 8‐*pro*‐*R* hydrogen introduced from (*Z*)‐(4‐^13^C,4‐^2^H)IPP (Figure S15), while the 8‐*pro*‐*S* hydrogen from (*E*)‐(4‐^13^C,4‐^2^H)IPP remains bound to C8, as shown by a vanished crosspeak in the HSQC spectrum (Figure S16). The 1,2‐hydride transfer from **C** to **D** (H_a_) was investigated by incubation of (3‐^13^C,2‐^2^H)GGPP^13^ with CpCS, resulting in a triplet for C3 in the ^13^C‐NMR spectrum that indicated a direct ^13^C−^2^H bond for the obtained product (Figure S17). The final deprotonation proceeds with loss of the hydrogen from C6 of GGPP (H_b_), as followed by incubation of (2‐^2^H)FPP and IPP with GGPPS and CpCS and product analysis by GC/MS (Figure S18).

While the configuration at C2 of **C** can be inferred from the structure of **1**, because the subsequent 1,2‐hydride migration to **D** must proceed suprafacially, the configuration at C6 is based on DFT calculations for the cyclisation to **1**. Two conformers of **A** can explain the correct stereochemistry at C2 of **C** (Scheme 1 C), but their computed structures demonstrate that only the DD conformer (Me19 and Me20 pointing down) allows for the 1,5‐hydride shift to **B**, while this reaction is not possible for the DU conformer (Me20 down, Me19 up) that is the required precursor of the hypothetical intermediate 6‐*epi*‐**C**. The DFT calculations further reveal a low transition state (TS) barrier for the 1,5‐hydride shift from **A** (DD) to **B** (Figure 1). After a conformational rearrangement of **B**, the subsequent cyclisation to **C** also proceeds smoothly, followed by a 1,2‐hydride transfer to **D** through the highest TS barrier of the profile with 6.69 kcal mol^−1^. The sequence from **A** to **D** is exergonic with −11.11 kcal mol^−1^.

**Figure 1:**
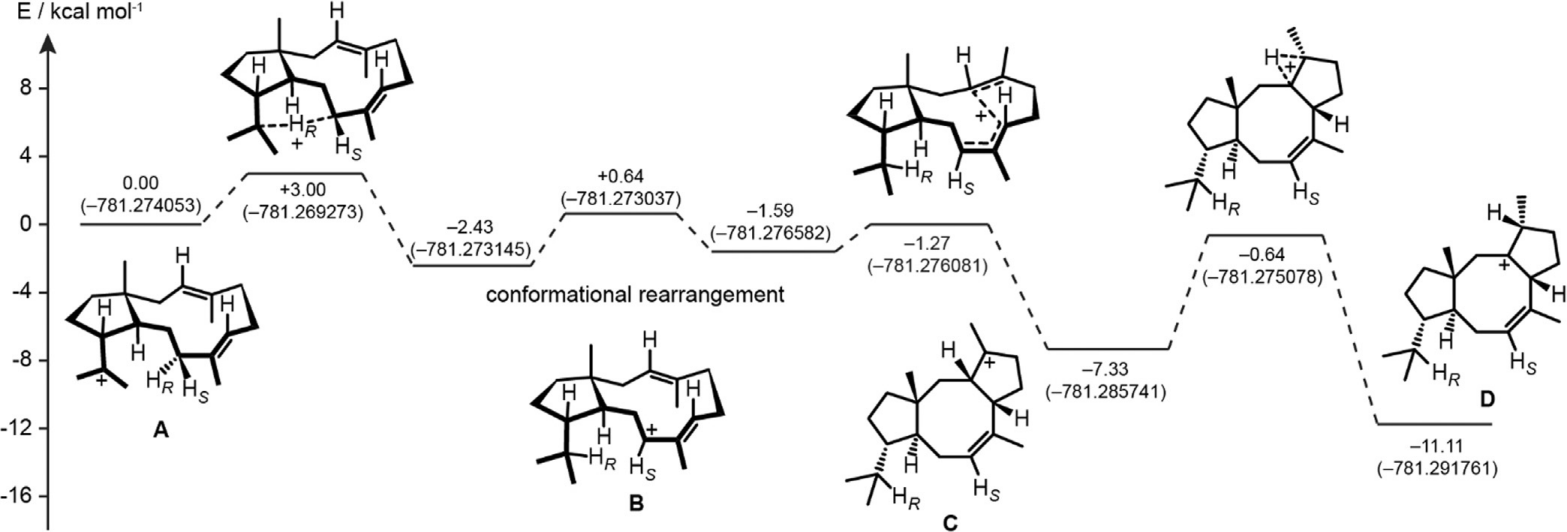
Computational B97D3/6‐31G** analysis of the cationic cascade reaction from **A** to **D** in the cyclisation of GGPP to **1** catalysed by CpCS. Relative energies are given in kcal mol^−1^ (total energies in Hartree).

Notably, **1** is structurally related to fusicoccane diterpenes (Scheme 2 A), including fusicocca‐2,10(14)‐diene (**2**)^16^ that is made by the fusicoccadiene synthases from *Phomopsis amygdali* (PaFS) and *Alternaria brassicicola* (AbFS),^17^ cyclooctat‐9‐en‐7‐ol (**3**) formed by CotB2 in *Streptomyces melanosporofaciens*,^18^ cycloaraneosene (**4**) made by the DTS SdnA in *Sordaria araneosa*,^19^ and the recently reported myrothec‐15(17)‐en‐7‐ol (**5**) produced by the DTS MgMS in *Myrothecium graminearum*.^20^ Also the sesterterpene ophiobolin F (**6**), the product of AcOS in *Aspergillus clavatus*,^21^ exhibits a highly similar core structure, but the cyclisation mechanisms of all these enzymes are different to the mechanism of CpCS, as a different stereochemistry is installed. To give an example, isotopic labelling experiments in conjunction with a computational study^22^ support a mechanism for PaFS with 1,11‐10,14‐cyclisation of GGPP to **E** which may be identical to **A** or resemble its C14 epimer, in both cases with the same configuration at C11 (compare Schemes 1 B and 2B). Because of a subsequent 1,2‐hydride shift of H_d_ and a 1,4‐proton transfer of H_c_ from C10 to C2 to form **F** the stereocentre at C14 of **E** cannot be inferred from the structure of the product **2**. H_c_ is then passed on by a 1,5‐proton transfer to C6 in **G**, followed by a 1,2‐hydride migration of H_b_ and ring closure to **H**. This step proceeds with *cis* fusion of the newly formed rings, but with inverted stereochemistry at C2 and C6 compared to intermediate **C** in the cyclisation to **1**, to explain the structure of the side product fusicocca‐3(16),10(14)‐diene (**7**)^23^ by deprotonation from Me20, while loss of H_a_ yields the main product **2**. The mechanistic and stereochemical differences are also reflected by the weak identity (15 %) between CpCS and the TS domain of PaFS, suggesting that these enzymes have evolved independently. Unrelated DTSs are also known for phomopsene from fungi and bacteria,^24^ while bacterial and fungal corvol ether synthases are closely related, suggesting a cross‐kingdom horizontal gene transfer.^25^

**Scheme 2:**
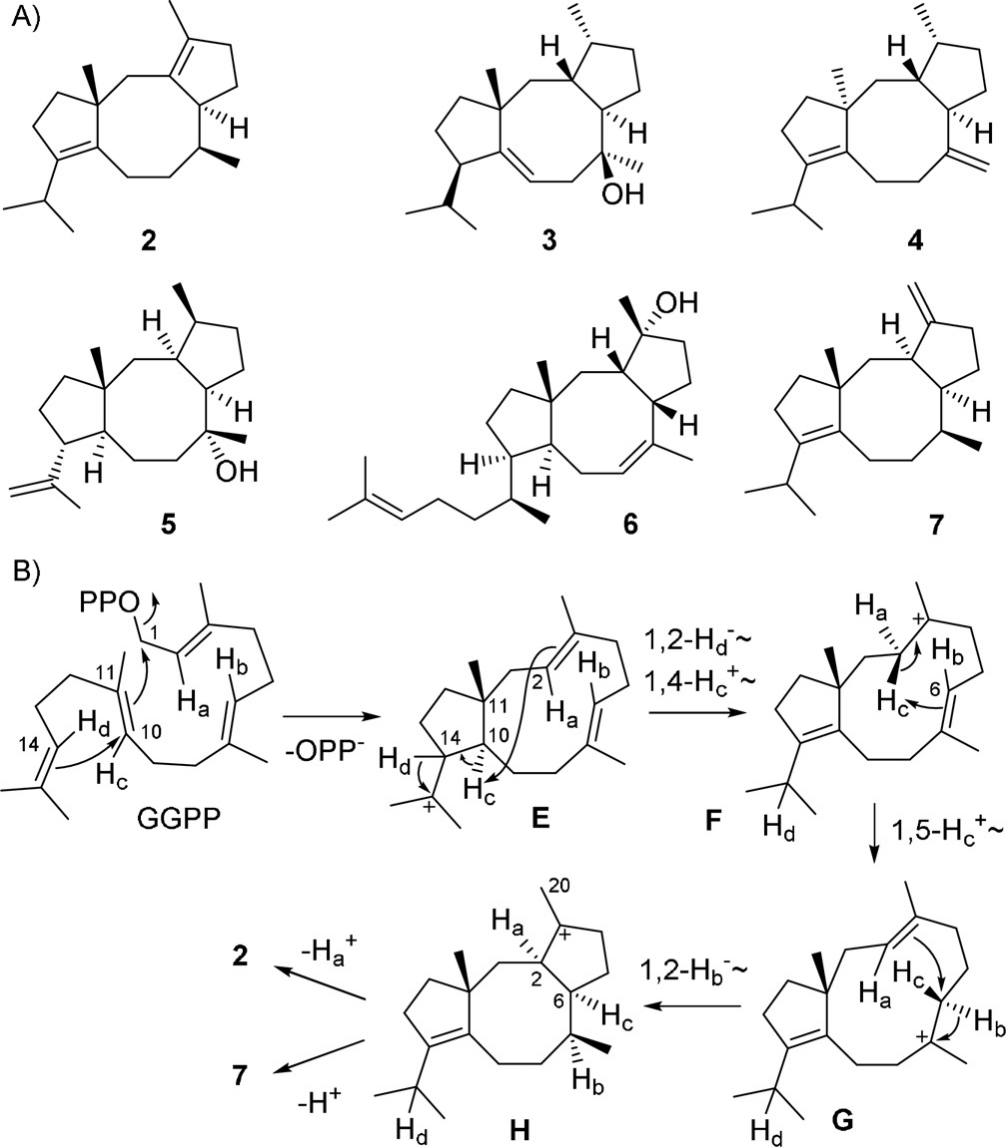
Cyclisation mechanism from GGPP to **2** and **3** by PaFS.

The second enzyme from *C. wanjuense* (WP_089795910) did not accept GPP, FPP or GFPP, but efficiently converted GGPP into an unkown diterpene **8**, besides traces of bonnadiene (**9**) for which the bonnadiene synthase (BdS) was recently discovered from *Allokutzneria albata* (Scheme 3, Figure S19).^12^ Product isolation and structure elucidation by NMR spectroscopy (Table S4, Figures S20–S26) resulted in the structure of the new compound wanjudiene (**8**) and characterised the DTS as *Chryseobacterium wanjuense* wanjudiene synthase (CwWS). The absolute configuration of **8** was established through stereoselective deuteriation by conversion of DMAPP and (*E*)‐ and (*Z*)‐(4‐^13^C,4‐^2^H)IPP (Figure S27), and (*R*)‐ and (*S*)‐(1‐^13^C,1‐^2^H)IPP (Figure S28) with GGPPS and CwWS. All experiments pointed to the absolute configuration for **8** as shown in Scheme 3.

**Scheme 3:**
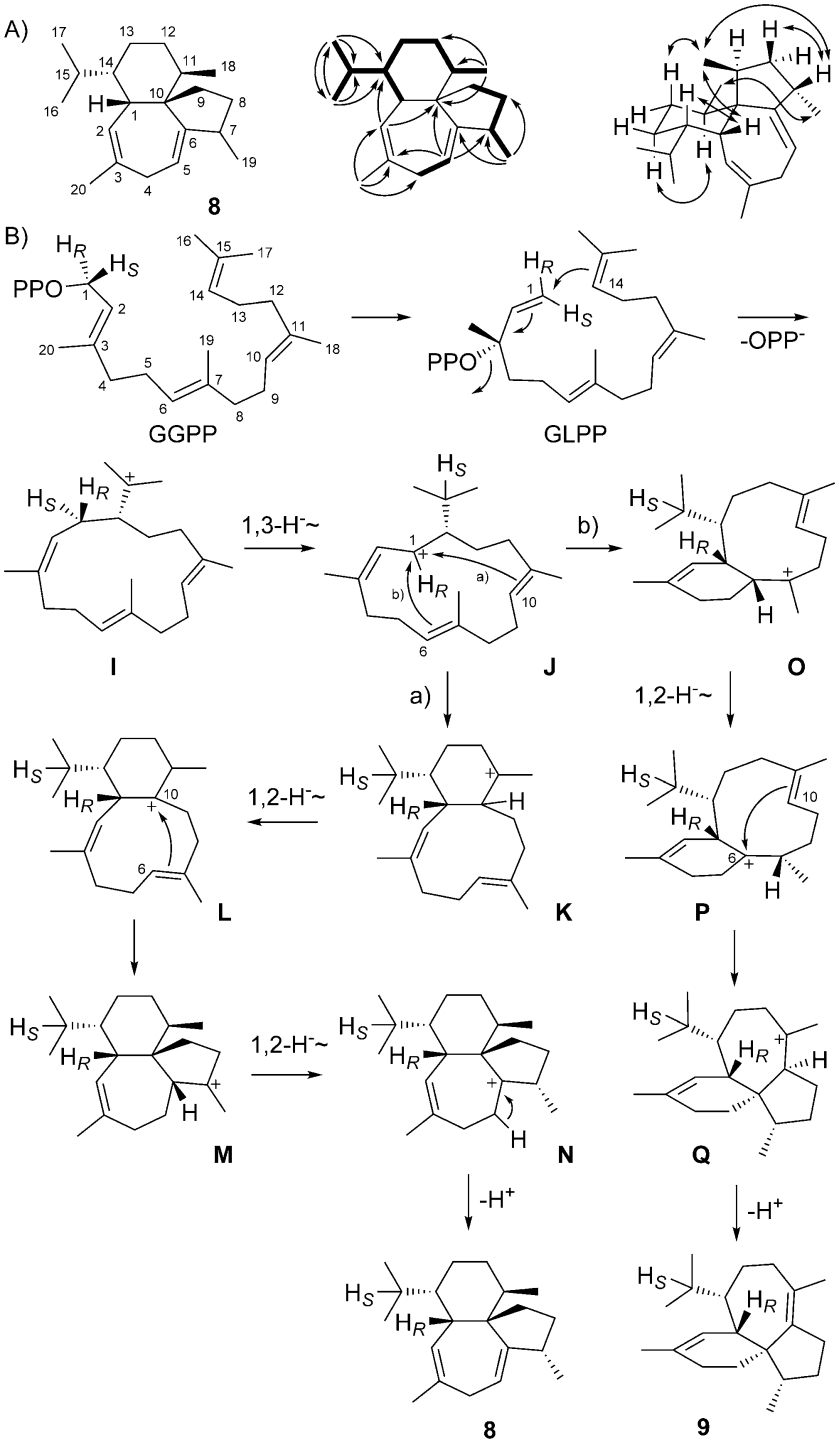
A) Structure of **8**. Bold lines: ^1^H,^1^H‐COSY, single‐headed arrows: HMBC, double‐headed arrows: NOESY correlations. Carbon numbering indicates the origin of each carbon from GGPP by same number. B) Cyclisation mechanism from GGPP to the main product **8** and the side product **9** by CwWS.

The proposed cyclisation mechanism from GGPP to **8** starts with the isomerisation of GGPP to GLPP, followed by 1,14‐cyclisation to **I**. The isomerisation to GLPP is required to explain the *Z* configured double bond in **I**. A subsequent 1,3‐hydride shift to **J** is followed by 1,10‐cyclisation to **K** (path a). The downstream steps include a 1,2‐hydride migration to **L**, 6,10‐cyclisation to **M**, another 1,2‐hydride shift to **N** and final deprotonation. Alternatively, cation **J** can react by 1,6‐cyclisation to **O** (path B), followed by 1,2‐hydride shift to **P**, 6,10‐cyclisation to **Q** and deprotonation to the side product **9**. The cyclisation mechanism to **8** was investigated by enzymatic conversion of all twenty isotopomers of (^13^C)GGPP with CwWS, resulting in the incorporation of labelling into the expected positions in all cases (Figure S29). The 1,3‐hydride shift from **I** to **J** was followed using (11‐^13^C)FPP with (*R*)‐ and (*S*)‐(1‐^2^H)IPP, GGPPS and CwWS, resulting in a triplet for C15 in the ^13^C‐NMR due to ^13^C–^2^H spin coupling for the product from (*S*)‐(1‐^2^H)IPP, while a singlet was observed with the *R* enantiomer, indicating specific migration of the 1‐*pro*‐*S* hydrogen of GGPP (Figure S30). Also for the side product **9** the 1‐*pro*‐*S* hydrogen of GGPP shifts into the *i*Pr group, which is evident from its EIMS fragmentation with loss of deuterium for labelled **9** from (*S*)‐(1‐^2^H)IPP, while EIMS cleavage of the *i*Pr group for labelled **9** from (*R*)‐(1‐^2^H)IPP proceeds with retainment of deuterium (Figure S31). Notably, for **9** obtained with BdS from *Allokutzneria albata* the opposite outcome with a shift of the 1‐*pro*‐*R* hydrogen of GGPP into the *i*Pr group was observed,^12^ suggesting that **9** from BdS and the side product from CwWS are enantiomers (Figure S32).^26^

The second hydride migration in the biosynthesis of **8** from **K** to **L** was evident from the incubation of (3‐^13^C,2‐^2^H)GPP and IPP with GGPPS and CwWS, yielding labelled **8** with a slightly upfield shifted triplet for C11 (Figure S33). Similarly, the enzymatic conversion of (3‐^13^C,2‐^2^H)FPP and IPP with GGPPS and CwWS gave a strongly enhanced and upfield shifted triplet for C7, giving experimental support for the 1,2‐hydride transfer from **M** to **N** (Figure S34). Finally, the stereospecificity of the deprotonation from **N** to **8** was elucidated from the incubation of CwWS with (*R*)‐ and (*S*)‐(1‐^13^C,1‐^2^H)IPP, showing an enhanced HSQC signal for C5 from the *R* enantiomer, while no signal is observed when using (*S*)‐(1‐^13^C,1‐^2^H)IPP (Figure S35).

The production of diterpenes by *C. polytrichastri* and *C. wanjuense* was investigated by collecting the volatiles on charcoal filter traps using a closed‐loop stripping apparatus (CLSA),^27^ followed by extraction of the filters and GC/MS analysis of the extracts. *C. polytrichastri* did not produce **1** or any other diterpene (Figure S36), suggesting that the *CpCS* gene is not expressed under laboratory growth conditions, while *C. wanjuense* emitted **8** as one of the major volatiles (Figure S37).

Recently a linalool synthase producing mainly (*R*)‐linalool with ca. 60 % *ee* was reported from *C. polytrichastri*,^28^ but this study describes the first DTSs with more complex enzyme functions from this genus. The first enzyme (CpCS) produced the fusicoccane‐type diterpene chryseodiene (**1**) through a mechanism that was elucidated by a combination of experimental and computational work and that is distinct from the mechanism of other fusicoccane‐type DTSs. The second DTS (CwWS) catalyses the formation of wanjudiene (**8**), a diterpene with a previously unknown skeleton, and also provides the enantiomer **9** of the known compound bonnadiene as a side product. This surprising result coincides with a large phylogenetic distance between CwWS and BdS (Figure S1), but also suggests that some terpene structures may be privileged and may be more easily accessible by enzyme reactions than others. Future studies will presumably show more of such surprises, emphasising the importance of continuous thorough investigations on the intriguing and complex mechanisms of terpene synthases.

## Conflict of interest

The authors declare no conflict of interest.

## Supporting information

As a service to our authors and readers, this journal provides supporting information supplied by the authors. Such materials are peer reviewed and may be re‐organized for online delivery, but are not copy‐edited or typeset. Technical support issues arising from supporting information (other than missing files) should be addressed to the authors.

Supplementary
