# Supplementary material for: Two Diterpene Synthases from Chryseobacterium: Chryseodiene Synthase and Wanjudiene Synthase
Source: Angew Chem Int Ed Engl. 2020 May 18;59(29):11943–7. doi: 10.1002/anie.202004691 (PMC7383580; doi:10.1002/anie.202004691)
Supplement: Supplementary file 1 — Supplementary [file ANIE-59-11943-s001.pdf]

Angewandte Chemie
Eine Zeitschrift der Gesellschaft Deutscher Chemiker

Supporting Information

Two Diterpene Synthases from *Chryseobacterium*: Chryseodiene Synthase and Wanjudiene Synthase

*Lukas Lauterbach, Bernd Goldfuss, and Jeroen S. Dickschat**

anie_202004691_sm_miscellaneous_information.pdf

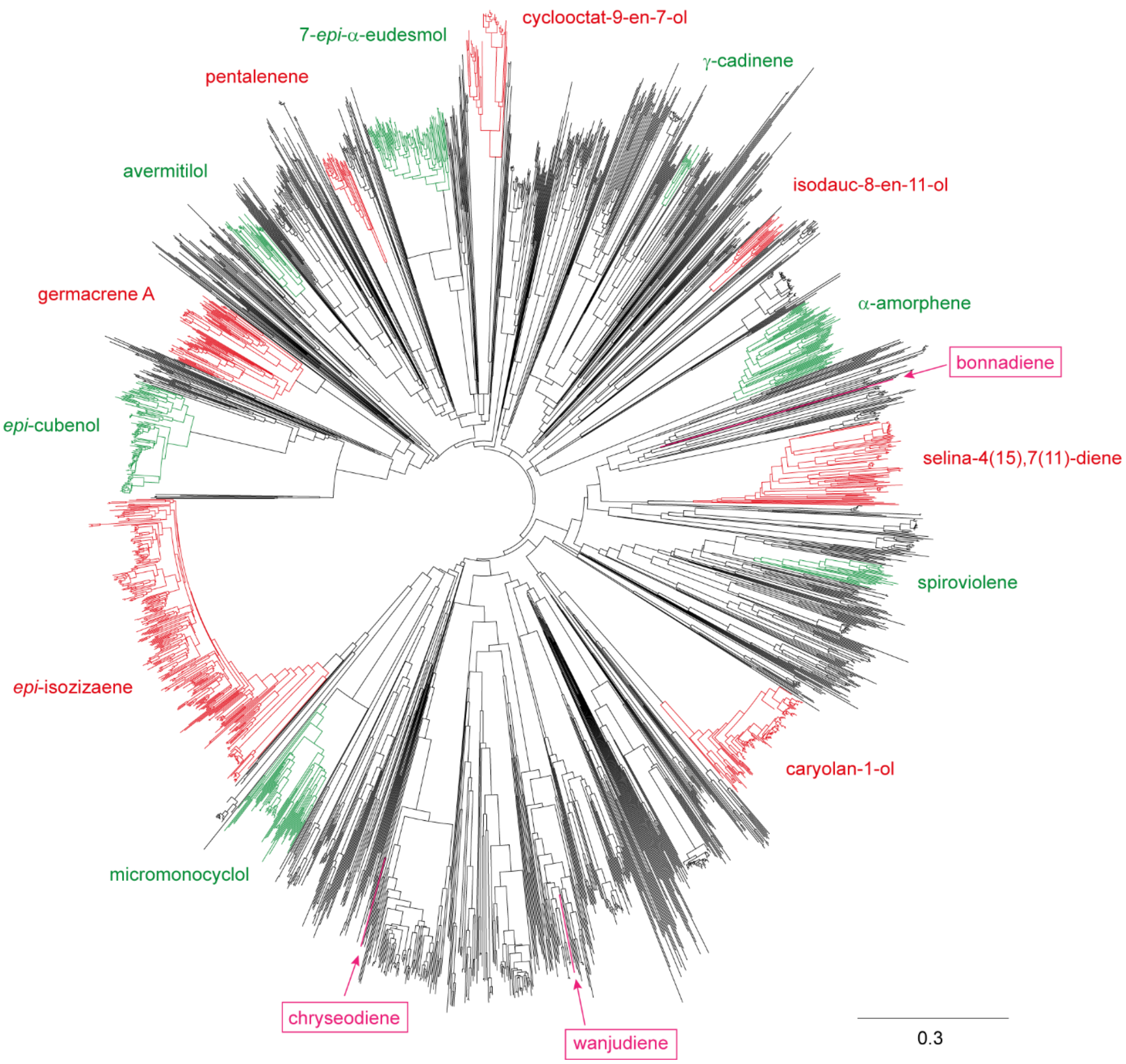

Figure S1. Phylogenetic tree of bacterial TS homologs. Enzymes discussed in this study are shown in boxes, characterised enzymes and their closest homolgs with likely the same function are shown in red and green.

Strains and culture conditions
Chryseobacterium polytrichastri DSM 26899 and *Chryseobacterium wanjuense* DSM17724 were obtained from DSMZ (Braunschweig, Germany) and were cultivated on medium 1496 (5.0 g tryptone, 3.0 g yeast extract, 1.0 g glucose, 1.0 L H_2O, pH 7.0, for agar plates 15.0 g agar-agar was added) at 28 °C. *Saccharomyces cerevisiae* FY834 was grown in liquid YPAD medium (10.0 g yeast extract, 20.0 g peptone, 20.0 g glucose, 400 mg adenine sulphate, 1.0 L H_2O) or on SM-URA agar plates (1.7 g yeast nitrogen base, 5.0 g ammonium sulphate, 20.0 g glucose, 770 mg nutritional supplement minus uracil, 20.0 g agar-agar, 1.0 L H_2O) at 28 °C. *Escherichia coli* BL21(DE3) was grown in LB-broth (10.0 g tryptone, 5.0 g yeast extract, 5.0 g NaCl, 1.0 L H_2O, for agar plates 16 g agar-agar was added, kanamycin was used at a concentration of 50 µg mL^{-1}) at 37 °C. All media were autoclaved at 121 °C for 20 min prior to use.

GC/MS and GC/MS-QToF analyses
GC/MS analyses were performed on a 7890B GC – 5977A mass detector system (Agilent, Santa Clara, CA, USA). The GC was equipped with a HP5-MS fused silica capillary column (30 m, 0.25 mm i. d., 0.50 µm film). GC parameters were 1) inlet pressure: 77.1 kPa, He at 23.3 mL min^{-1}, 2) injection volume: 2 µL, 3) temperature program: 5 min at 50 °C increasing at 5 °C min^{-1} to 320 °C, 4) 60 s valve time, and 5) carrier gas: He at 1.2 mL min^{-1}. MS parameters were 1) source: 230 °C, 2) transfer line: 250 °C, 3) quadrupole: 150 °C and 4) electron energy: 70 eV. Retention indices (*I*) were determined in comparison to a homologous series of *n*-alkanes (C_7-C_{40}).
GC/MS-QTOF analyses were conducted on a 7890B GC – 7200 accurate-mass Q-TOF detector system (Agilent). The GC was equipped with a HP5-MS fused silica capillary column (30 m, 0.25 mm i. d., 0.50 µm film). GC parameters were 1) injection volume: 1 µL, 2) split ratio: 10:1, 60 s valve time, 3) carrier gas: He at 1 mL min^{-1}, and 4) temperature program: 5 min at 50 °C increasing at 10 °C min^{-1} to 320 °C. MS parameters were 1) inlet pressure: 83.2 kPa, He at 24.6 mL min^{-1}, 2) transfer line: 250 °C, 3) electron energy 70 eV.

NMR spectroscopy
NMR measurements were performed on a Bruker (Billerica, MA, USA) Avance III HD Prodigy (500 MHz) or on an Avance III HD Cryo (700 MHz) NMR spectrometer. Spectra were evaluated using TopSpin 3.6.2 (Bruker) and were referenced against solvent signals (^{1}H-NMR, residual proton signal: C_6D_6 δ = 7.16 ppm, ^{13}C-NMR: C_6D_6 δ = 128.06).[1]

Isolation of genomic DNA[2]
For extraction of genomic DNA a 100 mL culture of the bacteria was incubated for 3 days, harvested by centrifugation (14.610 x g, 5 min, 4 °C) and the supernatant medium was carefully decanted. To the pellet was added SET buffer (5 mL, 75 mM NaCl, 25 mM EDTA, 20 mM TRIS HCl, pH 8.0) and freshly prepared lysozyme solution (100 µL, 50 mg mL^{-1}) and the mixture was incubated at 37 °C for 30 min. SDS solution (600 µL, 10 %) and freshly prepared proteinase K solution (100 µL, 50 mg mL^{-1}) were added and the mixture was shaken for 1 h at 55 °C. The mixture was washed using a premixed phenol/chloroform/isoamyl alcohol solution (5 mL, Carl Roth, Karlsruhe, Germany), and centrifuged at 14.610 x g for 30 min to separate the layers. The aqueous layer was collected by careful pipetting and the DNA was precipitated by adding ethanol to a final percentage of 60 %. The DNA was spun down, the precipitate was washed three times with 70 % ethanol. Finally the DNA was dried overnight and redissolved in nuclease-free water.

Table S1. Primers used in this study.

Primer	Sequence[a]
LL101f	ATGATTATTTTTAAAGATACTAAATCAATAATAAC
LL101r	TTATTCTATTTTGGGTAATTCTTCTATTTG
LL102f	GGCAGCCATATGGCTAGCATGACTGGTGGAATGATTATTTTTAAAGATACTAAATCAATAATAAC
LL102r	TCTCAGTGGTGGTGGTGGTGGTGCTCGAGTTTATTCTATTTTGGGTAATTCTTCTATTTG
LL138f	ATGAAAACCATGACAAACGAAG
LL138r	TTATTTTTCATTTGTATATTCACCTTCTAC
LL139f	GGCAGCCATATGGCTAGCATGACTGGTGGAATGAAAACCATGACAAACGAAG
LL139r	TCTCAGTGGTGGTGGTGGTGGTGCTCGAGTTTATTTTTCATTTGTATATTCACCTTCTAC

[a] Homology arms for recombination in yeast fitting to the end sequences of linearised pYE-Express (HindIII and EcoRI digestion) are underlined.

Gene cloning

Genes encoding the desired terpene synthases were obtained via polymerase-chain-reaction (PCR) using Q5®-High-Fidelity DNA polymerase (New England Biolabs, Ipswich, MA, USA) together with the previously isolated genomic DNA and the primers LL101f and LL101r for WP_073290622 (CpCS) and LL138f and 138r for WP_089795910 (CwWS), respectively (Table S1). PCR was performed using a 3-step touch-up protocol with initial denaturation at 98 °C for 5 min, a first 3-step-cycle with denaturation at 98 °C for 15 s, annealing at 53 °C for 30 s, and elongation at 72 °C for 1 min (16 repetitions, annealing temperature increasing 0.5 °C per cycle), followed by a second cycle differing only in the annealing temperature being constantly at 61 °C (24 cycles) and a final elongation step at 72 °C for 5 min. Successful amplification was checked by gel electrophoresis, PCR reactions were purified using Wizard® SV Gel and PCR Clean-Up System (Promega, Madison, WI, USA). The obtained products were used for another PCR under the same conditions using LL102f and LL102r for CpCS and LL139f and LL139r for CwWS, respectively, to add homology arms for recombination in yeast. Homologous recombination was performed as previously reported for our pYE-Express shuttle vector, using the standard PEG/LiOAc/salmon sperm protocol.[3,4] Prepared *S. cerevisiae* cultures were grown on SM-URA plates for 3 days and colonies were collected to isolate a plasmid mixture with the Zymoprep™ Yeast Plasmid Miniprep II kit (Zymo Research, Irvine, CA, USA). The isolated plasmid mixture was used for electroporation using *E. coli* BL21(DE3) electrocompetent cells, which were grown overnight on LB agar plates containing kanamycin. Single colonies were picked and used to inoculate 6 mL LB with kanamycin. After 24 h incubation time plasmids were extracted using the PureYield™ Plasmid Miniprep System (Promega) and checked by analytical digest and finally by sequencing. Thus transformants carrying the plasmids pYE_CpCS and pYE_CwWS were obtained which were used for further experiments.

Gene expression and Protein purification

For gene expression a glycerol stock of the previously obtained *E. coli* transformants was used to inoculate liquid LB medium containing kanamycin. The fresh overnight culture was used to inoculate larger culture volumes (1 mL L^{-1}), which were grown shaking until an OD_{600} of 0.4 – 0.6 was reached. The cultures were cooled down to 18 °C and IPTG solution (400 mM, 1 mL L^{-1}) was added to induce protein expression. The cultures were shaken overnight and harvested by centrifugation (3.600 x g, 30 min), the pelleted cells were resuspended in binding buffer (10 mL L^{-1} culture; 20 mM Na_2HPO_4, 500 mM NaCl, 20 mM imidazole, 1 mM $MgCl_2$, pH = 7.4, 4 °C)and lysed by ultra-sonication (50 % power, 5 x 1 min). The cell debris was removed by centrifugation (14.610 x g, 2 x 7 min), the supernatant was filtered through a syringe filter

and loaded onto a Ni^{2+}-NTA affinity chromatography column (Protino™ Ni-NTA, Macherey-Nagel, Düren, Germany). The column was washed twice with binding buffer (10 mL L^{-1} culture) and the desired His_6-tagged protein was eluted using elution buffer (10 mL L^{-1} culture; 20 mM Na_2HPO_4, 500 mM NaCl, 500 mM imidazole, 1 mM $MgCl_2$, pH = 7.4, 4 °C).

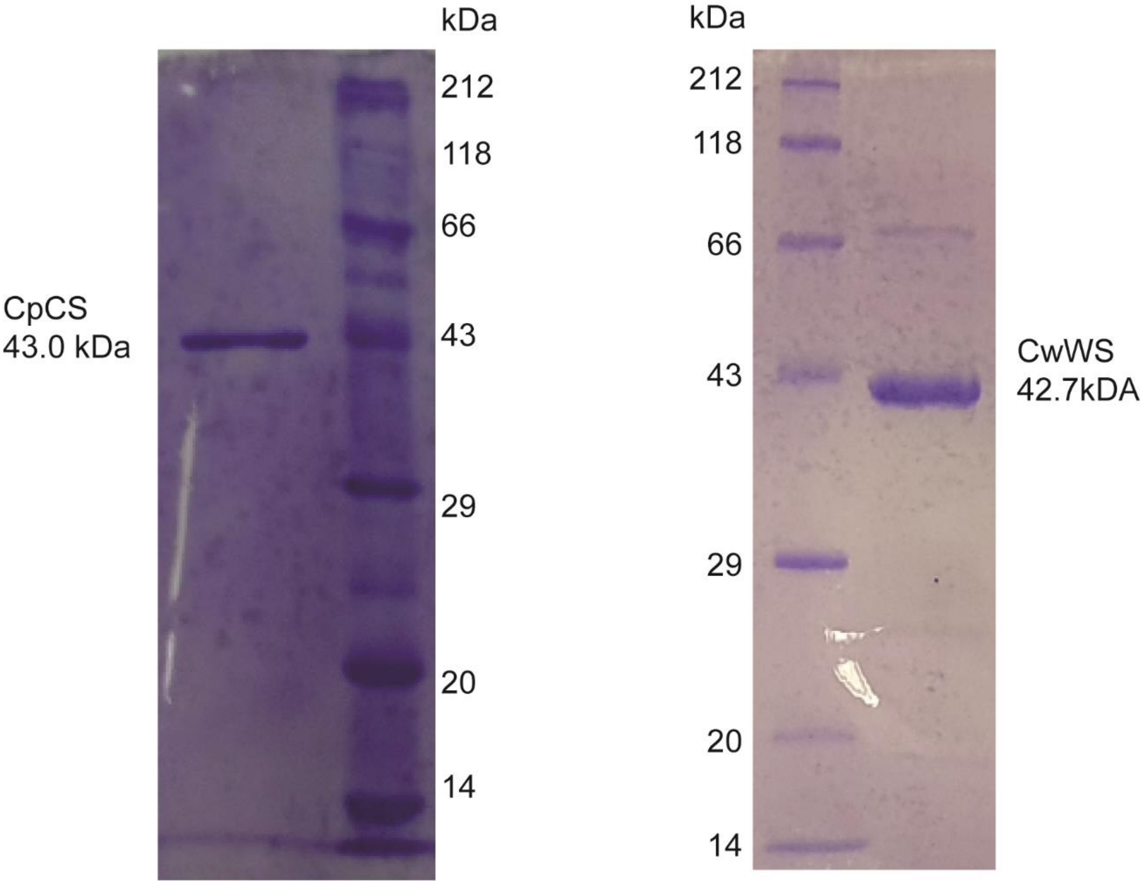

Figure S2. SDS-PAGE analysis of recombinant type I diterpene synthases from *Chryseobacterium polytrichastri* (CpCS) and from *Chryseobacterium wanjuense* (CwWS).

WP_073290622 (Chryseodiene Synthase, CpCS)

```
MIIFKDTKSIITKSQIMNIKNILENRFIYPFPDKISPFGKALQEIANTQWIDGELKDLLPLATREKYK
HTNTGIMSARWWPTTPTLDRMIPLSRFMLWAMYNDDMYEVATPNEIHFAQERSIAVLKGKISPEQAQI
PLAYQLAAIREDFLKFIPSESINRFADSLNEYFDGLEMEVLYQQNGTFPSVGNLISIRVRSLMVCAFV
DTIEIQTGITLPDRIYKHPVIKRLYHLSSSIIIYFNDVQSLHKDEVSGRLYFNLLGVLQHHYQLSKEE
ALEETIRMHNEDLEEFLLLKSTLPDFGEWQEAVIEIIECMGMFIKGWQTTSLLETKRYNNNGFPQIEE
LPKIE
```

WP_089795910 (Wanjudiene Synthase, CwWS)

```
MKTMTNEEFYAGLLDLPKPKYPFPDTIHPDFQRLREEYYNWIDTEYIIHSKQAREKHKEHNLCDIAAR
GCPFLKSIDDLLPLANYTANGAMMDDYFDRCSRNEMYEITNRIHELLTGNDPKEPSENGIFHLYWKLR
QDALRCDFPEHLYKRFVKSVDRVFKGYAEEKTYYRVNTIPPLPVYLLIREDTSGVQPYCDYVAMQKDY
RQIPDEIFDHPHIKRIQTLCSLLIGIHNDIISLPKELHREGDTMNIVKVLQQEYKITIQEAYIKALEL
HDDYLKEFLVLQNHLPSFDNWKNMIFEYIQDLGIMVSGVYAWHTDTVRYQNGNYVEGEYTNEK
```

Figure S3. Amino acid sequences of characterised type I terpene synthases from *Chryseobacterium polytrichastri* (CpCS) and from *Chryseobacterium wanjuense* (CwWS). Highly conserved motifs are highlighted in yellow.[5–8]

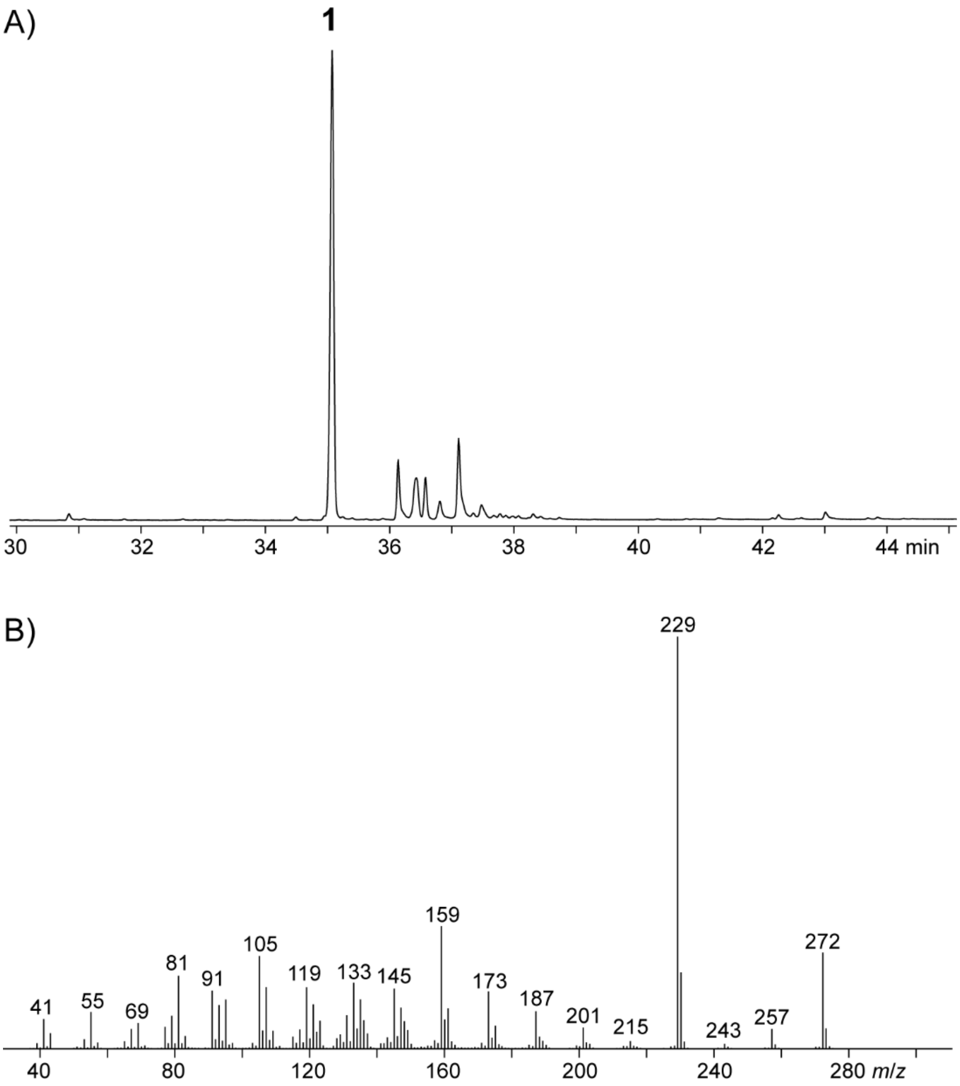

Figure S4. A) Total ion chromatogram of the products obtained from GGPP with CpFS. B) EI mass spectrum of the main product **1**.

Incubation experiments with recombinant CpCS and CwWS

Protein fractions obtained from Ni^{2+}-NTA purification were used directly for incubation experiments with geranylgeranyl diphosphate (0.5 mg mL^{-1}). The enzyme fractions were diluted with an equal volume of incubation buffer (50 mM TRIS, 10 mM $MgCl_2$, 20 % glycerol, pH 8.2) and filled to the final volume with binding buffer. Incubation was performed at 28 °C, shaking the reaction overnight. Initial trials with 1 mg GGPP were extracted with 200 µL *n*-hexane, which was dried and directly subjected to GC-MS. Preparative scale incubation was performed using 80 mg GGPP, followed by extraction with *n*-hexane (3 x 150 mL). The pooled organic fractions were dried with $MgSO_4$, evaporated under reduced pressure and subjected to column chromatography on silica gel using pentane to yield the respective terpenes as colourless oils.

Chryseodiene (3*S*,3a*S*,9*R*,10a*R*,*Z*)-3-isopropyl-6,9,10a-trimethyl-1,2,3,3a,4,7,8,9,10,10a-decahydrodicyclopenta[*a*,*d*][12]annulene (1). Colourless oil. Yield: 3.5 mg (0.01 mmol, 8 %). R_f (pentane) = 0.8, $[\alpha]_D^{20}$ = + 83.8 (*c* 0.39, C_6D_6). HRMS (EI): *m*/*z* = 272.2498 (calc. for $[C_{20}H_{32}]^+$ 272.2499. GC (HP5-MS): *I* = 1870. MS (EI, 70 eV): *m*/*z* (%) = 272 (21), 257 (5), 243 (1), 229 (100), 215 (2), 201 (5), 187 (9), 173 (14), 159 (32), 145 (16), 133 (18), 119 (17), 105 (26), 91 (16), 81 (22), 69 (8), 55 (12), 41 (9), see Figure S4. IR (diamond ATR): $\tilde{\nu}$ / cm^{-1} = 2955 (s), 2926 (s), 2872 (m), 1706 (w), 1620 (w), 1377 (w), 1260 (m), 1094 (m), 1025 (m), 800 (s). NMR data are given in Table S2 and Figures S5 – S11.

Wanjudiene (7a*S*,8*S*,11*R*,11a*S*)-8-isopropyl-3,6,11-trimethyl-2,3,5,7a,8,9,10,11-octahydro-1*H*-benzo[*d*]azulene (8). Colourless oil. Yield: 1.9 mg (0.01 mmol, 6 %). R_f (pentane) = 0.75, $[\alpha]_D^{20}$ = + 113.7 (*c* 0.21, C_6D_6). HRMS (EI): *m*/*z* = 272.2499 (calc. for $[C_{20}H_{32}]^+$ 272.2499. GC (HP5-MS): *I* = 1945. MS (EI, 70 eV): *m*/*z* (%) = 272 (38), 257 (11), 243 (2), 229 (63), 215 (7), 201 (10), 187 (35), 173 (27), 159 (100), 145 (51), 131 (30), 119 (56), 105 (69), 91 (30), 81 (26), 69 (18), 55 (24), 41 (21), see Figure S19. IR (diamond ATR): $\tilde{\nu}$ / cm^{-1} = 2954 (m), 2922 (s), 2854 (m), 1461 (m), 1373 (m), 1196 (w), 1154 (w), 1070 (w), 1015 (w), 918 (w), 893 (w), 839 (w), 800 (w), 720 (w). NMR data are given in Table S4 and Figures S20 – S26.

Table S2. NMR data of chryseodiene (**1**) in C_6D_6 recorded at 298 K.

C[a]		^1H[b]	^{13}C[b]
1	CH_2	2.25 (d, ^2J = 13.1, 1 H, H_β)	38.7
		1.93 (d, ^2J = 13.1, 1 H, H_α)	
2	C_q	–	142.2
3	CH	2.64 (m, 1 H)	45.6
4	CH_2	2.04 (m, 1 H, H_β)	31.7
		1.45 (m, 1 H, H_α)	
5	CH_2	2.63 (m, 1 H, H_α)	33.6
		2.19 (m, 1 H, H_β)	
6	C_q	–	137.1
7	C_q	–	134.1 (br)
8	CH	5.67 (tq, ^3J = 8.2, ^4J = 1.3, 1 H)	128.3
9	CH_2	1.96 (ddd, ^2J = 13.4, ^3J = 8.6, 8.6, 1 H, H_β)	25.6 (br)
		1.82 (dd, ^2J = 13.4, ^3J = 8.2, 1 H, H_α)	
10	CH	1.10 (m, 1 H)	53.3 (br)
11	C_q	–	40.9
12	CH_2	1.35 (ddd, ^2J = 12.2, ^3J = 8.6, 1.4, 1 H, H_β)	40.1
		1.21 (m, 1 H, H_α)	
13	CH_2	1.63 (m, 1 H, H_β)	23.1
		1.38 (m, 1 H, H_α)	
14	CH	1.50 (m, 1 H)	46.8
15	CH	1.72 (dsept, ^3J = 4.6, 6.7, 1 H)	29.0
16	CH_3	0.92 (d, ^3J = 6.9, 3 H)	22.5
17	CH_3	0.82 (d, ^3J = 6.7, 3 H)	17.2
18	CH_3	0.84 (s, 3 H)	18.2 (br)
19	CH_3	1.78 (br s, 3 H)	22.3 (br)
20	CH_3	1.01 (d, ^3J = 6.9, 3 H)	18.7 (br)

[a] Carbon numbering as shown in Scheme 1 of main text. [b] Chemical shifts δ in ppm, multiplicity: s = singlet, d = doublet, t = triplet, q = quartet, sept = septet, m = multiplet, br = broad, coupling constants *J* are given in Hertz.

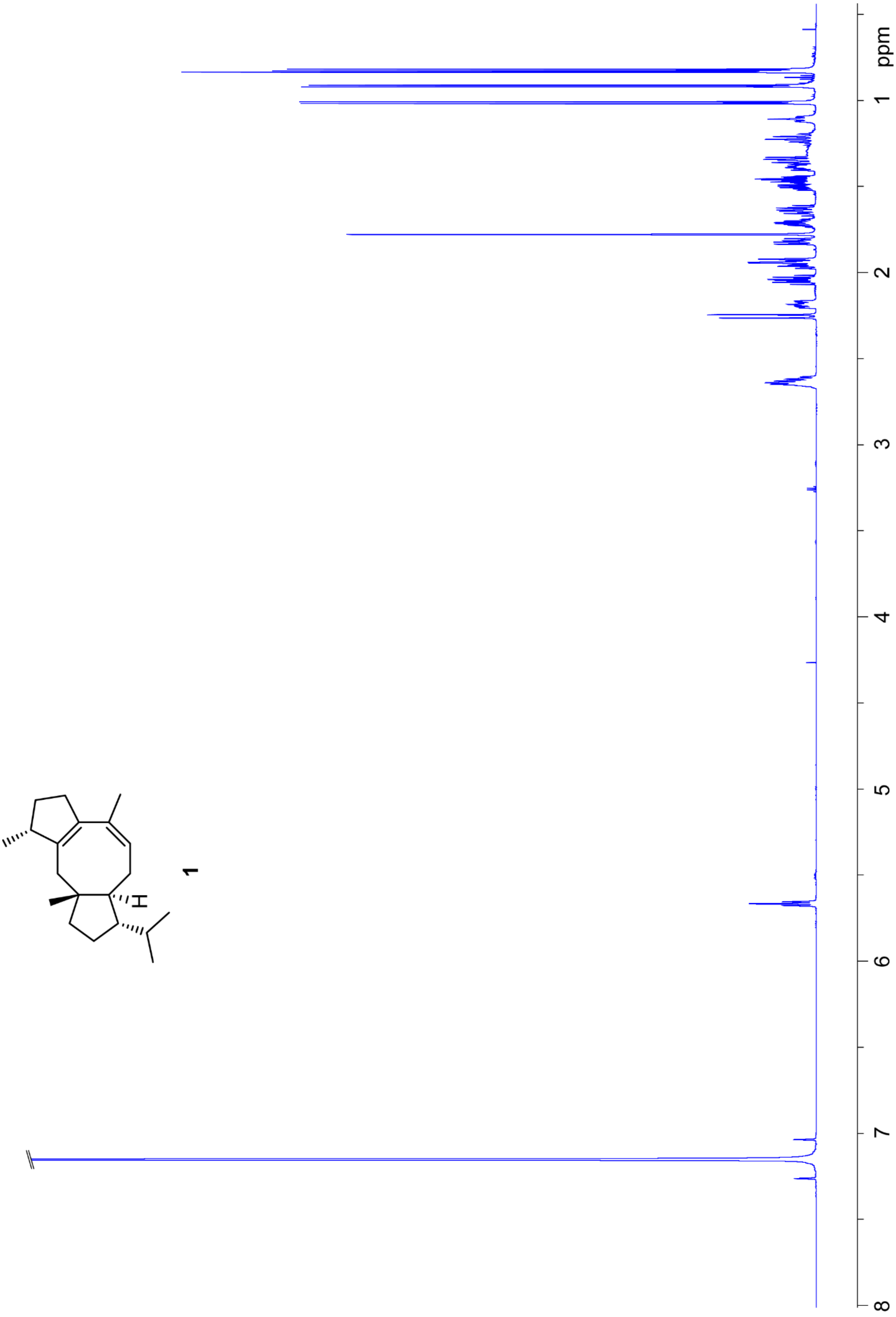

Figure S5. ^{1}H-NMR spectrum of **1** (700 MHz, C_6D_6).

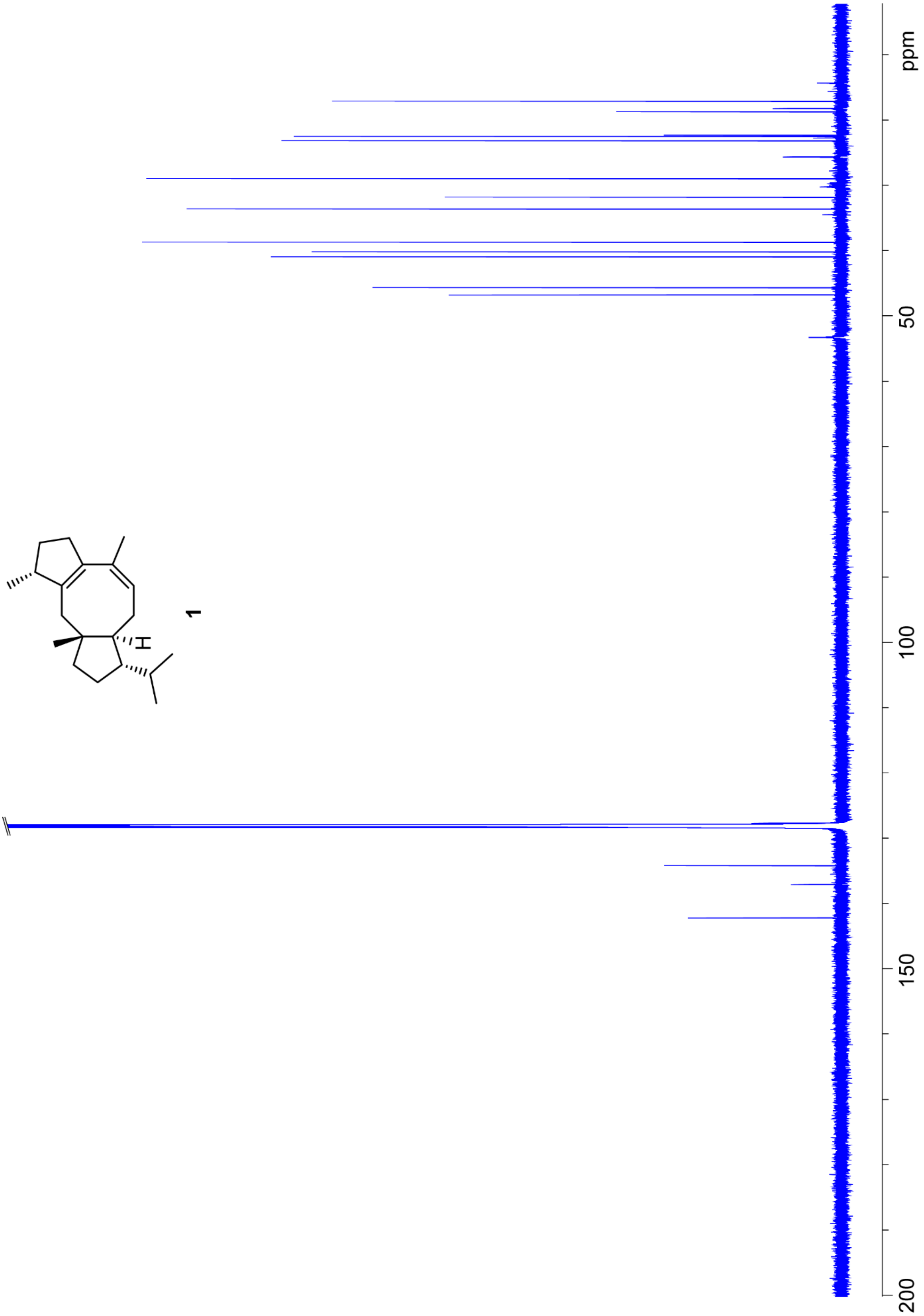

Figure S6. ^{13}C-NMR spectrum of **1** (175 MHz, C_6D_6).

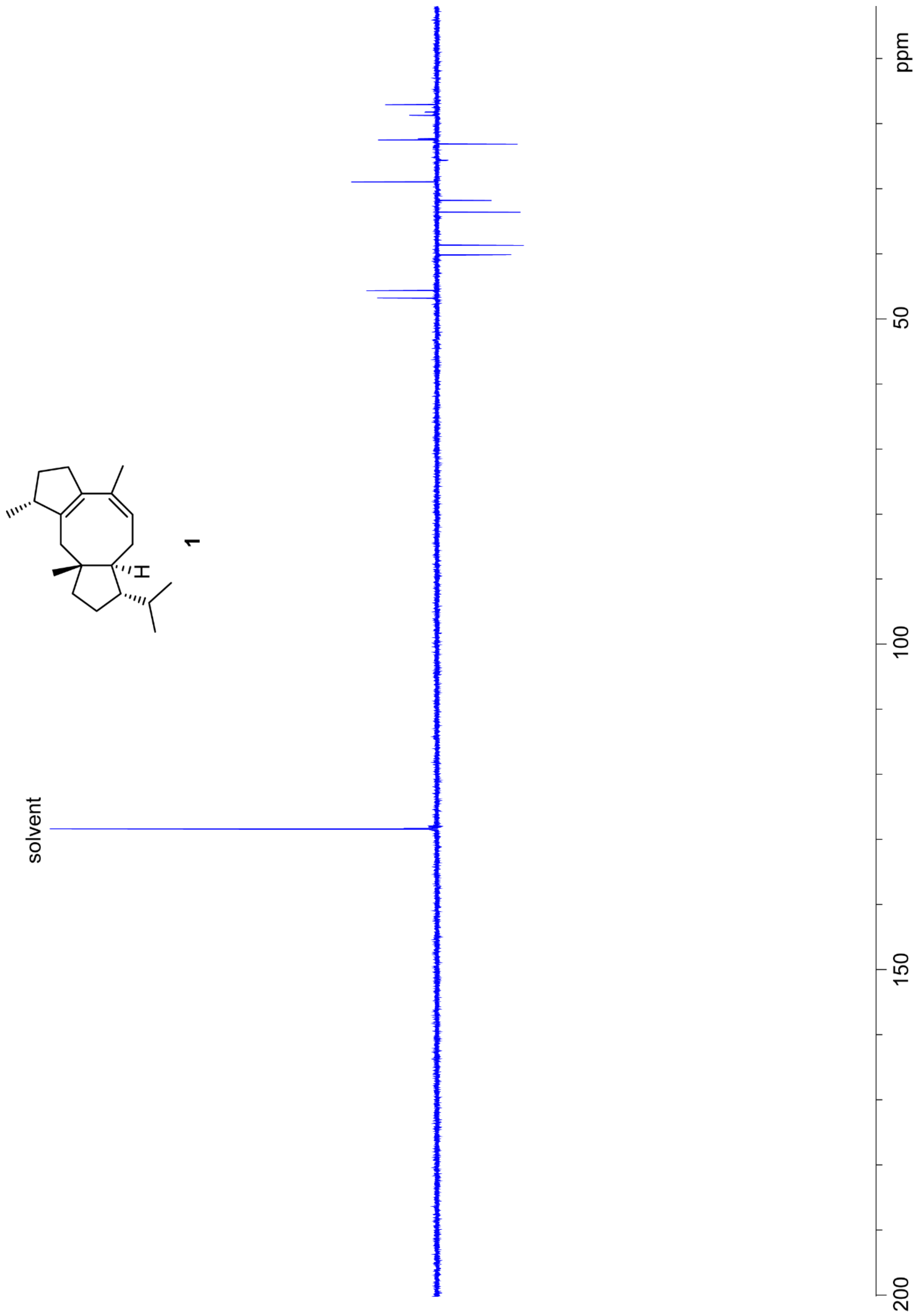

Figure S7. ^{13}C-DEPT-135 spectrum of **1** (175 MHz, C_6D_6).

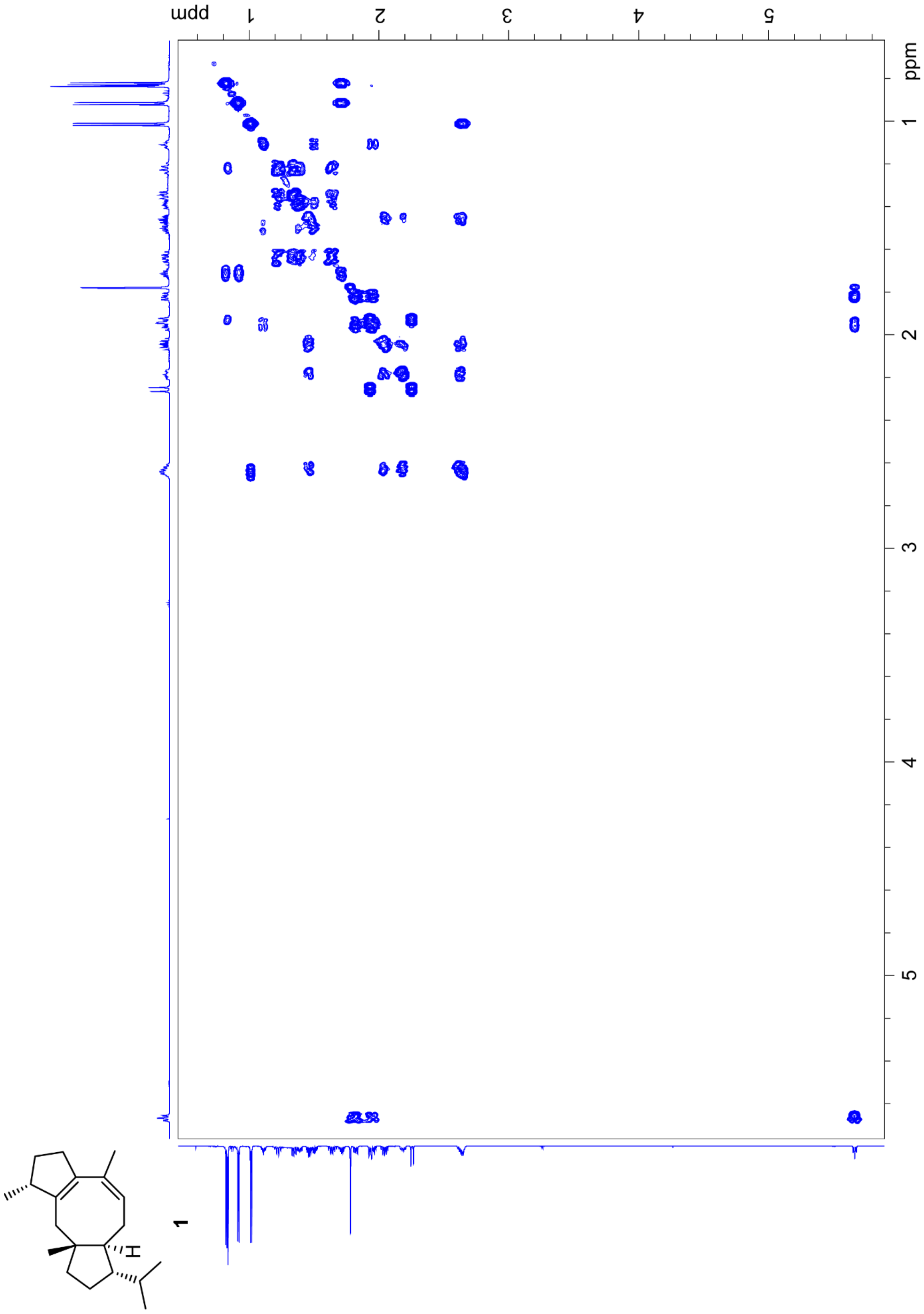

Figure S8. ^{1}H,^{1}H-COSY spectrum of **1** (C_6D_6).

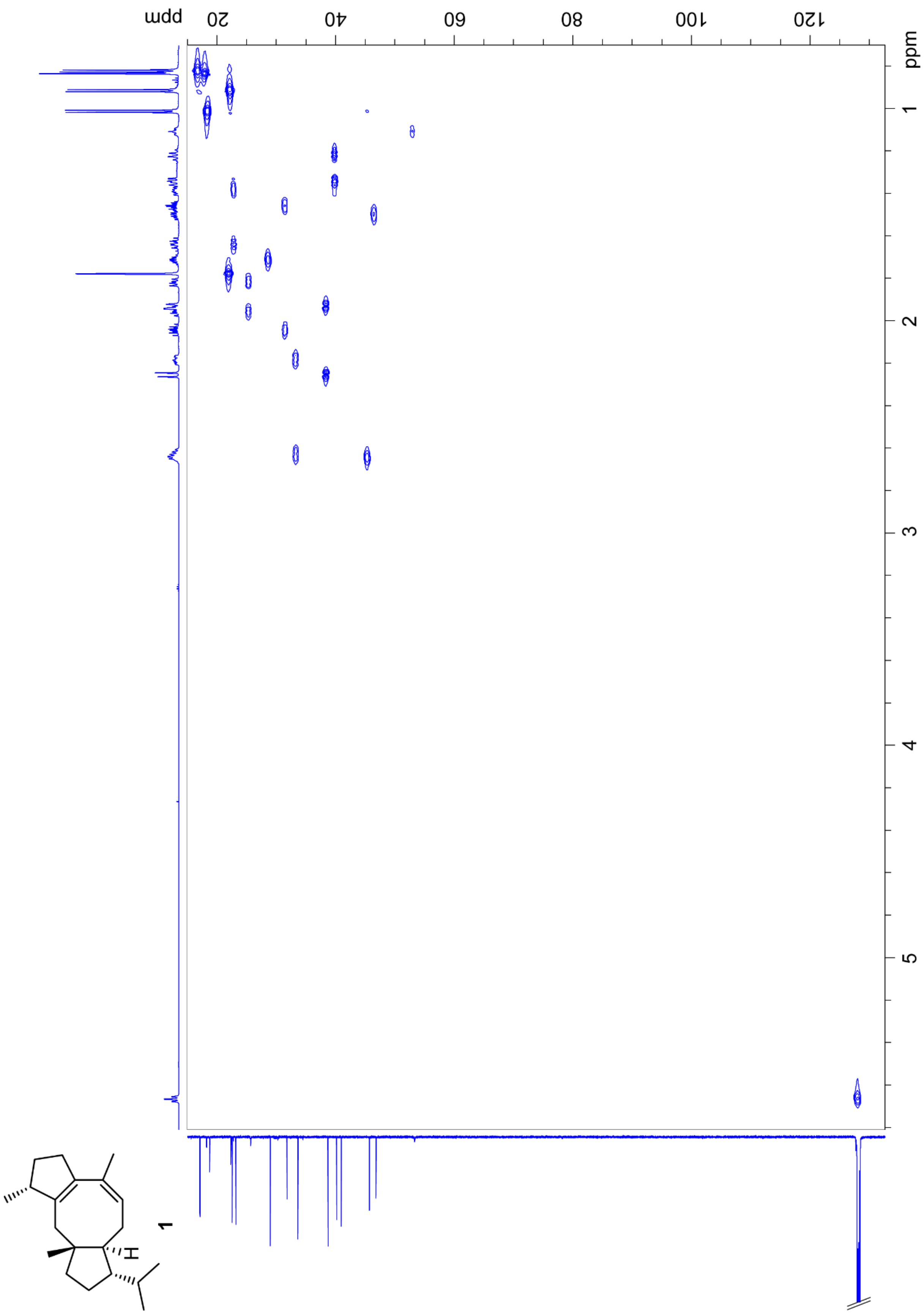

Figure S9. HSQC spectrum of **1** (C_6D_6).

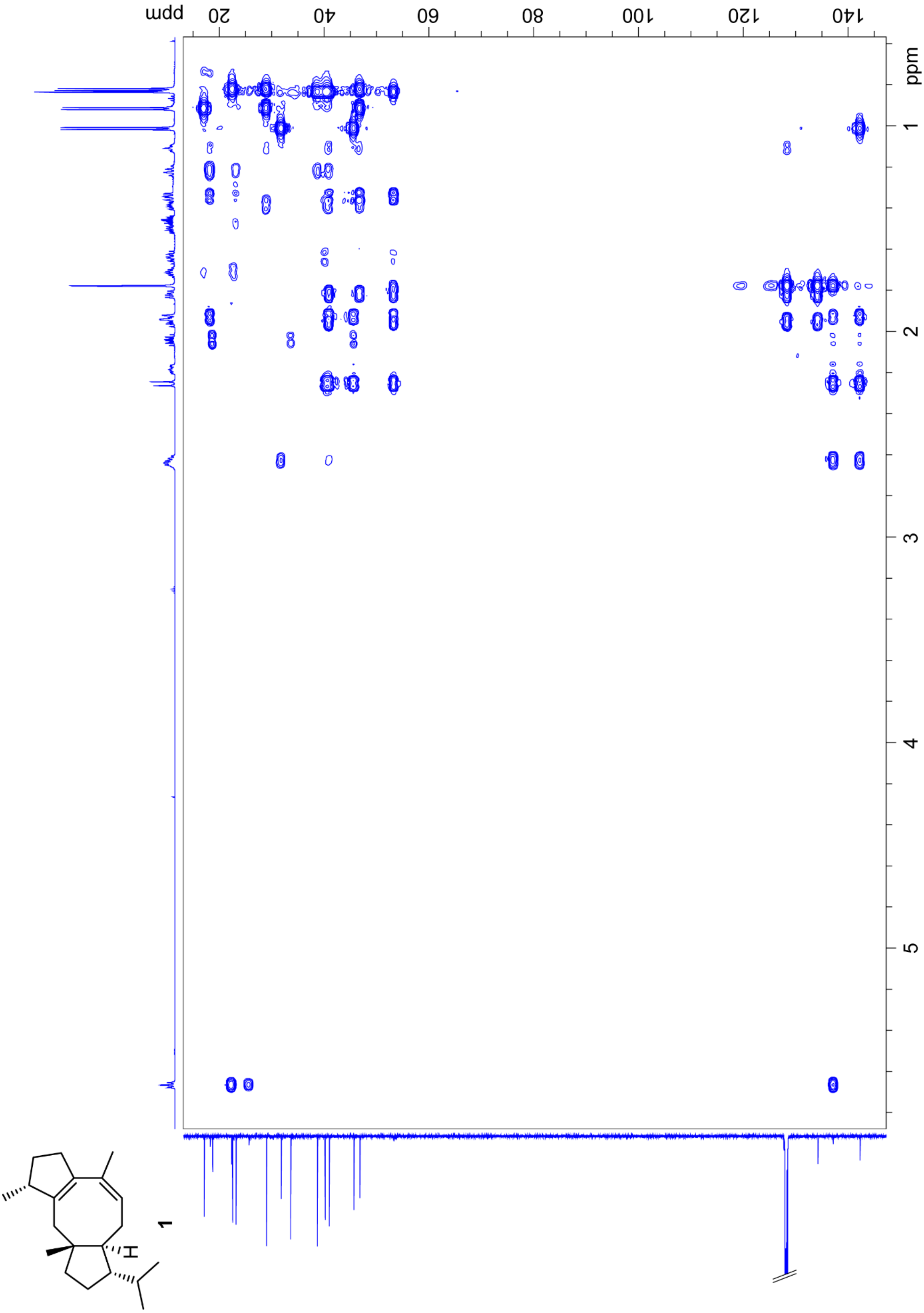

Figure S10. HMBC spectrum of **1** (C_6D_6).

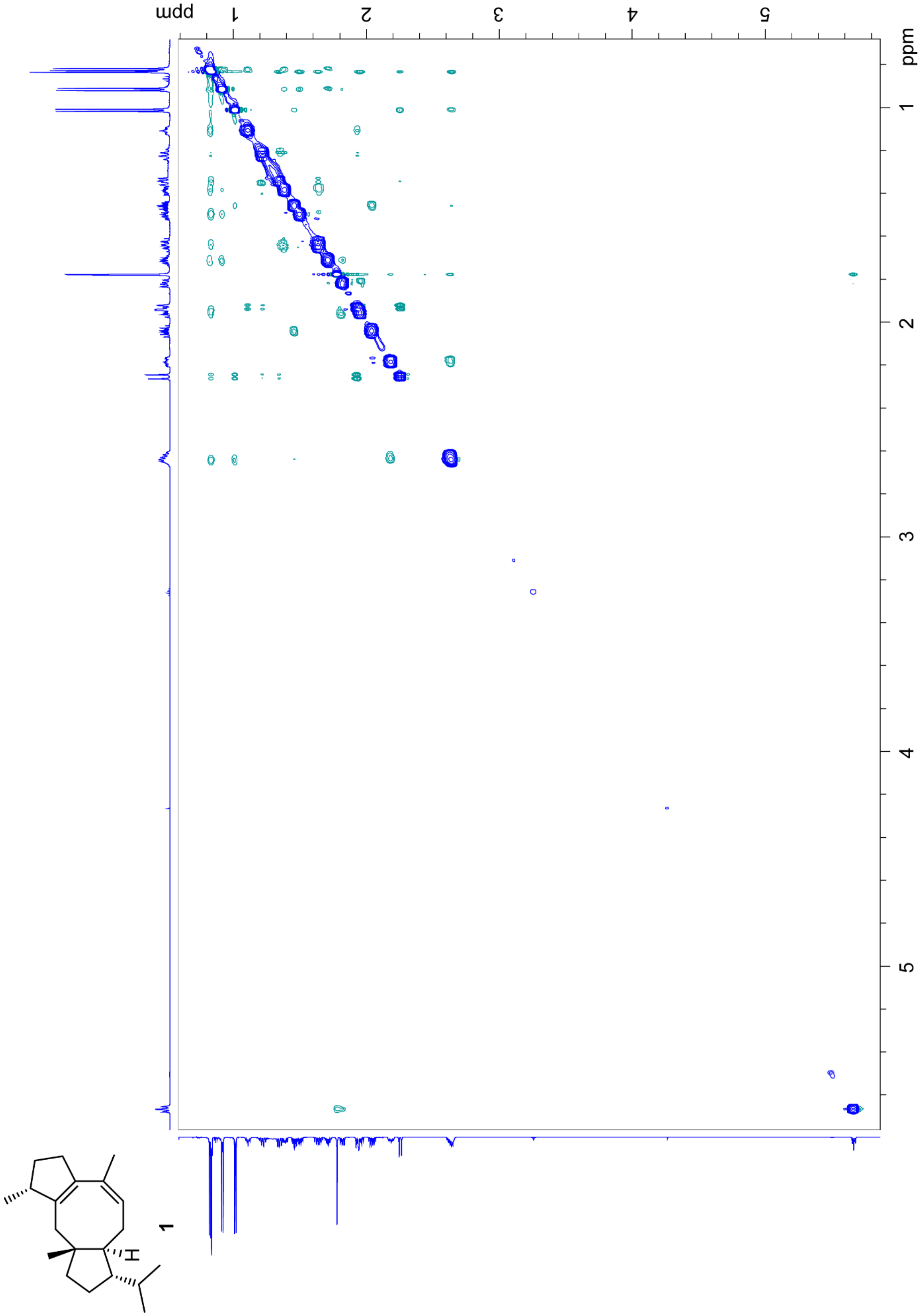

Figure S11. NOESY spectrum of **1** (C_6D_6).

Incubation experiments with isotopically labelled substrates and CpCS

Isotopic labelling experiments were performed with approximately 1 mg labelled GGPP (or its precursors) in substrate buffer (1 mL, 25 mM NH_4HCO_3), incubation buffer (4 mL), enzyme elution fractions (3 mL) with the substrates and enzyme preparations as listed in Table S3. After incubation with shaking at 28 °C overnight the products were extracted with C_6D_6 (650 μL) or *n*-hexane (200 μL) for NMR or GC/MS experiments, respectively. The extracts were dried with $MgSO_4$ and analysed by NMR and/or GC/MS.

Table S3. Isotopic labelling experiments with CpCS.

entry	substrate(s)	additional enzyme(s)	results shown in
1	DMAPP + (*E*)-(4-^{13}C,4-^{2}H)IPP[9]	FPPS[10] + GGPPS[11]	Figures S12, S15, S16
2	DMAPP + (*Z*)-(4-^{13}C,4-^{2}H)IPP[9]	FPPS[10] + GGPPS[11]	Figures S12, S15, S16
3	(*R*)-(1-^{13}C,1-^{2}H)IPP[12]	FPPS[10] + GGPPS[11] + IDI[13]	Figure S13
4	(*S*)-(1-^{13}C,1-^{2}H)IPP[12]	FPPS[10] + GGPPS[11] + IDI[13]	Figure S13
5	FPP + (1-^{13}C)IPP[11]	GGPPS[11]	Figure S14
6	FPP + (2-^{13}C)IPP[14]	GGPPS[11]	Figure S14
7	FPP + (3-^{13}C)IPP[11]	GGPPS[11]	Figure S14
8	FPP + (4-^{13}C)IPP[11]	GGPPS[11]	Figure S14
9	(1-^{13}C)FPP[15] + IPP	GGPPS[11]	Figure S14
10	(2-^{13}C)FPP[15] + IPP	GGPPS[11]	Figure S14
11	(3-^{13}C)FPP[15] + IPP	GGPPS[11]	Figure S14
12	(4-^{13}C)FPP[15] + IPP	GGPPS[11]	Figure S14
13	(5-^{13}C)FPP[15] + IPP	GGPPS[11]	Figure S14
14	(6-^{13}C)FPP[15] + IPP	GGPPS[11]	Figure S14
15	(7-^{13}C)FPP[15] + IPP	GGPPS[11]	Figure S14
16	(8-^{13}C)FPP[15] + IPP	GGPPS[11]	Figure S14
17	(9-^{13}C)FPP[15] + IPP	GGPPS[11]	Figure S14
18	(10-^{13}C)FPP[15] + IPP	GGPPS[11]	Figure S14
19	(11-^{13}C)FPP[15] + IPP	GGPPS[11]	Figure S14
20	(12-^{13}C)FPP[15] + IPP	GGPPS[11]	Figure S14
20	(9-^{13}C)GPP[16] + IPP	GGPPS[11]	Figure S14
21	(10-^{13}C)GPP[17] + IPP	GGPPS[11]	Figure S14
22	(15-^{13}C)FPP[15]	GGPPS[11]	Figure S14
23	(20-^{13}C)GGPP[11]	–	Figure S14
24	(3-^{13}C,2-^{2}H)GGPP[11]	–	Figure S17
25	(2-^{2}H)FPP[11] + IPP	GGPPS[11]	Figure S18

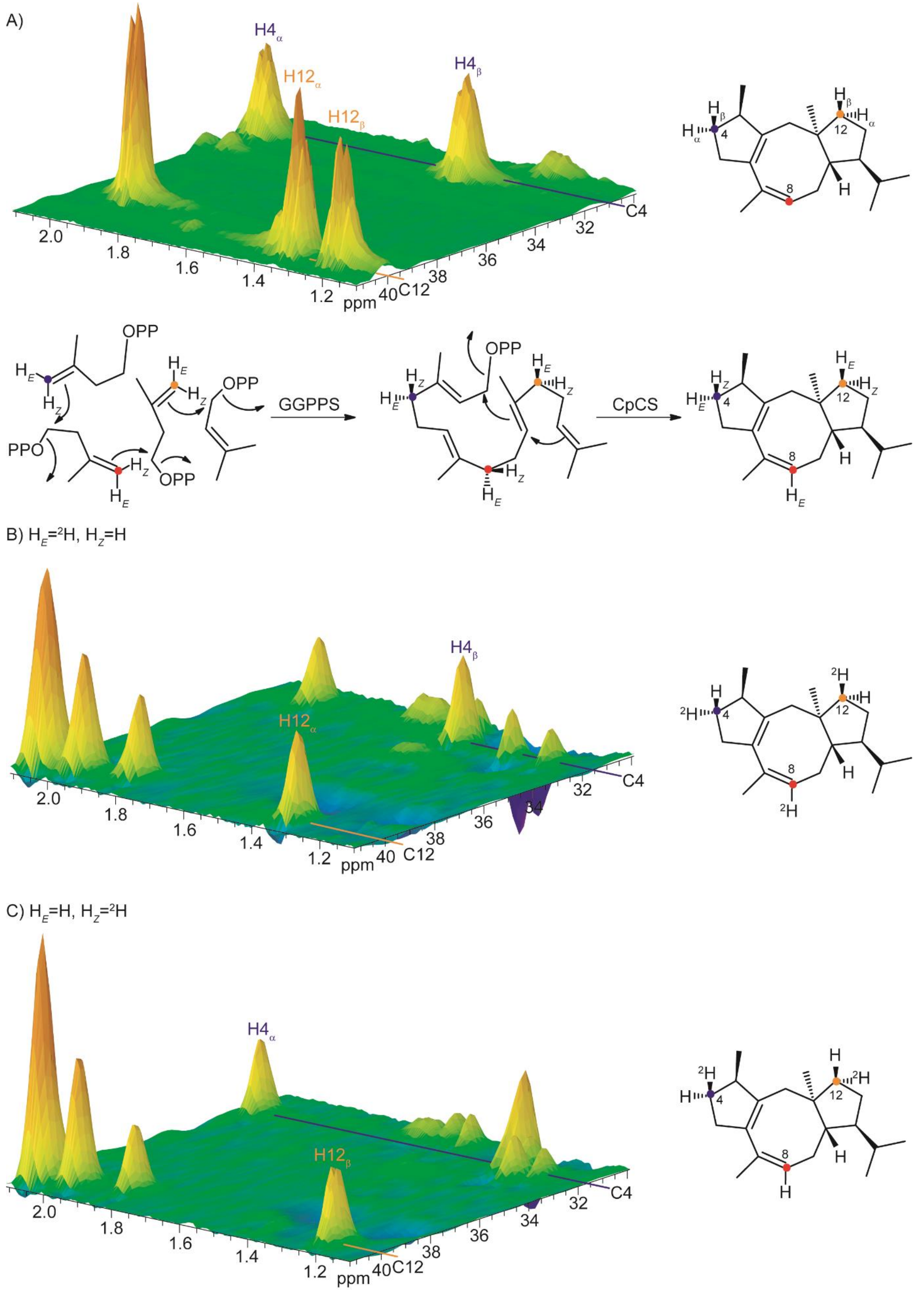

Figure S12. Determination of the absolute configuration of **1**. A) Partial HSQC of unlabelled **1**. Incubation of CpCS with DMAPP, GGPPS and B) (*E*)(4-^{2}H,4-^{13}C)IPP and C) (*Z*)(4-^{2}H,4-^{13}C)IPP. Coloured dots indicate ^{13}C labelling.

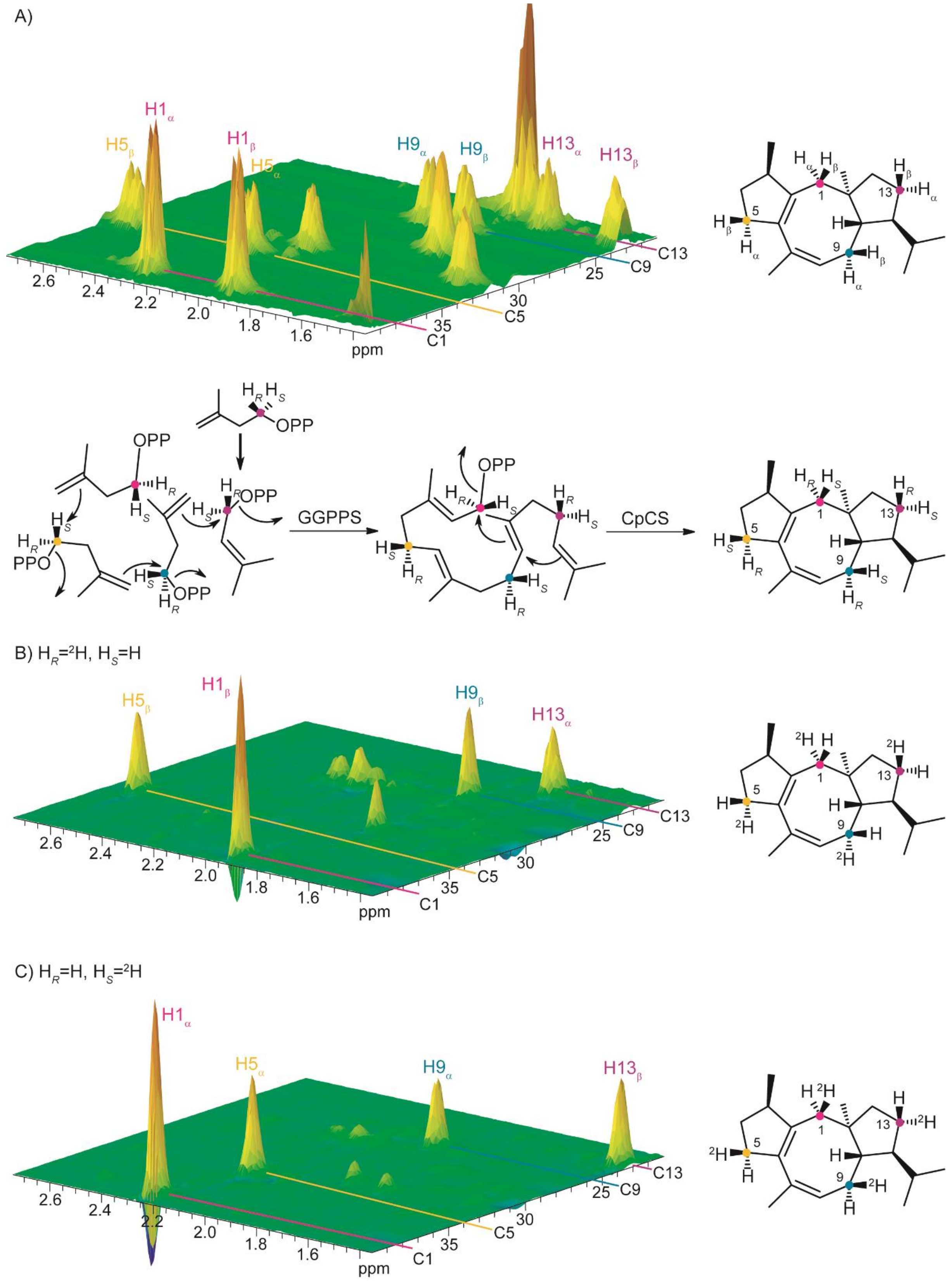

Figure S13. Determination of the absolute configuration of **1**. A) Partial HSQC of unlabelled **1**. Incubation of CpCS with DMAPP, IDI, GGPPS and B) (*R*)(1-^{2}H,1-^{13}C)IPP and C) (*S*)(1-^{2}H,1-^{13}C)IPP. Coloured dots indicate ^{13}C labelling.

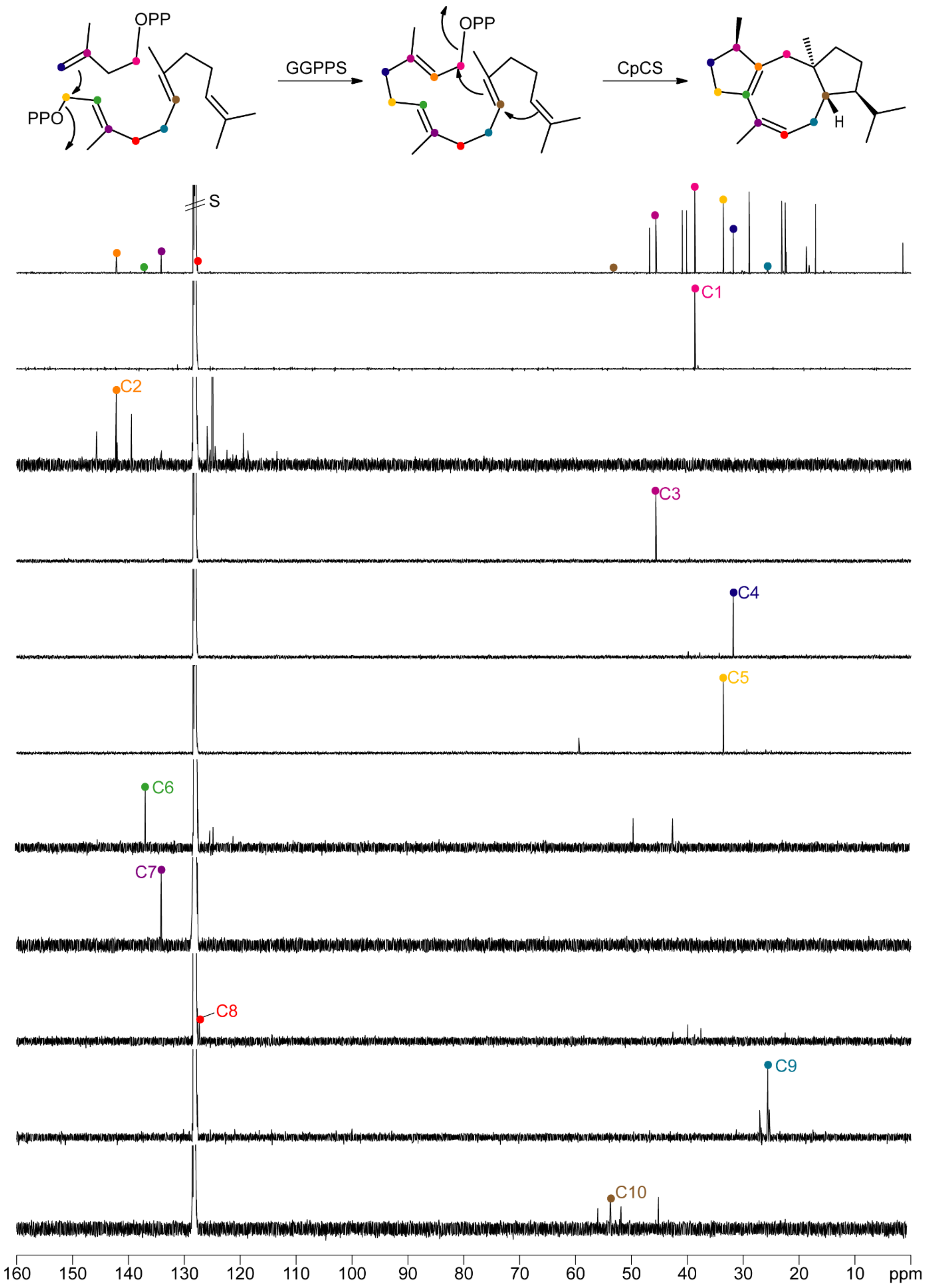

Figure S14. ^{13}C-NMR spectra of **1** obtained from incubation of CpCS with either (^{13}C)GGPP isotopomers or isotopomers prepared in situ from (^{13}C)IPP and FPP or IPP and (^{13}C)FPP with GGPPS..

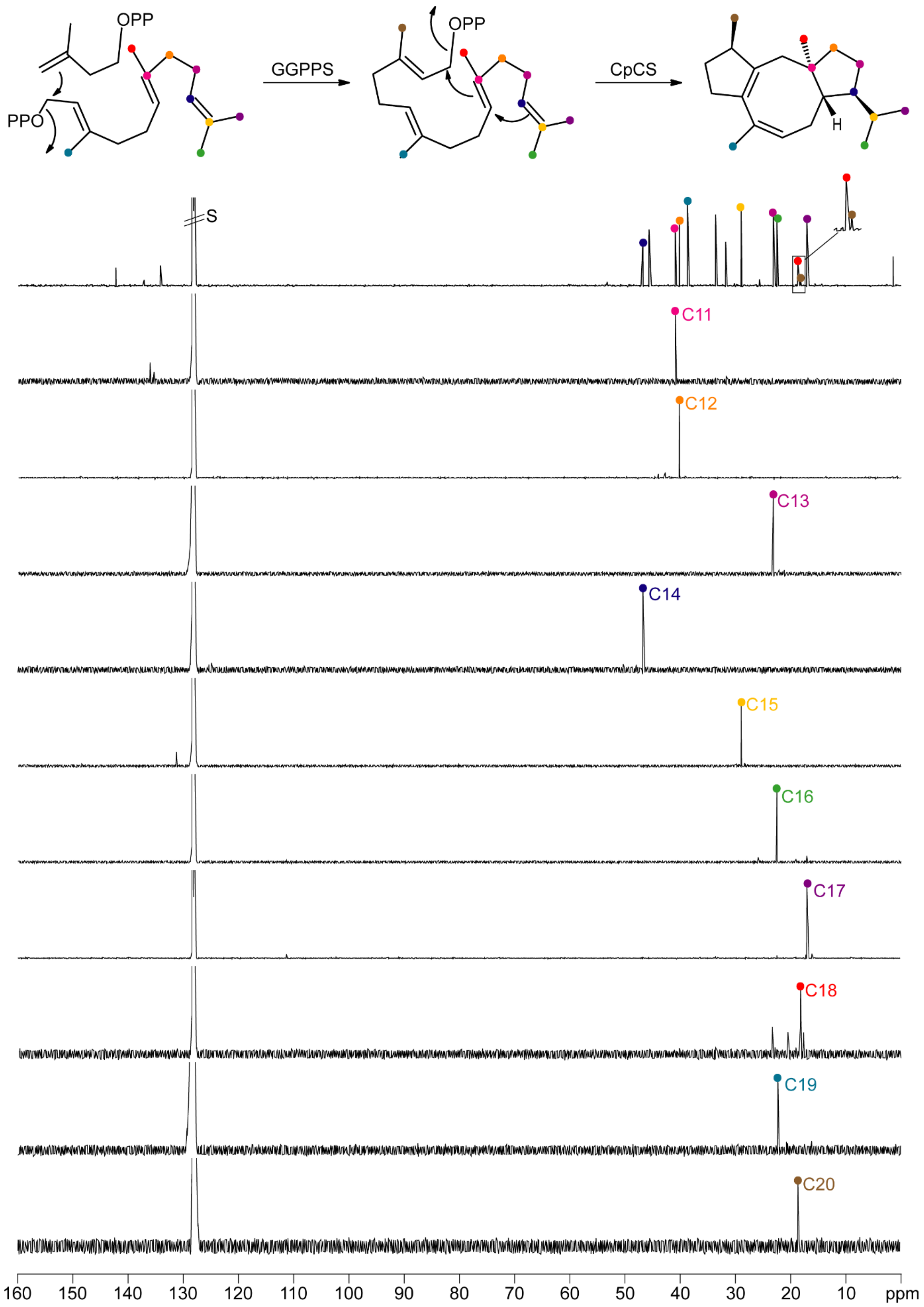

Figure S14 (continued). ^{13}C-NMR spectra of **1** obtained from incubation of CpCS with either (^{13}C)GGPP isotopomers or isotopomers prepared in situ from (^{13}C)IPP and FPP or IPP and (^{13}C)FPP with GGPPS.

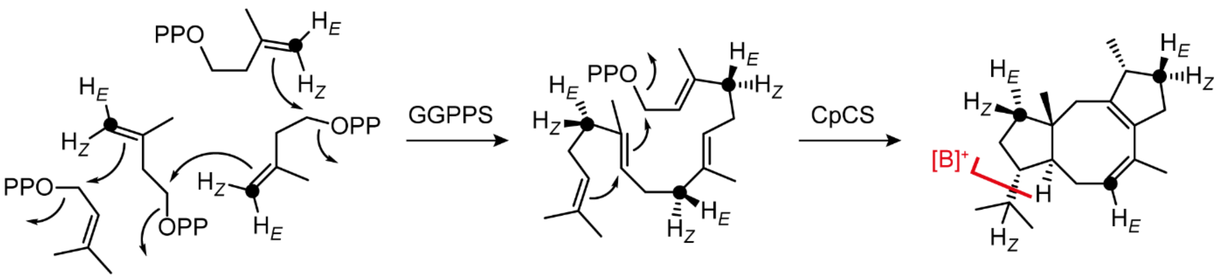

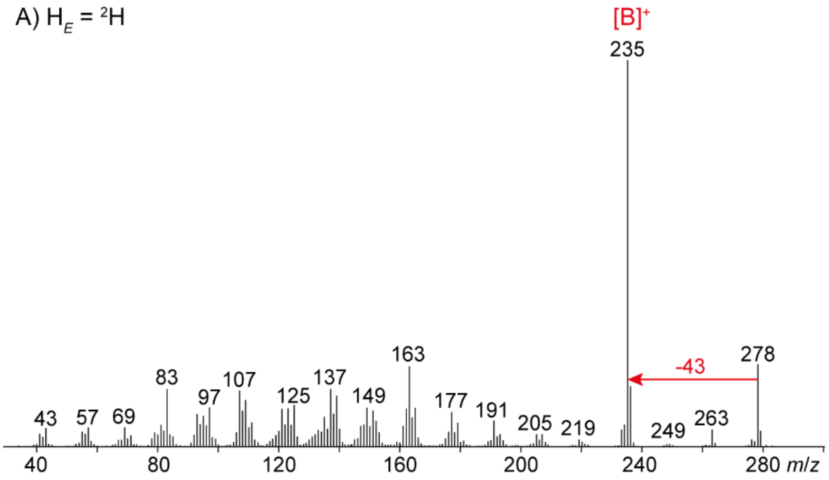

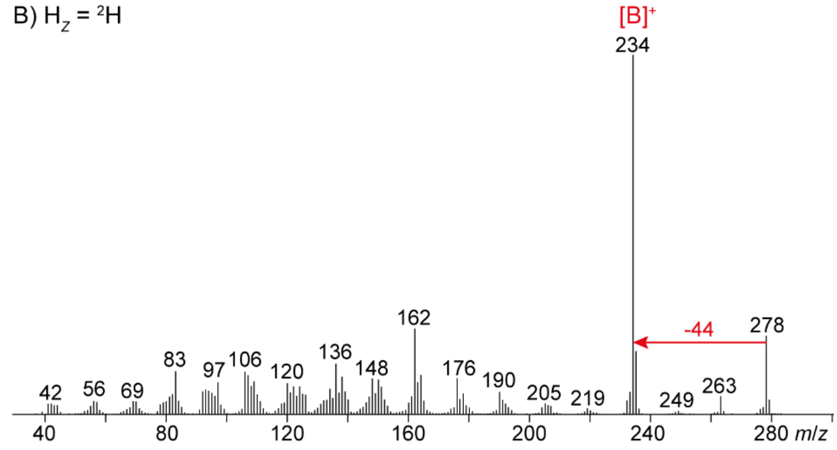

Figure S15. Investigation of the 1,5-hydride shift during the cyclisation of GGPP to **1**. A) EI mass spectrum of the product obtained from an incubation of DMAPP and (*E*)-(4-^{13}C,4-^{2}H)IPP with GGPPS and CpCS. The base peak ion [B]$^+$ arises by cleavage of a non-labelled *i*Pr group. B) EI mass spectrum of the product obtained from an incubation of DMAPP and (*Z*)-(4-^{13}C,4-^{2}H)IPP with GGPPS and CpCS. The base peak ion [B]$^+$ arises by cleavage of a deuterium containing *i*Pr group, which supports the proposed 1,5-hydride shift. This hydride shift proceeds with the highly stereoselective migration of the 8-*pro-R* hydrogen.

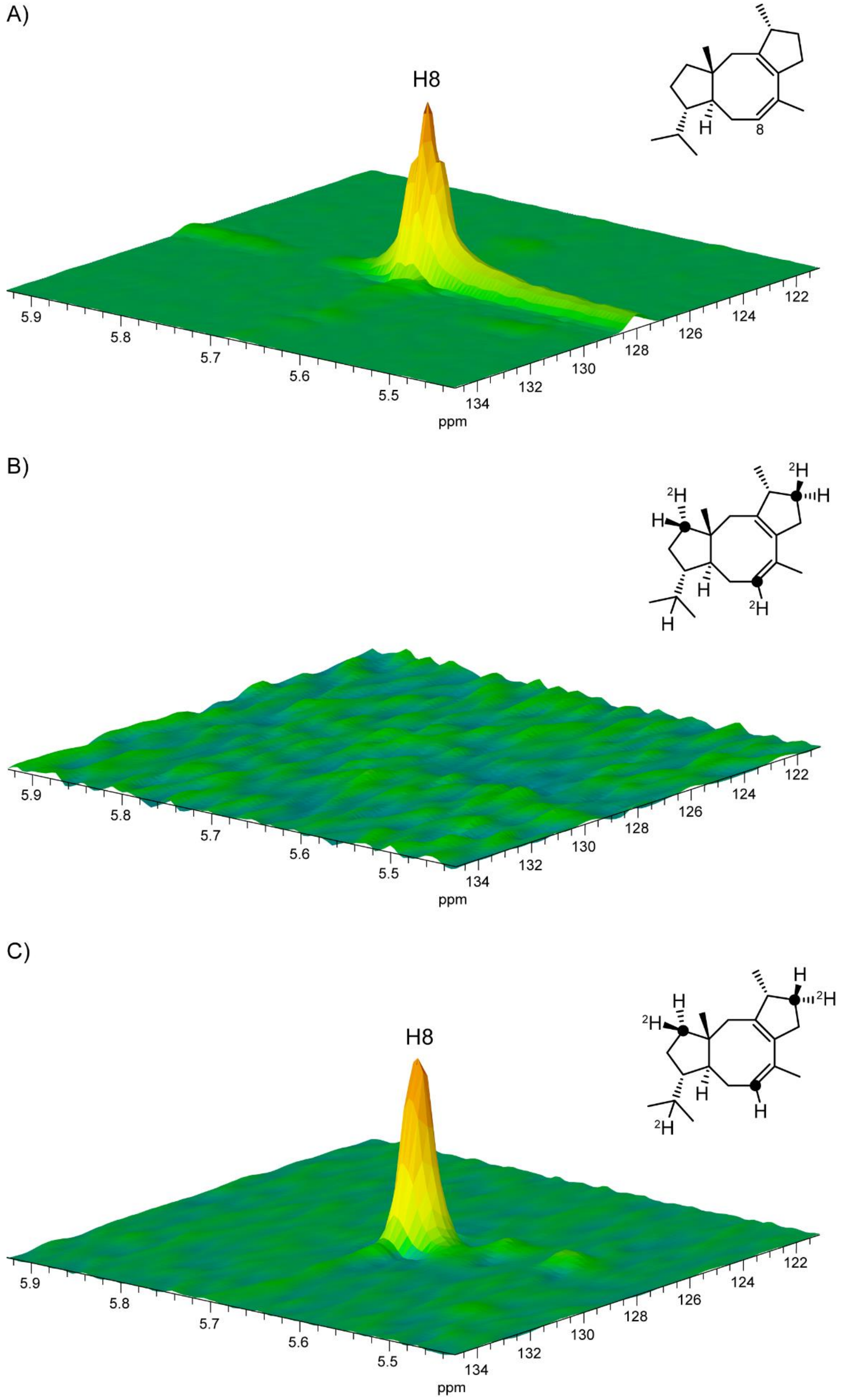

Figure S16. Partial HSQC spectra showing the region for H8 of A) unlabelled **1**, B) ($^{13}C_3$,$^{2}H_3$)-**1** obtained from (*E*)-(4-^{13}C,4-^{2}H)IPP, and C) ($^{13}C_3$,$^{2}H_3$)-**1** obtained from (*Z*)-(4-^{13}C,4-^{2}H)IPP. The vanished crosspeak in B) indicates a substitution of H8 by deuterium.

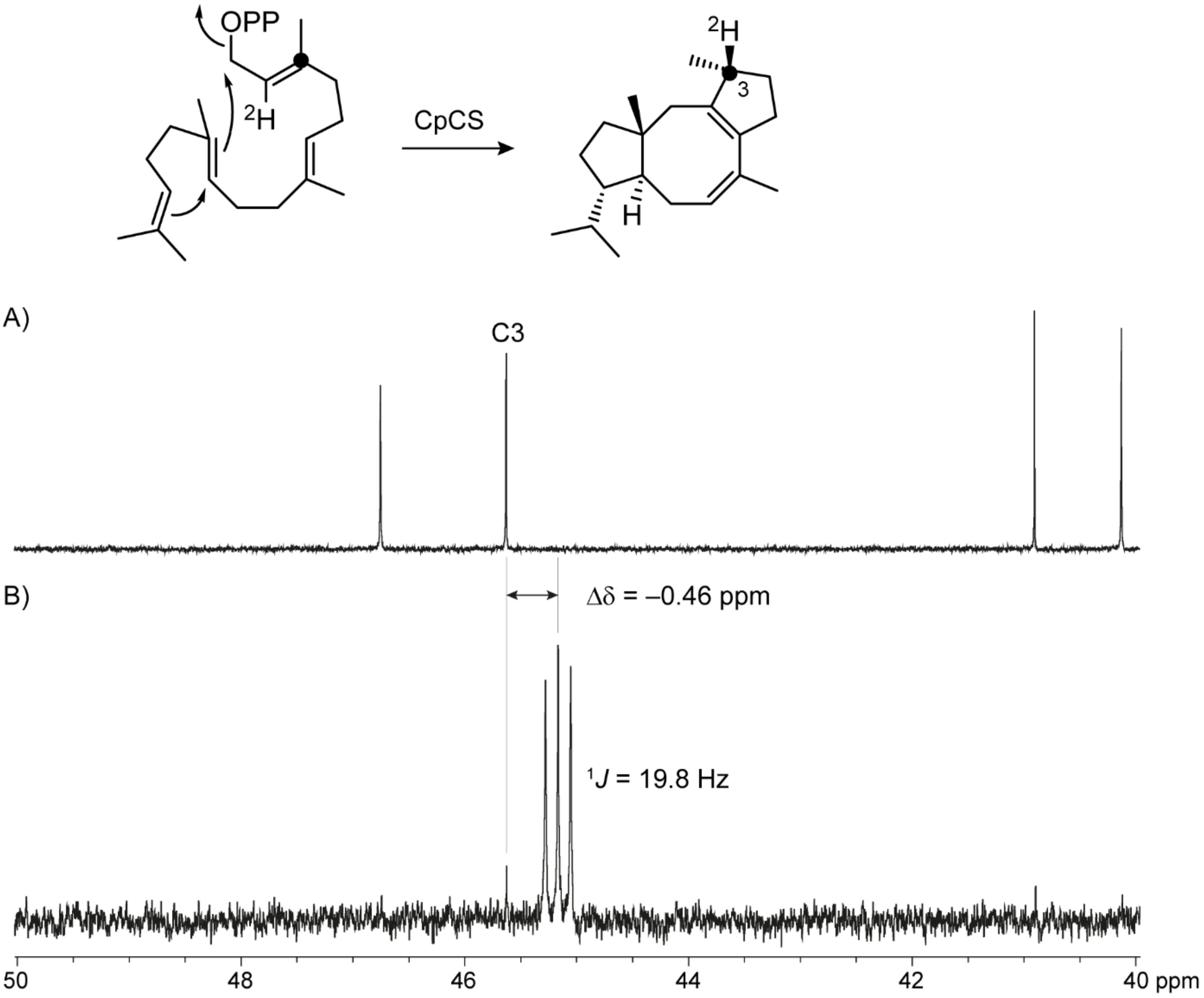

Figure S17. Investigation of the 1,2-hydride shift during the cyclisation of GGPP to **1**. A) Partial ^{13}C-NMR spectrum of unlabelled **1**. B) ^{13}C-NMR spectrum of the product (3-^{13}C,3-^{2}H)-**1** obtained by incubation of (3-^{13}C,2-^{2}H)GGPP with CpCS. The sligtly upfield shifted triplet for C3 indicates a direct ^{13}C-^{2}H bond which supports the proposed 1,2-hydride migration.

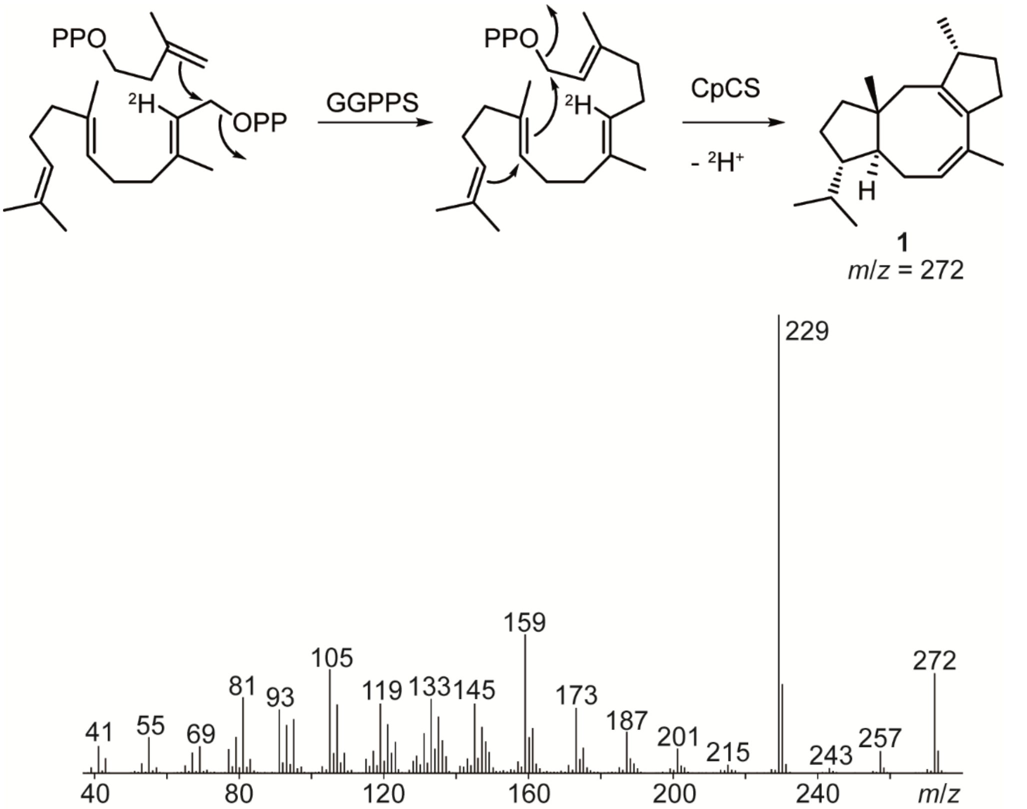

Figure S18. Investigation of the final deprotonation step in the formation of **1**. Mass spectrum of the product from incubation of CpCS with (2-^{2}H)FPP, IPP and GGPPS.

Computational details

All computed structures are geometry optimised without (symmetry) restrictions and are characterised as minima or as transition structures by frequency analyses (which also provides Gibbs-corrections) using the B97D3/6-31g(d,p) method, which includes Grimme's empirical dispersion correction[18] in Gaussian16.[19]

For single point energies, the mPW1PW91 functional is applied with the 6-311+G(d,p) basis set, as this method has shown to be reliable for examining carbocation cyclisation-rearrangement reactions.[20]

For an entropic quasi-harmonic treatment a frequency cut-off value of 100.0 wavenumbers is applied according to Grimme, using a mixture of RRHO and free-rotor vibrational entropies.[21]

For the cationic cascade reaction from **A** to **D** (cf. Figure 1) nine stationary points were localised and were characterised by frequency as well as IRC calculations, employing the B97D3/6-31G**-method. These nine structures are listed below with their energies, imaginary frequencies and cartesian coordinates. Gibbs-corrections (298.15 K, including an entropic quasi-harmonic correction) as wells as mPW1PW91/6-311+G(d,p)// B97D3/6-31G** single point energies are provided additionally.

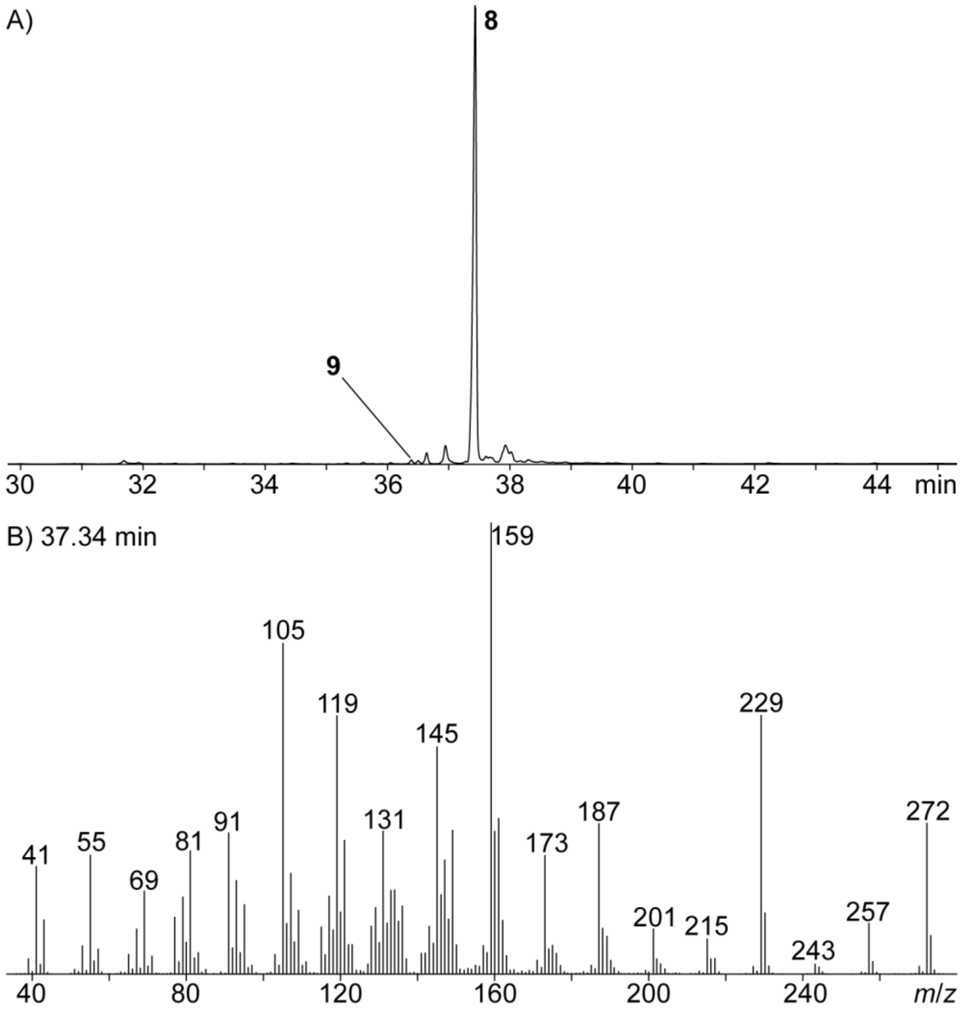

Figure S19. A) Total ion chromatogram of the products obtained from GGPP with CwWS. B) EI mass spectrum of the main product **8**.

Table S4. NMR data of wanjudiene (**8**) in C_6D_6 recorded at 298 K.

C[a]		^{1}H[b]	^{13}C[b]
1	CH	2.54 (br s, 1 H)	46.0
2	CH	5.34 (s, 1 H)	123.9
3	C_q	–	133.2
4	CH_2	2.91 (dm, $^{2}J_{H,H}$ = 20.3, 1 H, H_β)	33.2
		2.39 (dd, $^{2}J_{H,H}$ =20.3, $^{3}J_{H,H}$ = 8.8, 1 H, H_α)	
5	CH	5.50 (ddd, $^{3}J_{H,H}$ = 8.8, 3.2, $^{4}J_{H,H}$ = 2.8, 1 H)	117.3
6	C_q	–	155.6
7	CH	2.41 – 2.45 (m, 1 H)	39.9
8	CH_2	1.66 – 1.69 (m, 1 H, H_β)	34.7
		1.12 – 1.16 (m, 1 H, H_α)	
9	CH_2	1.97 (ddd, $^{2}J_{H,H}$ = 12.9, $^{3}J_{H,H}$ = 6.8; 1.0, 1 H, H_α)	34.2
		1.16 – 1.21 (m, 1 H, H_β)	
10	C_q	–	54.1
11	CH	2.35 – 2.39 (m, 1 H)	30.4
12	CH_2	1.52 – 1.57 (m, 1 H, H_α)	32.4
		1.16 – 1.26 (m, 1 H, H_β)	
13	CH_2	1.63 – 1.68 (m, 1 H, H_β)	26.4
		1.29 (dddd, $^{2}J_{H,H}$ = 13.0, $^{3}J_{H,H}$ = 12.9; 12.8; 4.3, 1 H, H_α)	
14	CH	1.07 (dddd, $^{3}J_{H,H}$ = 12.9; 9.6; 3.3; 3.3, 1 H)	45.5
15	CH	1.53 – 1.59 (m, 1 H)	29.1
16	CH_3	0.91 (d, $^{3}J_{H,H}$ = 6.7, 3 H)	20.8
17	CH_3	0.94 (d, $^{3}J_{H,H}$ = 6.5, 3 H)	22.0
18	CH_3	0.81 (d, $^{3}J_{H,H}$ = 6.9, 3 H)	19.0
19	CH_3	1.09 (d, $^{3}J_{H,H}$ = 6.9, 3 H)	17.9
20	CH_3	1.73 (s, 3 H)	28.4

[a] Carbon numbering as shown in Scheme 3 of main text. [b] Chemical shifts δ in ppm, multiplicity: s = singlet, d = doublet, m = multiplet, br = broad, coupling constants *J* are given in Hertz.

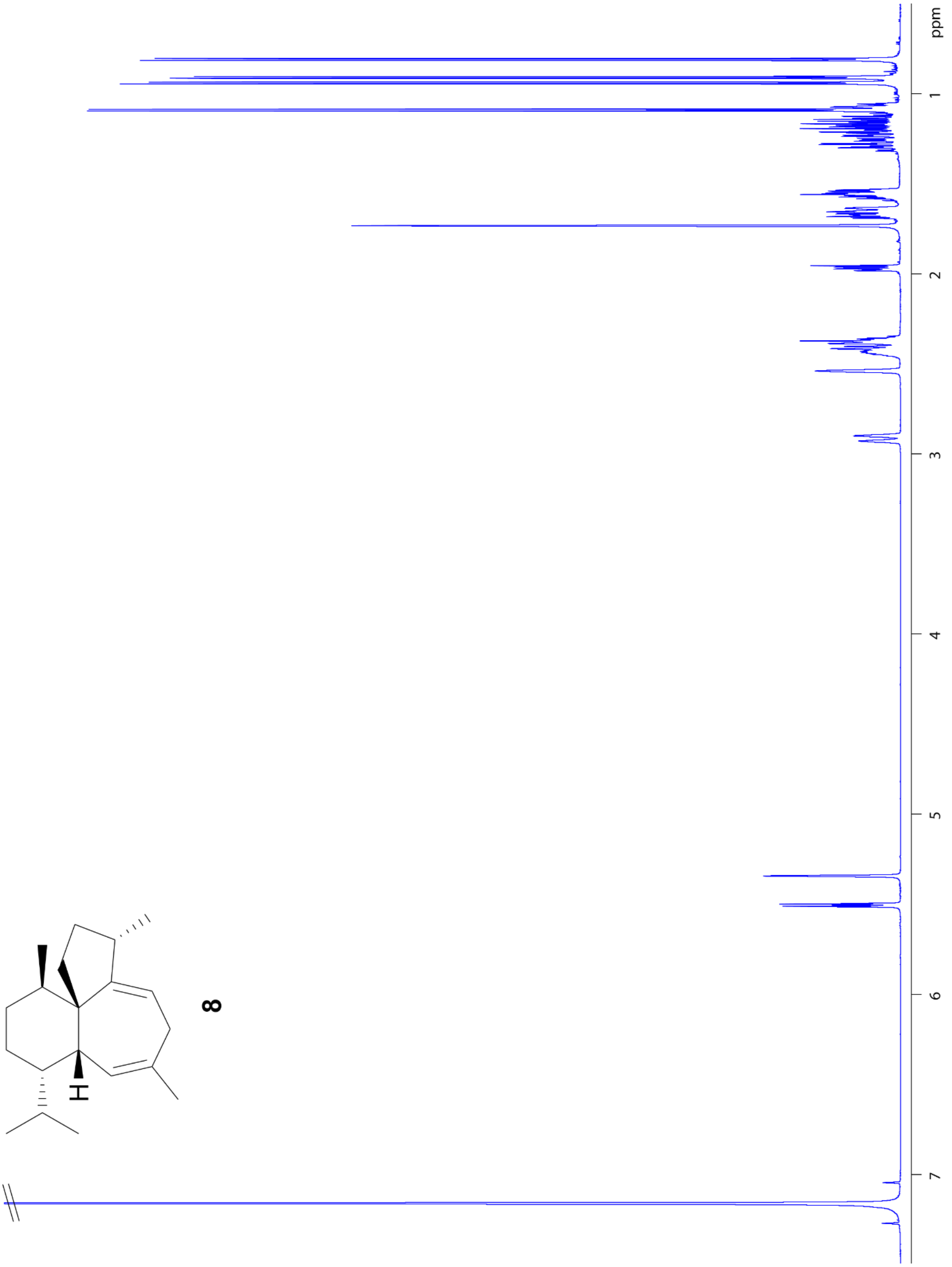

Figure S20. ^{1}H-NMR spectrum of **8** (C_6D_6, 700 MHz).

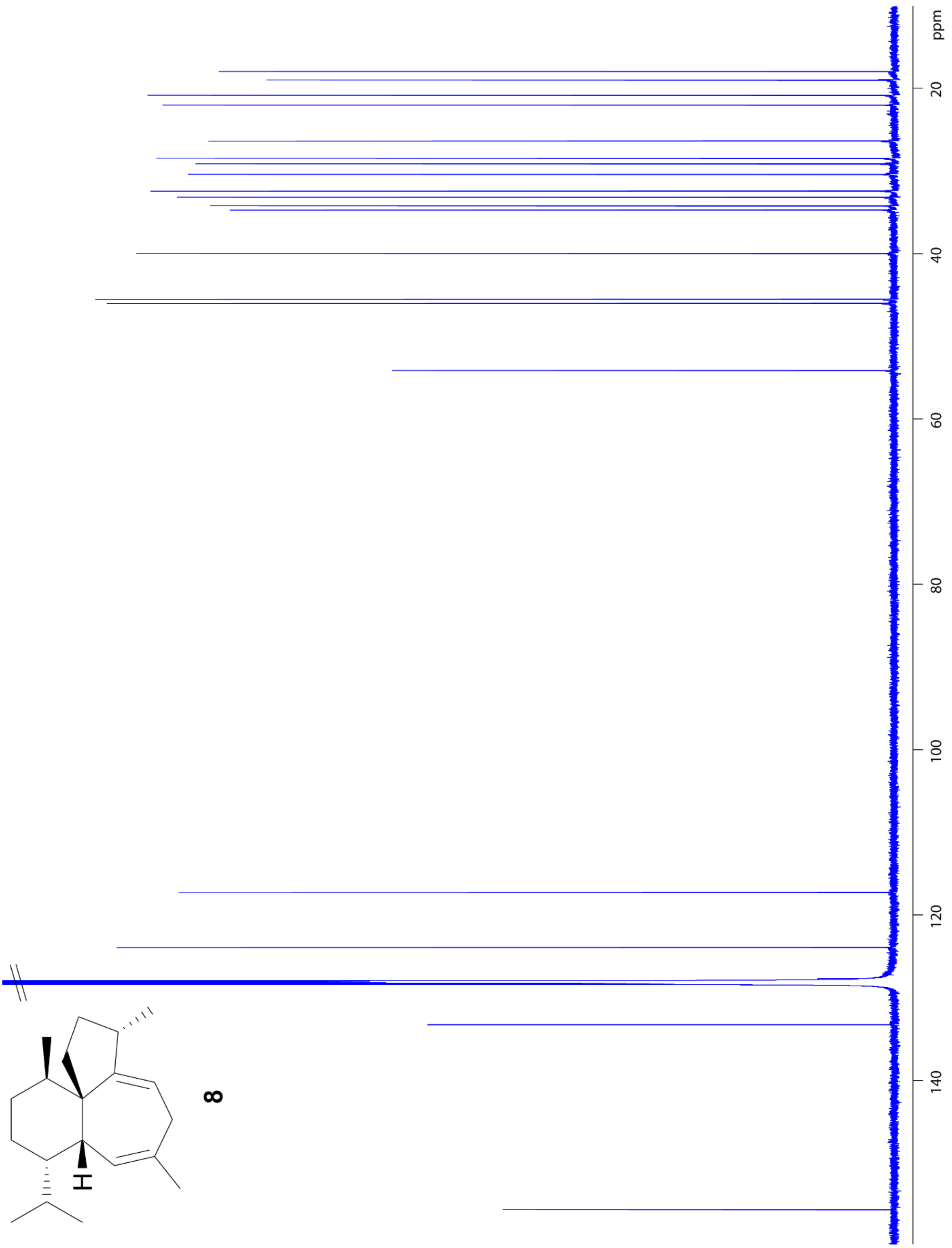

Figure S21. ^{13}C-NMR spectrum of **8** (C_6D_6, 175 MHz).

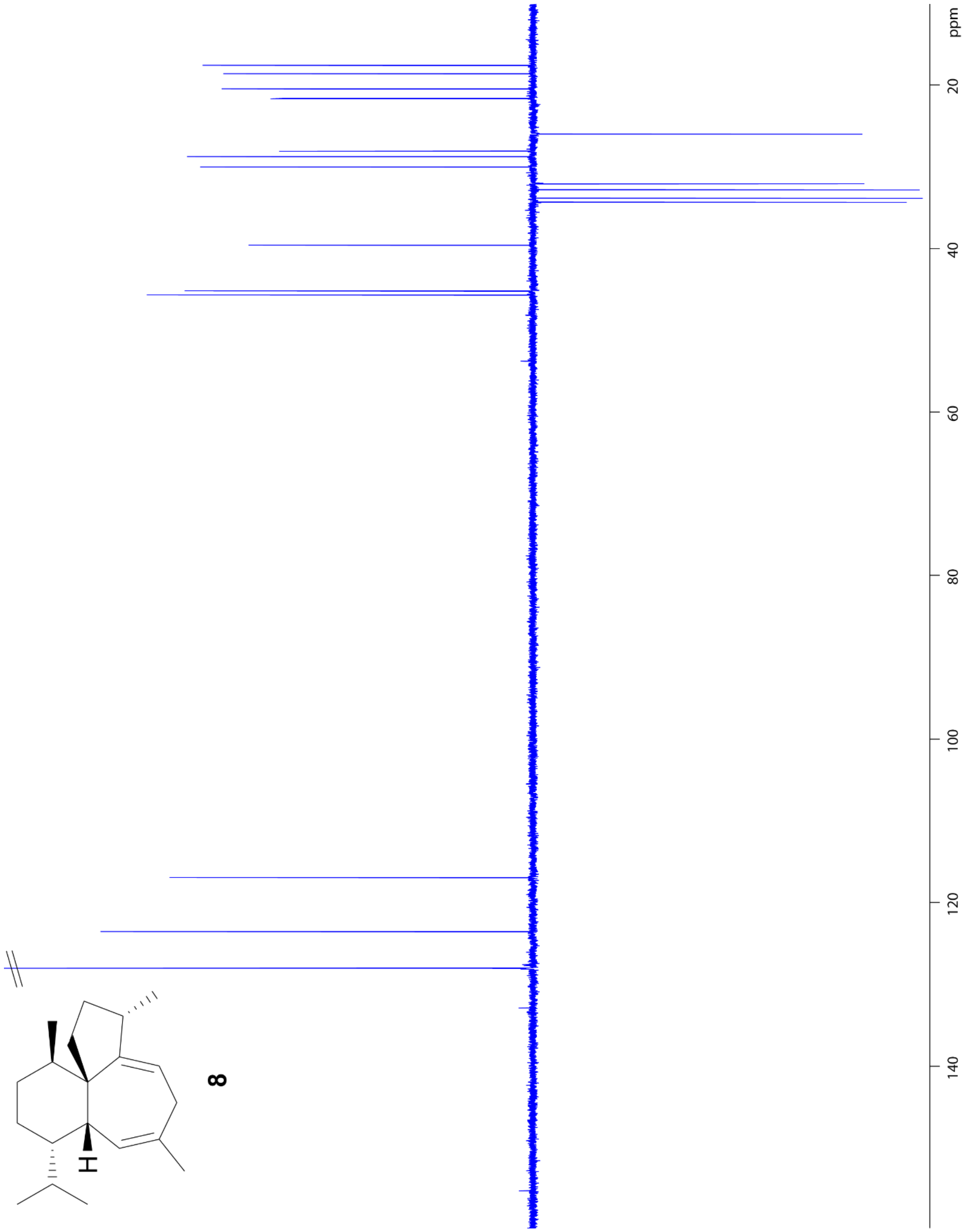

Figure S22. ^{13}C-DEPT-135 spectrum of **8** (C_6D_6, 175 MHz).

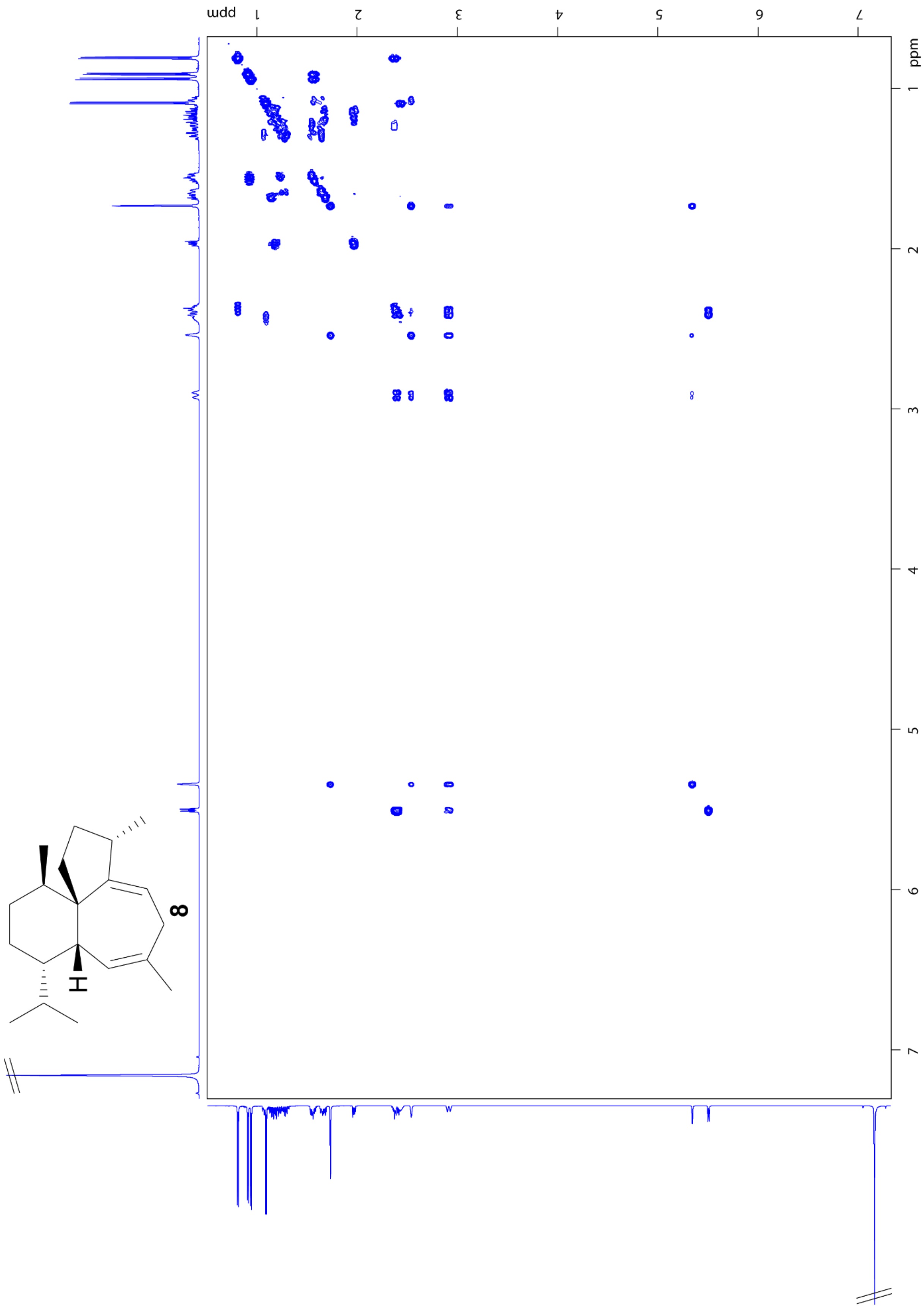

Figure S23. ^{1}H,^{1}H-COSY spectrum of **8** (C_6D_6).

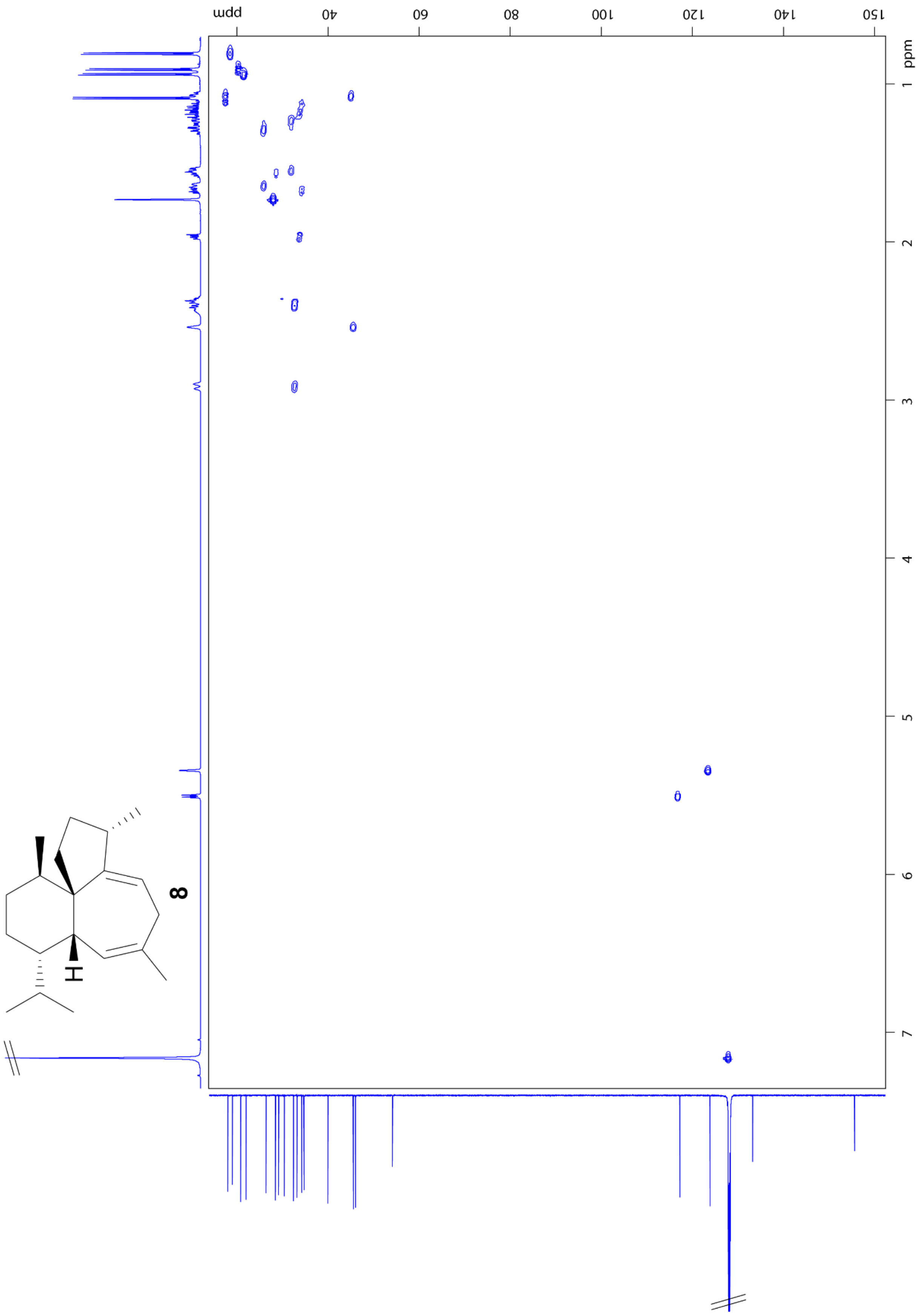

Figure S24. HSQC spectrum of **8** (C_6D_6).

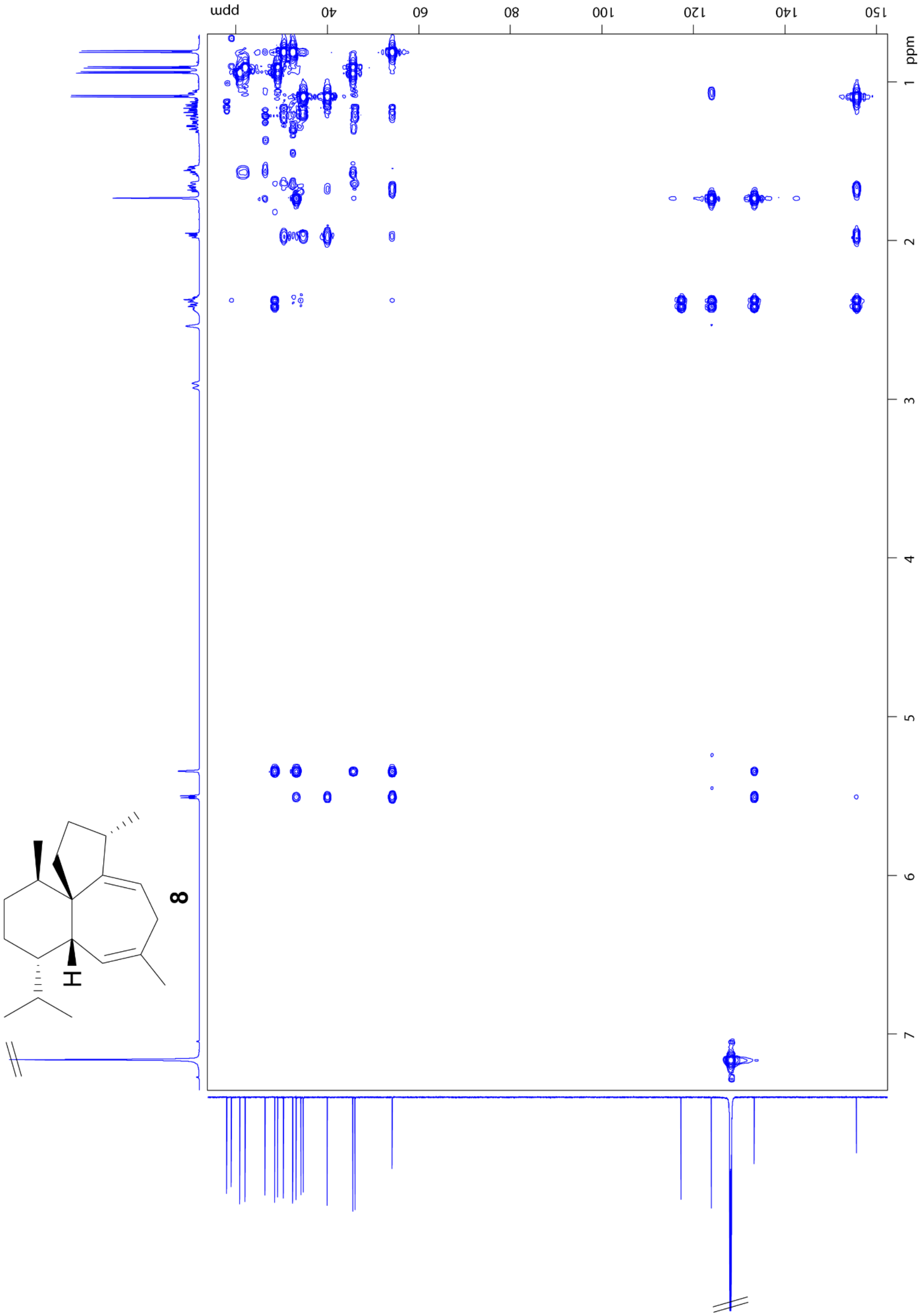

Figure S25. HMBC spectrum of **8** (C_6D_6).

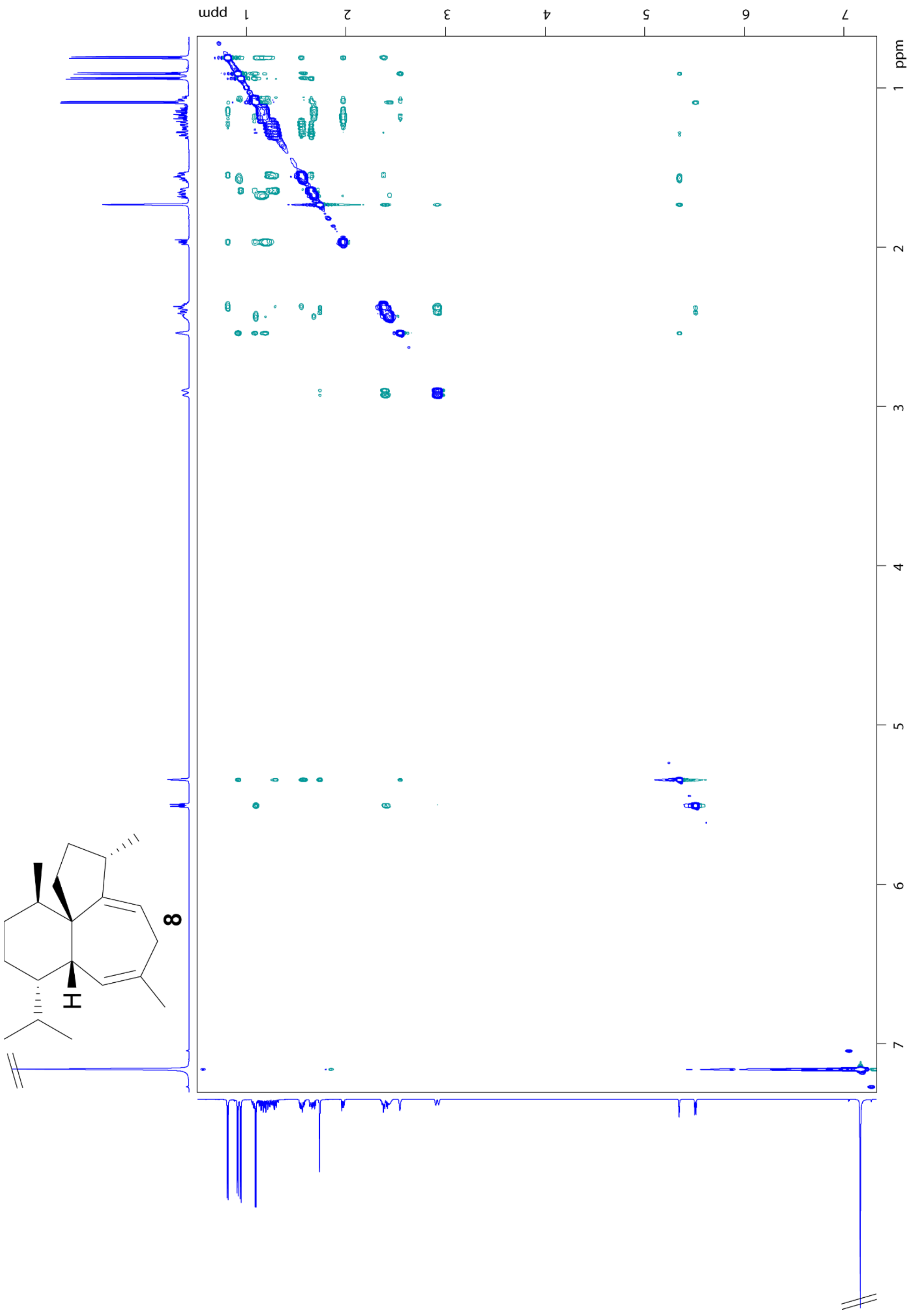

Figure S26. NOESY spectrum of **8** (C_6D_6).

Incubation experiments with isotopically labelled substrates and CwWS

Isotopic labelling experiments were performed with approximately 1 mg labelled GGPP (or its precursors) in substrate buffer (1 mL, 25 mM NH_4HCO_3), incubation buffer (4 mL), enzyme elution fractions (3 mL) with the substrates and enzyme preparations as listed in Table S5. After incubation with shaking at 28 °C overnight the products were extracted with C_6D_6 (650 μL) or *n*-hexane (200 μL) for NMR or GC/MS experiments, respectively. The extracts were dried with $MgSO_4$ and analysed by NMR and/or GC/MS.

Table S5. Isotopic labelling experiments with CwWS.

entry	substrate(s)	additional enzyme(s)	results shown in
1	DMAPP + (*E*)-(4-^{13}C,4-^{2}H)IPP[9]	FPPS[10] + GGPPS[11]	Figure S27
2	DMAPP + (*Z*)-(4-^{13}C,4-^{2}H)IPP[9]	FPPS[10] + GGPPS[11]	Figure S27
3	(*R*)-(1-^{13}C,1-^{2}H)IPP[12]	FPPS[10] + GGPPS[11] + IDI[13]	Figures S28 and S35
4	(*S*)-(1-^{13}C,1-^{2}H)IPP[12]	FPPS[10] + GGPPS[11] + IDI[13]	Figures S28 and S35
5	FPP + (1-^{13}C)IPP[11]	GGPPS[11]	Figure S29
6	FPP + (2-^{13}C)IPP[14]	GGPPS[11]	Figure S29
7	FPP + (3-^{13}C)IPP[11]	GGPPS[11]	Figure S29
8	FPP + (4-^{13}C)IPP[11]	GGPPS[11]	Figure S29
9	(1-^{13}C)FPP[15] + IPP	GGPPS[11]	Figure S29
10	(2-^{13}C)FPP[15] + IPP	GGPPS[11]	Figure S29
11	(3-^{13}C)FPP[15] + IPP	GGPPS[11]	Figure S29
12	(4-^{13}C)FPP[15] + IPP	GGPPS[11]	Figure S29
13	(5-^{13}C)FPP[15] + IPP	GGPPS[11]	Figure S29
14	(6-^{13}C)FPP[15] + IPP	GGPPS[11]	Figure S29
15	(7-^{13}C)FPP[15] + IPP	GGPPS[11]	Figure S29
16	(8-^{13}C)FPP[15] + IPP	GGPPS[11]	Figure S29
17	(9-^{13}C)FPP[15] + IPP	GGPPS[11]	Figure S29
18	(10-^{13}C)FPP[15] + IPP	GGPPS[11]	Figure S29
19	(11-^{13}C)FPP[15] + IPP	GGPPS[11]	Figure S29
20	(12-^{13}C)FPP[15] + IPP	GGPPS[11]	Figure S29
20	(9-^{13}C)GPP[16] + IPP	GGPPS[11]	Figure S29
21	(10-^{13}C)GPP[17] + IPP	GGPPS[11]	Figure S29
22	(15-^{13}C)FPP[15]	GGPPS[11]	Figure S29
23	(20-^{13}C)GGPP[11]	–	Figure S29
24	(11-^{13}C)FPP[15] + (*R*)(1-^{2}H)IPP[14]	GGPPS[11]	Figures S30 and S31
25	(11-^{13}C)FPP[15] + (*S*)(1-^{2}H)IPP[14]	GGPPS[11]	Figures S30 and S31
26	(*R*)(1-^{2}H)GGPP[11]	–*	Figure S32
27	(*S*)(1-^{2}H)GGPP[11]	–*	Figure S32
28	(3-^{13}C,2-^{2}H)GPP[13] + IPP	GGPPS[11]	Figure S33
29	(3-^{13}C,2-^{2}H)FPP[22] + IPP	GGPPS[11]	Figure S34

*Entries 26 and 27 were performed with BdS[9] instead of CwWS.

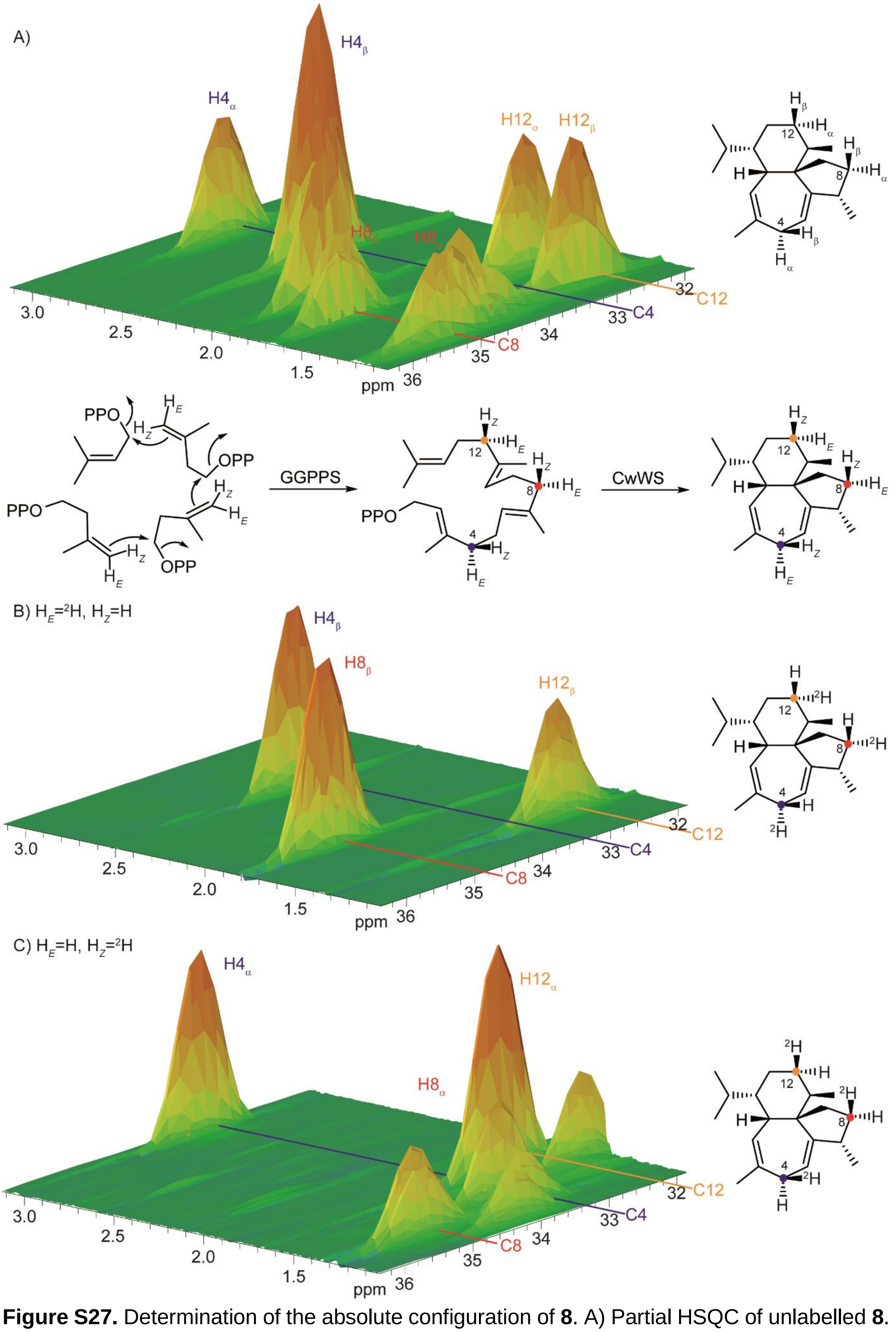

Figure S27. Determination of the absolute configuration of **8**. A) Partial HSQC of unlabelled **8**. Incubation of CwWS with DMAPP, GGPPS and B) (*E*)(4-^{2}H,4-^{13}C)IPP and C) (*Z*)(4-^{2}H,4-^{13}C)IPP. Coloured dots indicate ^{13}C labelling.

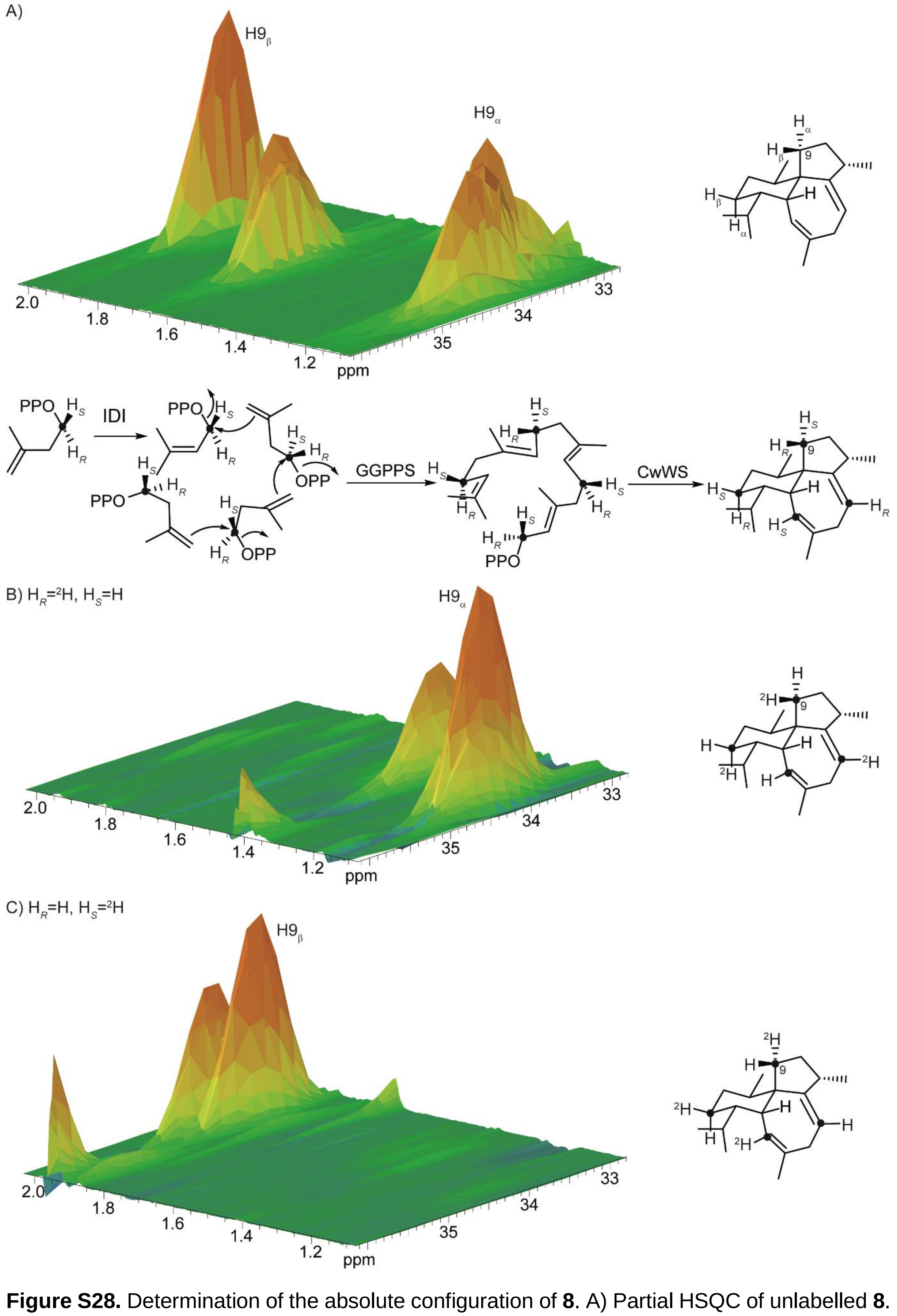

Figure S28. Determination of the absolute configuration of **8**. A) Partial HSQC of unlabelled **8**. Incubation of CwWS with DMAPP, IDI, GGPPS and B) (*R*)(1-^{2}H,1-^{13}C)IPP and C) (*S*)(1-^{2}H,1-^{13}C)IPP. Black dots indicate ^{13}C labelling.

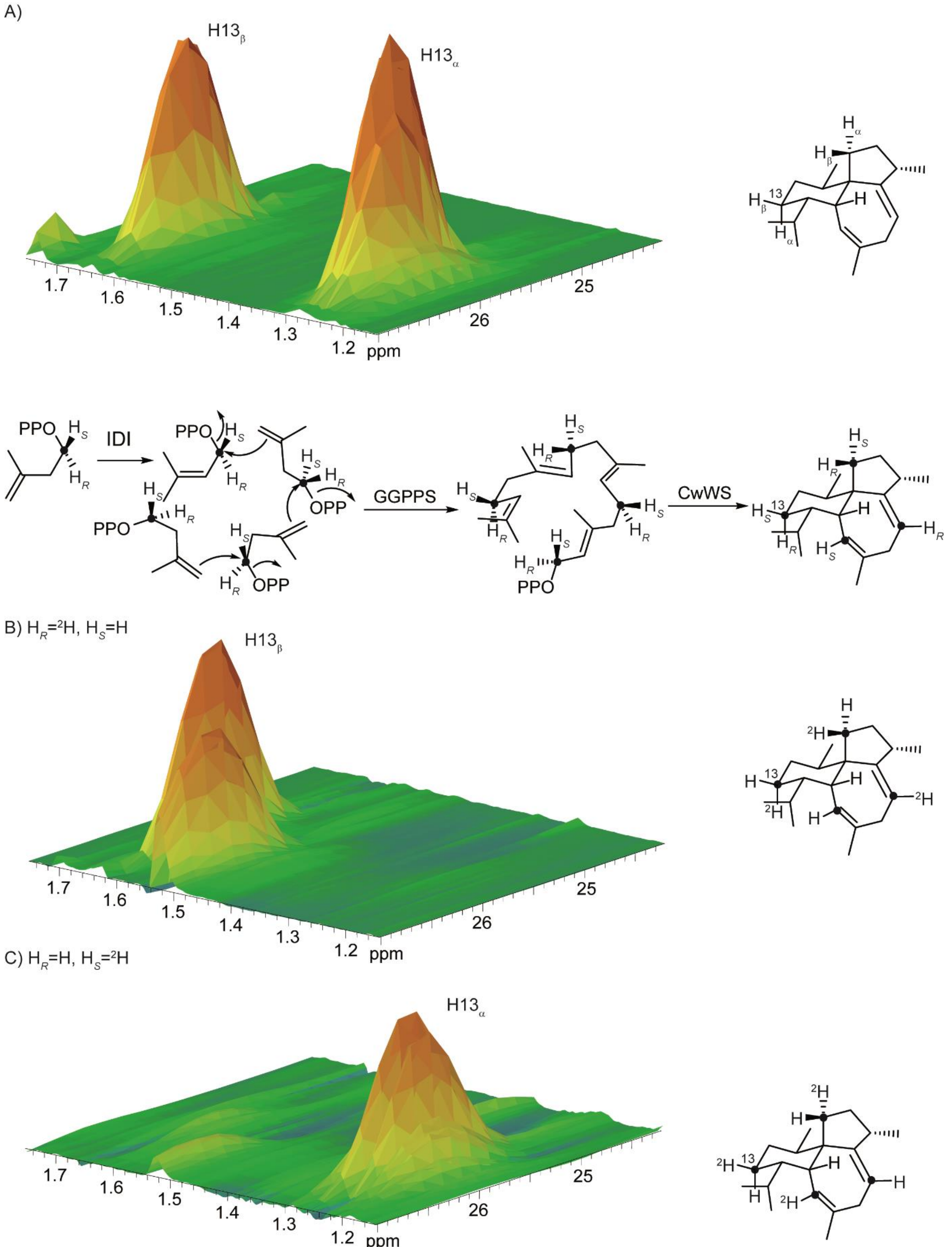

Figure S28 (continued). Determination of the absolute configuration of **8**. A) Partial HSQC of unlabelled **8**. Incubation of CwWS with DMAPP, IDI, GGPPS and B) (*R*)(1-^{2}H,1-^{13}C)IPP and C) (*S*)(1-^{2}H,1-^{13}C)IPP. Black dots indicate ^{13}C labelling.

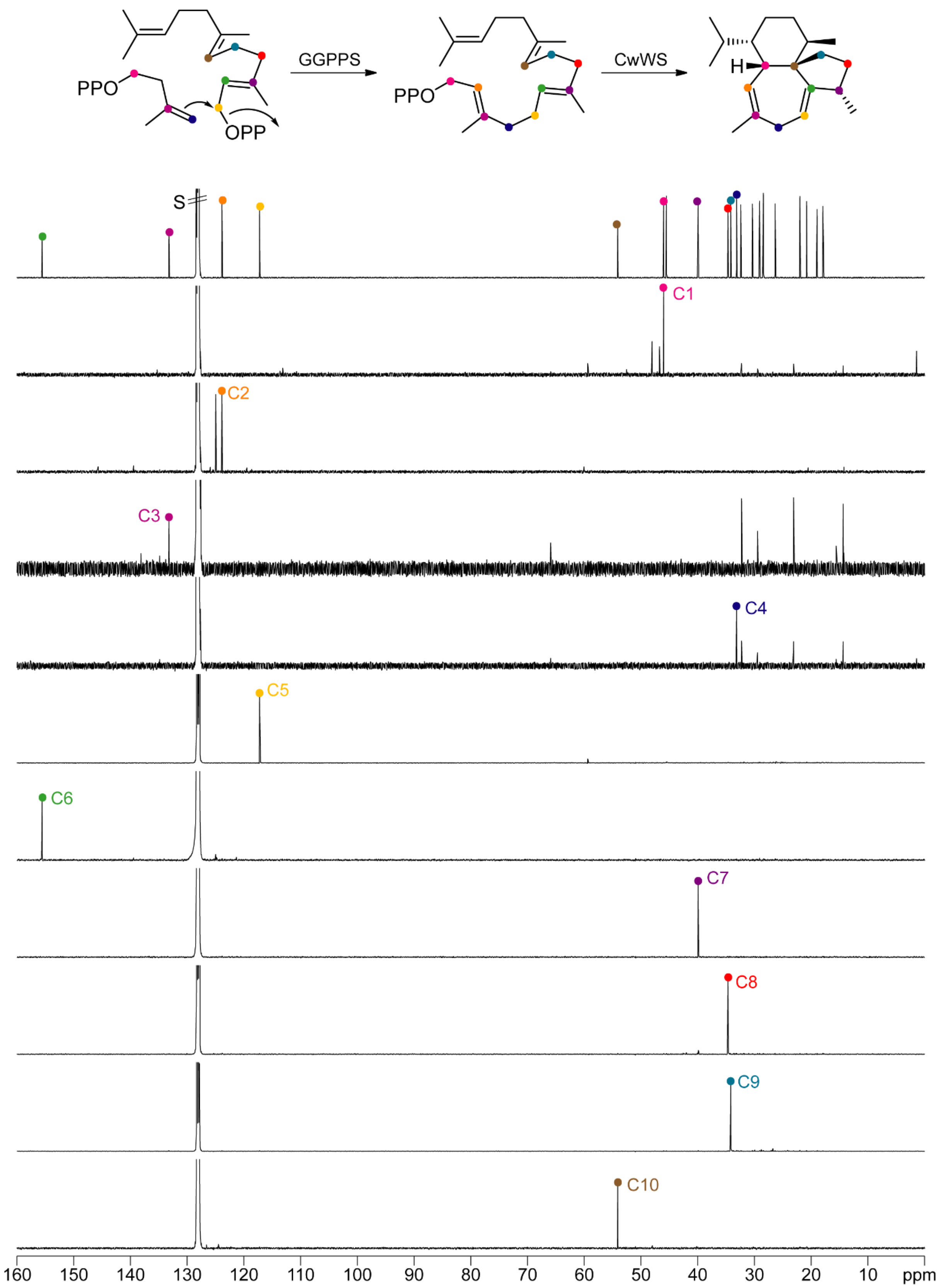

Figure S29. ^{13}C-NMR spectra of **8** obtained from incubation of CwWS with either (^{13}C)GGPP isotopomers or isotopomers prepared in situ from (^{13}C)IPP and FPP or IPP and (^{13}C)FPP with GGPPS.

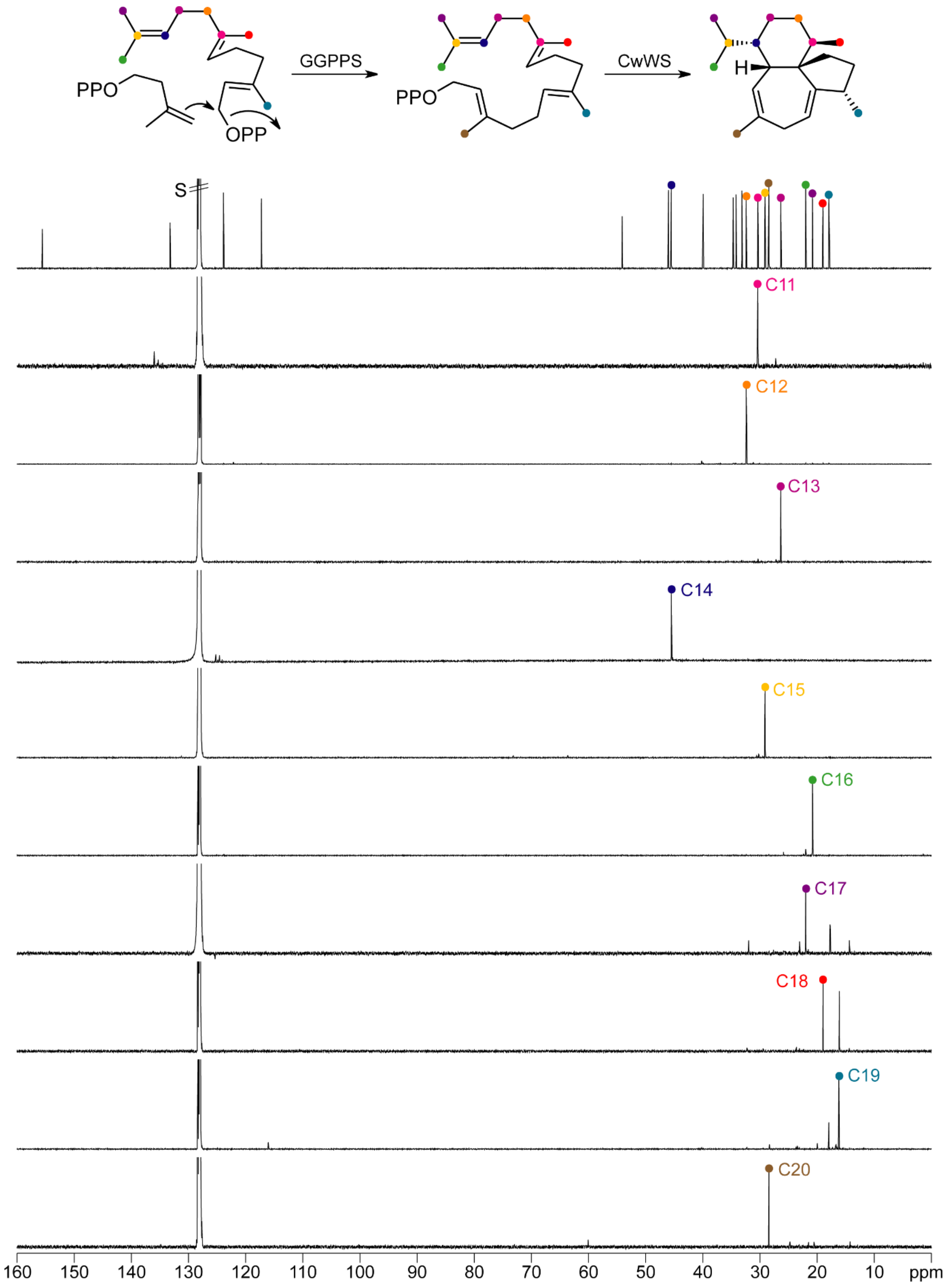

Figure S29 (continued). ^{13}C-NMR spectra of **8** obtained from incubation of CwWS with either (^{13}C)GGPP isotopomers or isotopomers prepared in situ from (^{13}C)IPP and FPP or IPP and (^{13}C)FPP with GGPPS.

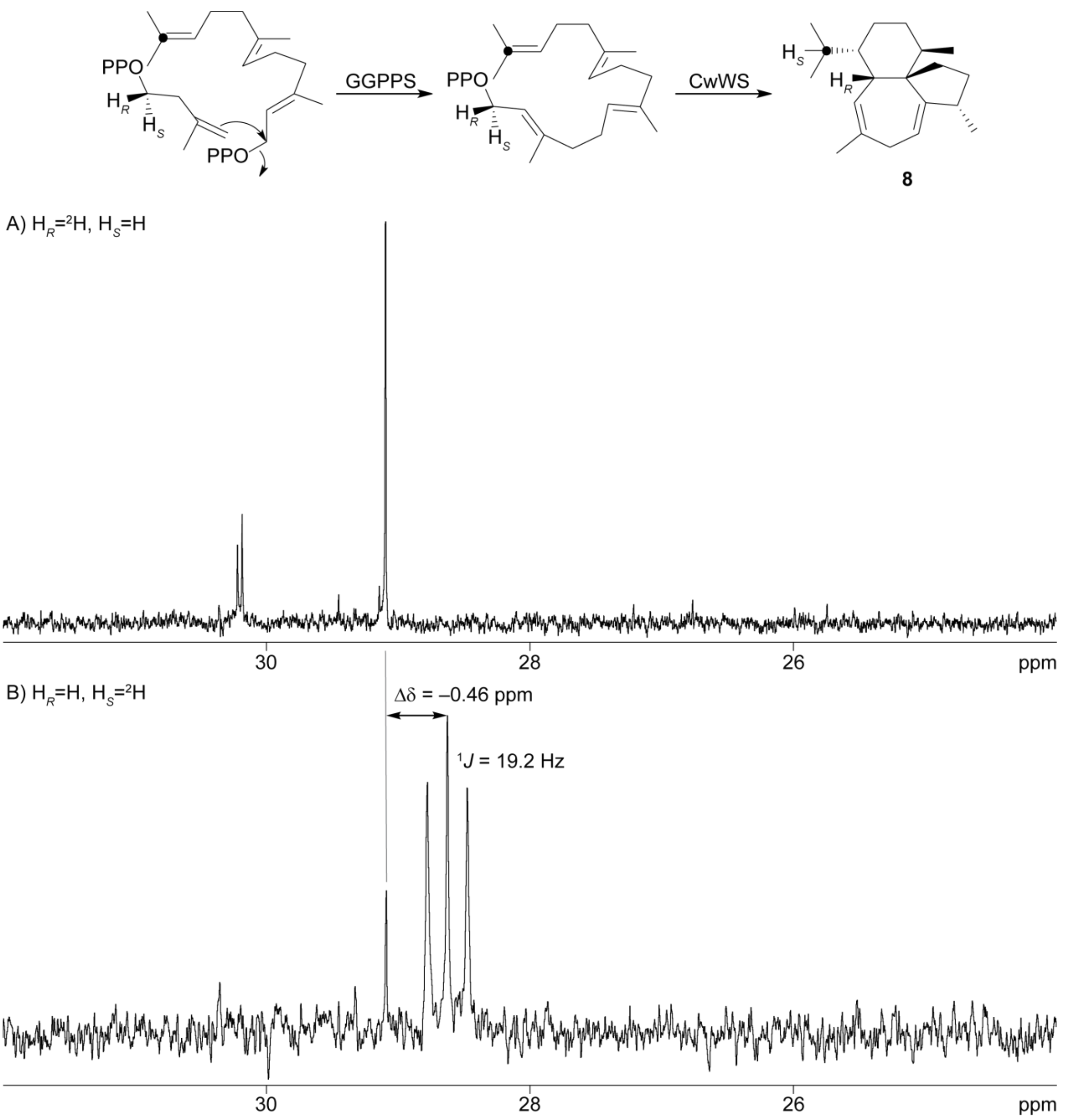

Figure S30. Investigation of the the 1,3-hydride shift from C1 to C15 in the cyclisation of GGPP using CwWS. Partial ^{13}C-NMR spectra obtained by incubation of CwWS with (11-^{13}C)FPP, GGPPS and A) (*R*)(1-^{2}H)IPP and B) (*S*)(1-^{2}H)IPP.

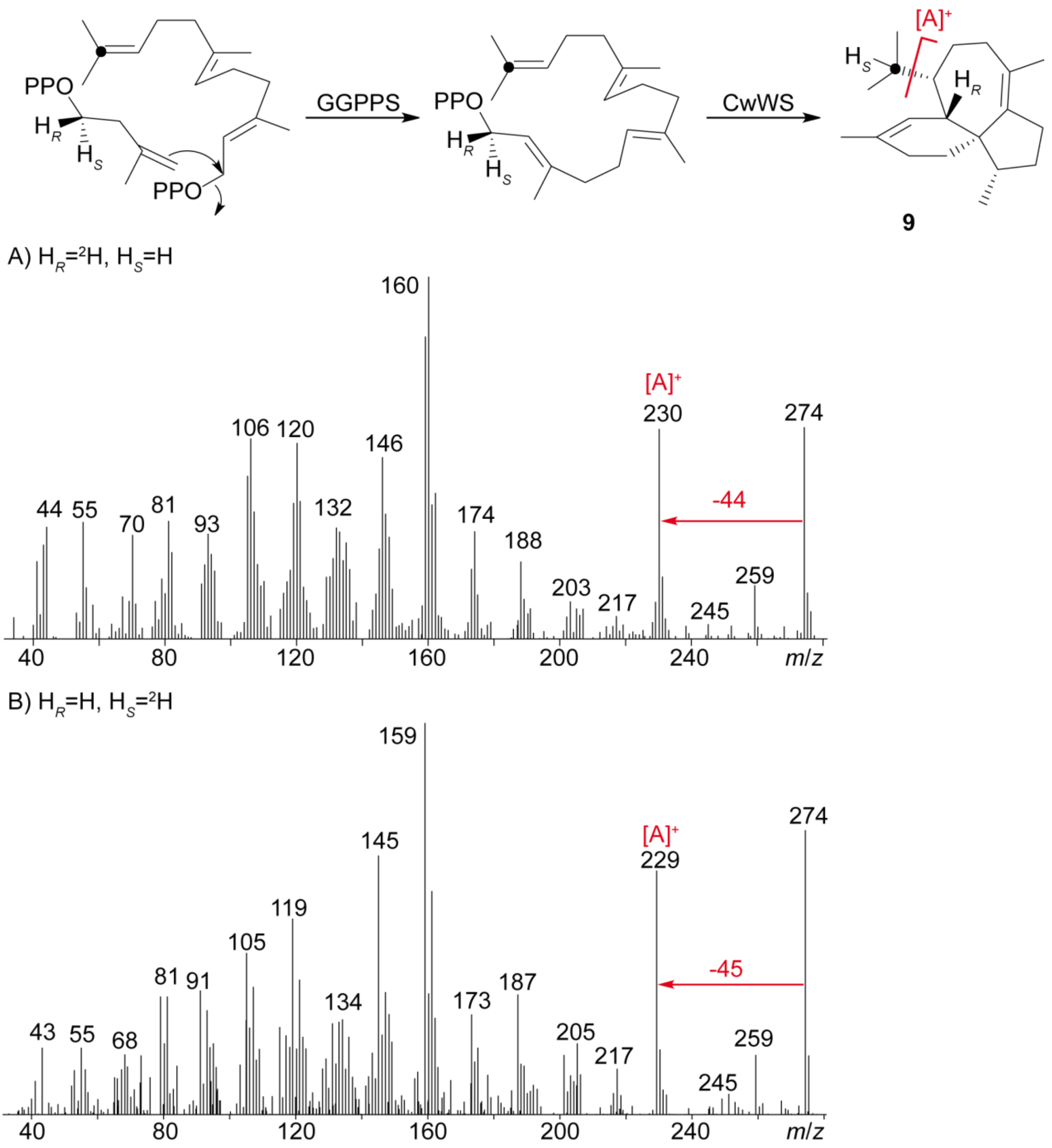

Figure S31. Investigation of the the 1,3-hydride shift from C1 to C15 in the cyclisation of GGPP using CwWS. A) EI mass spectrum of the side product **9** obtained from an incubation of (11-^{13}C)FPP and (*R*)-(1-^{13}C,1-^{2}H)IPP with GGPPS and CwWS. The fragment ion $[A]^+$ arises by cleavage of a single labelled *i*Pr group. B) EI mass spectrum of the side product **9** obtained from an incubation of (11-^{13}C)FPP and (*S*)-(1-^{13}C,1-^{2}H)IPP with GGPPS and CwWS. The fragment ion $[A]^+$ arises by cleavage of a deuterium containing double labelled *i*Pr group, which supports the proposed 1,3-hydride shift. This hydride shift proceeds with the highly stereoselective migration of the 1-*pro*-*S* hydrogen. This contrary finding to the biosynthesis of bonnadiene in *Allokutzneria albata* indicates that CwWS produces **9** as *ent*-bonnadiene.

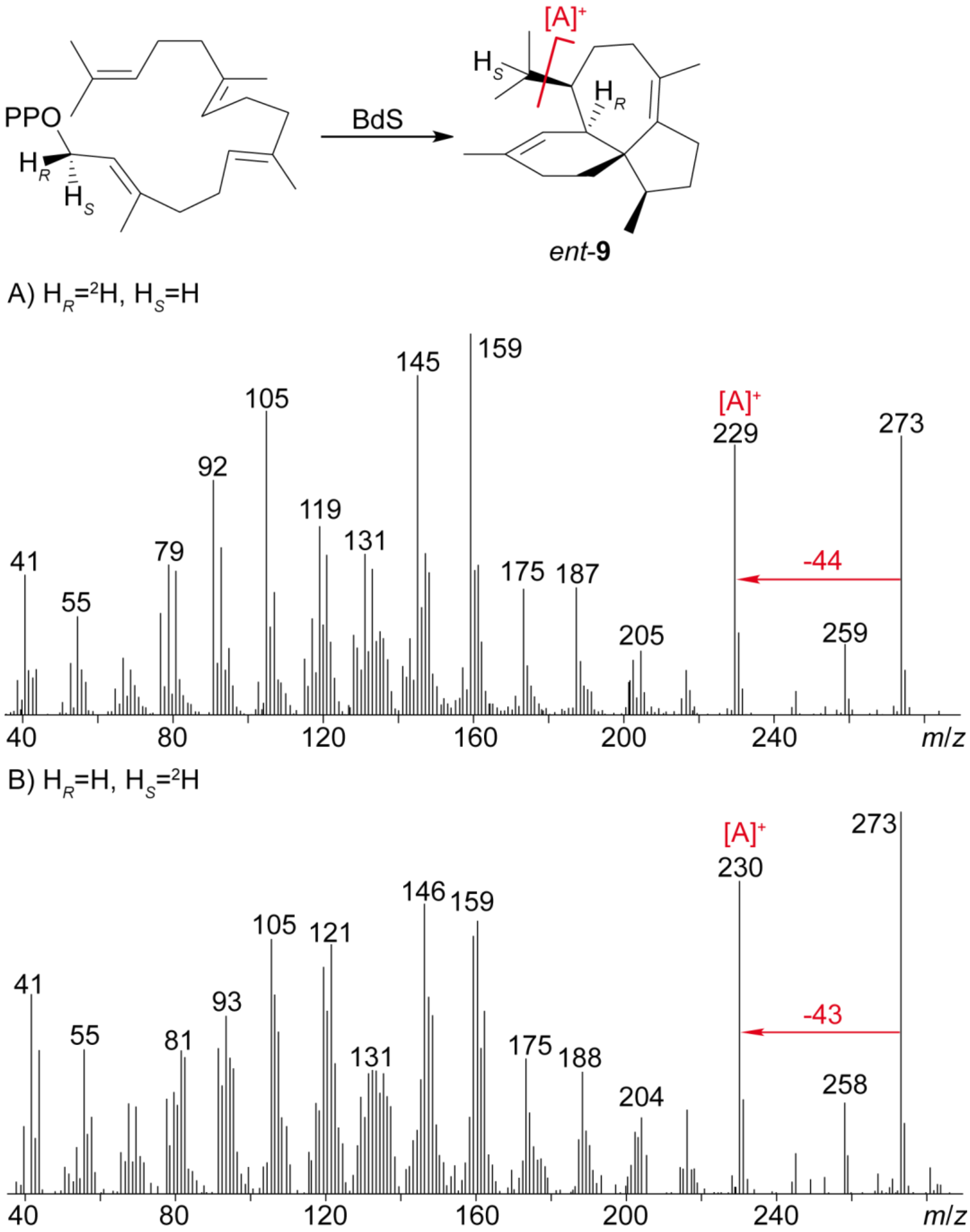

Figure S32. Investigation of the the 1,3-hydride shift from C1 to C15 in the cyclisation of GGPP using BdS. A) EI mass spectrum of *ent*-**9** obtained from an incubation of (*R*)-(1-^{13}C,1-^{2}H)GGPP with BdS. The fragment ion [A]$^{+}$ arises by cleavage of a deuterium labelled *i*Pr group. B) EI mass spectrum of *ent*-**9** obtained from an incubation of (*S*)-(1-^{13}C,1-^{2}H)GGPP with BdS. The fragment ion [A]$^{+}$ arises by cleavage of a non- labelled *i*Pr group. This hydride shift proceeds with the highly stereoselective migration of the 1-*pro*-*R* hydrogen. This finding clearly indicates that bonnadiene produced by BdS is *ent*-**9**.

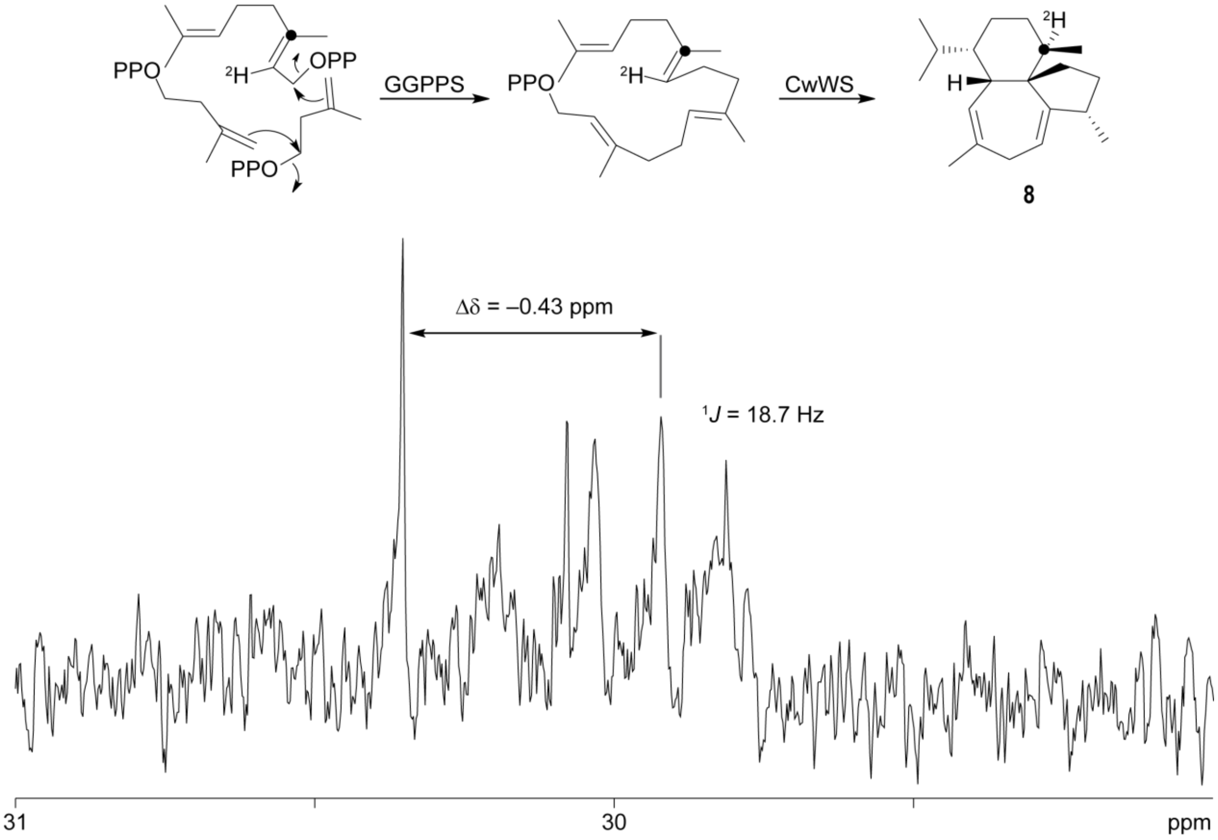

Figure S33. Investigation of the 1,2-hydride shift from cation **K** to **L** in the cyclisation of GGPP to **8**. (3-^{13}C,2-^{2}H)GPP was elongated with IPP and GGPPS and the resulting (11-^{13}C,10-^{2}H)GGPP was converted with CwWS to yield (11-^{13}C,11-^{2}H)-**8**. The upfield shifted triplet indicates direct linkage of ^{2}H and ^{13}C.

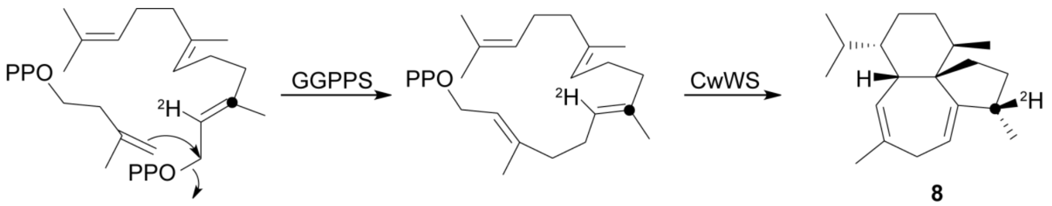

8

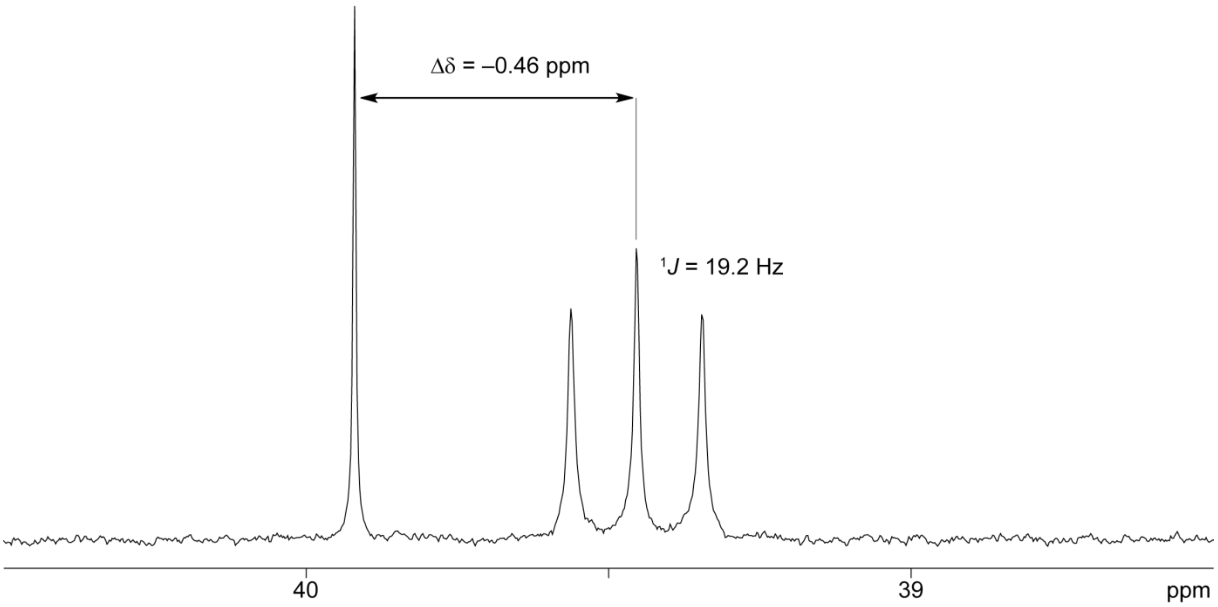

Figure S34. Investigation of the 1,2-hydride shift from cation **M** to **N** in the cyclisation of GGPP to **8**. (3-^{13}C,2-^{2}H)FPP was elongated with IPP and GGPPS and the resulting (7-^{13}C,6-^{2}H)GGPP was converted with CwWS to yield (7-^{13}C,7-^{2}H)-**8**. The upfield shifted triplet indicates direct linkage of ^{2}H and ^{13}C.

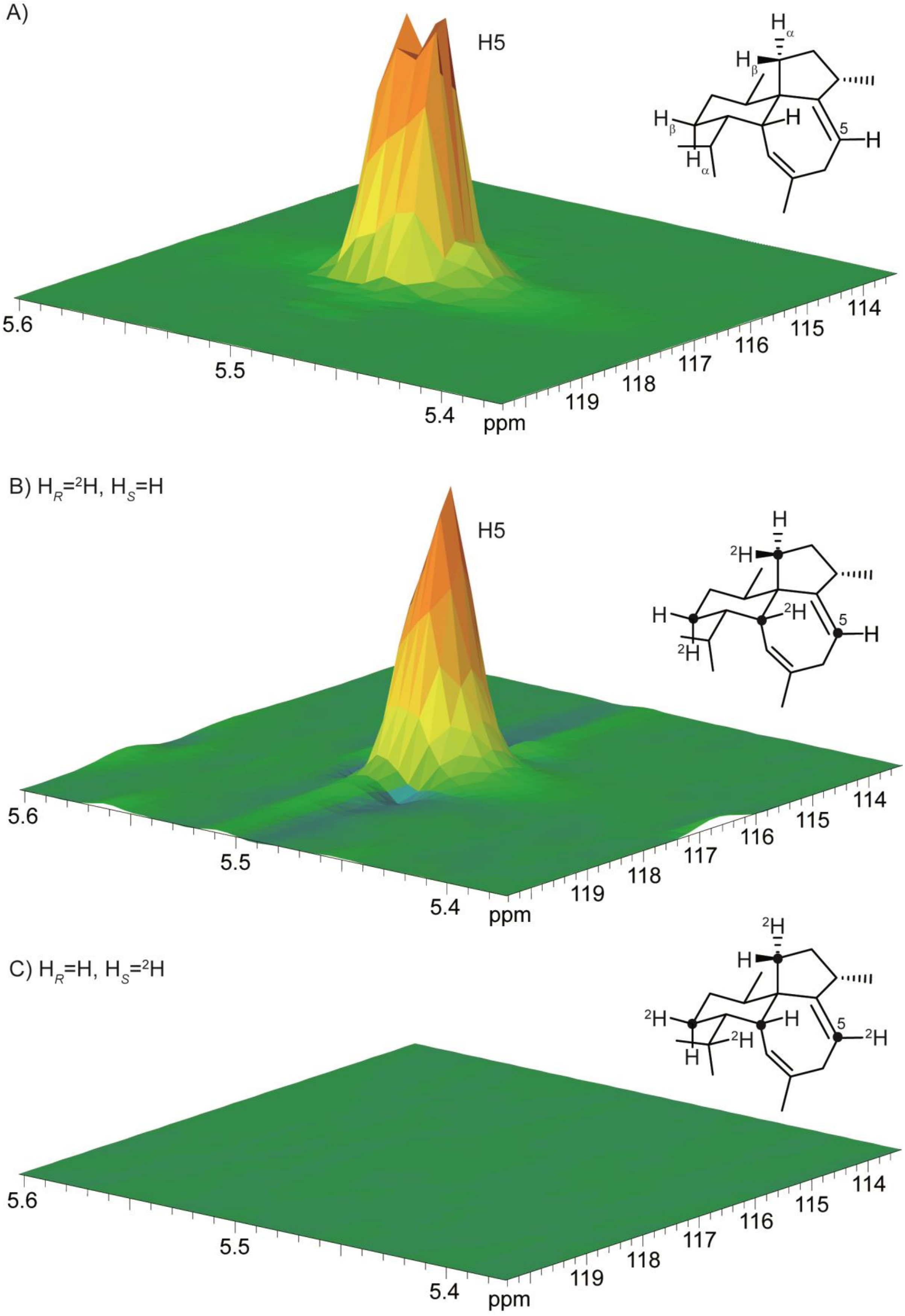

Figure S35. Partial HSQC spectra showing the region for H5 of A) unlabelled **8**, B) ($^{13}C_4$,$^{2}H_3$)-**1** obtained from (*R*)-(1-^{13}C,1-^{2}H)IPP, and C) ($^{13}C_4$,$^{2}H_4$)-**8** obtained from (*S*)-(1-^{13}C,1-^{2}H)IPP. The vanished crosspeak in C) indicates a substitution of H5 by deuterium.

CLSA analyses

For CLSA analyses 1496 PGY agar plates were inoculated with 1 mL taken from a fresh liquid culture grown for 3 days. The plates were then incubated for 7 days and subjected to the CLSA to collect the volatile compounds for 24 h. The adsorbed compounds were extracted from the activated charcoal using 10 units of dichloromethane, 10 μL each. The combined extracts were analysed by GC/MS.

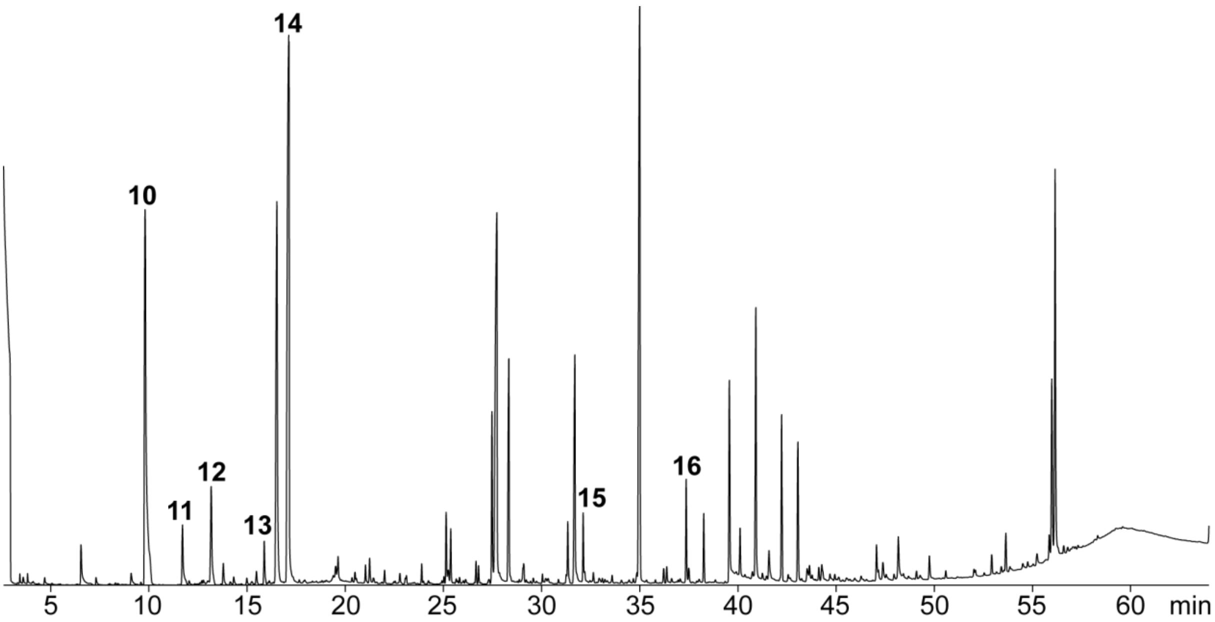

Figure S36. Total ion chromatogram of a CLSA headspace extract from *C. polytrichastri*. Numbers at peaks refer to compounds that were unambiguously identified from their mass spectra and retention indices by comparison to library spectra and published data (Table S6).

Table S6. Volatiles produced by *C. polytrichastri*.

compound	*I*	*I* (Lit.)
2,5-dimethylpyrazine (**10**)	911	912[23]
benzaldehyde (**11**)	963	952[24]
trimethylpyrazine (**12**)	1002	1002[23]
2-ethyl-3,6-dimethylpyrazine (**13**)	1080	1077[23]
phenethyl alcohol (**14**)	1116	1106[24]
1-tetradecanol (**15**)	1678	1671[24]
farnesylacetone (**16**)	1925	1927[25]

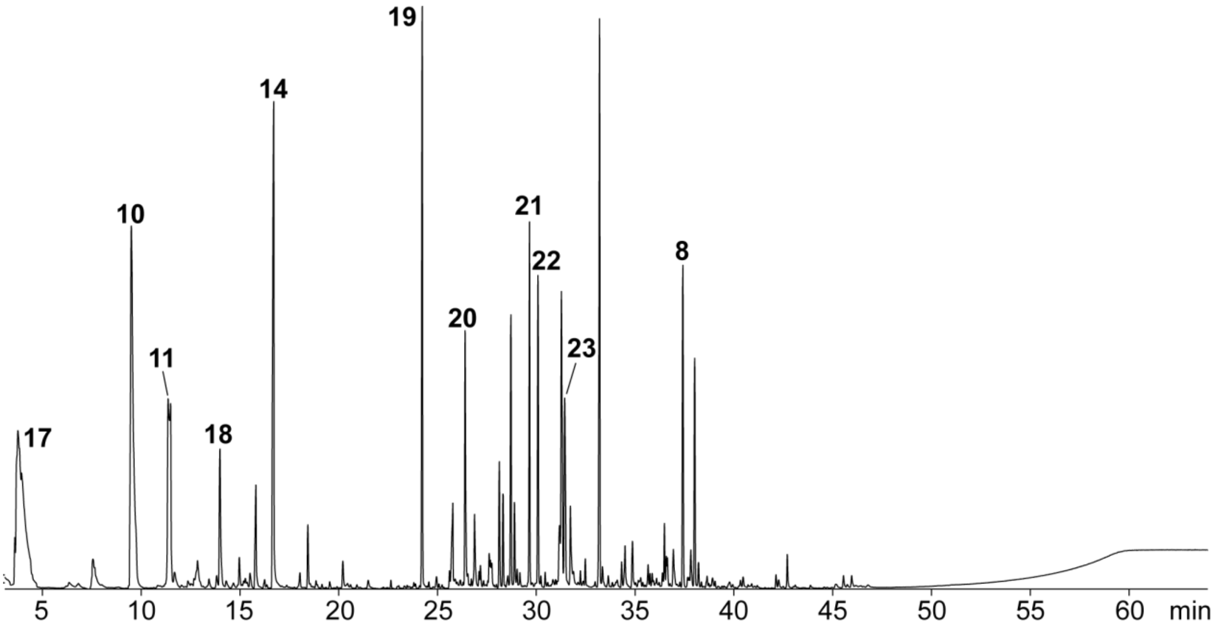

Figure S37. Total ion chrmoatogram of a CLSA headspace extract from *C. wanjuense* showing the production of wanjudiene (**8**). Numbers at peaks refer to compounds that were unambiguously identified from their mass spectra and retention indices by comparison to library spectra and published data (Table S7).

Table S7. Volatiles produced by *C. wanjuense*.

compound	*I*	*I* (Lit.)
isopentyl alcohol (**17**)	725	731[24]
2,5-dimethylpyrazine (**10**)	905	912[23]
benzaldehyde (**11**)	958	952[24]
benzyl alcohol (**18**)	1026	1026[24]
phenethyl alcohol (**14**)	1112	1106[24]
2,5-diisobutylpyrazine (**19**)	1375	1381[26]
geranylacetone (**20**)	1449	1449[24]
germacrene D-4-ol (**21**)	1581	1574[24]
viridiflorol (**22**)	1599	1592[24]
α-cadinol (**23**)	1660	1652[24]
wanjudiene (**8**)	1945	–

References

[1] G. R. Fulmer, A. J. M. Miller, N. H. Sherden, H. E. Gottlieb, A. Nudelman, B. M. Stoltz, J. E. Bercaw, K. I. Goldberg, *Organometallics* **2010**, *29*, 2176.

[2] B. Neumann, A. Pospiech, H. U. Schairer, *Trends Genet*. **1992**, *8*, 332.

[3] R. D. Giets, R. H. Schiestl, *Nat. Protoc*. **2007**, *2*, 31.

[4] J. S. Dickschat, K. A. K. Pahirulzaman, P. Rabe, T. A. Klapschinski, *ChemBioChem* **2014**, *15*, 810.

[5] C. M. Starks, K. Back, J. Chappell, J. P. Noel, *Science* **1997**, *277*, 1815.

[6] E. Y. Shishova, L. Di Costanzo, D. E. Cane, D. W. Christianson, *Biochemistry* **2007**, *46*, 1941.

[7] P. Baer, P. Rabe, K. Fischer, C. A. Citron, T. A. Klapschinski, M. Groll, J. S. Dickschat, *Angew. Chem. Int. Ed.* **2014**, *53*, 7652.

[8] J. S. Dickschat, *Nat. Prod. Rep.* **2016**, *33*, 87.

[9] L. Lauterbach, J. Rinkel, J. S. Dickschat, *Angew. Chem. Int. Ed.* **2018**, *57*, 8280.

[10] P. Rabe, J. Rinkel, B. Nubbemeyer, T. G. Köllner, F. Chen, J. S. Dickschat, *Angew. Chem. Int. Ed.* **2016**, *55*, 15420.

[11] P. Rabe, J. Rinkel, E. Dolja, T. Schmitz, B. Nubbemeyer, T. H. Luu, J. S. Dickschat, *Angew. Chem. Int. Ed*. **2017**, *56*, 2776-2779.

[12] J. Rinkel, J. S. Dickschat, *Org. Lett.* **2019**, *21*, 2426.

[13] J. Rinkel, L. Lauterbach, P. Rabe, J. S. Dickschat, *Angew. Chem. Int. Ed.* **2018**, *57*, 3238.

[14] J. Rinkel, L. Lauterbach, J. S. Dickschat, *Angew. Chem. Int. Ed.*, **2019**, *58*, 452.

[15] P. Rabe, L. Barra, J. Rinkel, R. Riclea, C. A. Citron, T. A. Klapschinski, A. Janusko, J. S. Dickschat, *Angew. Chem. Int. Ed.* **2015**, *54*, 13448.

[16] G. Bian, J. Rinkel, Z. Wang, L. Lauterbach, A. Hou, Y. Yuan, Z. Deng, T. Liu, J. S. Dickschat, *Angew. Chem. Int. Ed.* **2018**, *57*, 15887.

[17] T. Mitsuhashi, J. Rinkel, M. Okada, I. Abe, J. S. Dickschat, *Chem. Eur. J.* **2017**, *23*, 10053.

[18] S. Grimme, S. Ehrlich and L. Goerigk, *J. Comp. Chem.* **2011**, *32*, 1456.

[19] Gaussian 16, Revision B.01, M. J. Frisch, et al., Gaussian, Inc., Wallingford CT, **2016**.

[20] a) Y. J. Hong and D. J. Tantillo, *J. Org. Chem.* **2018**, *83*, 3780; b) C. Adamo, V. Barone, *J. Chem. Phys.* **1998**, *108*, 664; c) S. P. T. Matsuda, W. K. Wilson, Q. Xiong, *Org. Biomol. Chem.* **2006**, *4*, 530.

[21] a) S. Grimme, *Chem. Eur. J.* **2012**, *18*, 9955, b) GoodVibes v3.0.1, G. Luchini, J. V. Alegre-Requena, Y. Guan,I. Funes-Ardoiz, R. S. Paton, **2019**.

[22] T. A. Klapschinski, P. Rabe, J. S. Dickschat, *Angew. Chem. Int. Ed.* **2016**, *55*, 10141.

[23] J. S. Dickschat, S. Wickel, C. J. Bolten, T. Nawrath, S. Schulz, C. Wittmann, *Eur. J. Org. Chem.* **2010**, 2687.

[24] R. P. Adams, Identification of Essential Oil Components by Gas Chromatography/Mass Spectrometry, Allured, Carol Stream, **2009**.

[25] R. Palic, G. Stojanovic, S. Alagic, M. Nikolic, Z. Lepojevic, *Flavour Fragr. J.* **2002**, *17*, 323.

[26] J. S. Dickschat, H. Reichenbach, I. Wagner-Döbler, S. Schulz, *Eur. J. Org. Chem.* **2005**, 4141.

Structure 1 = A
B97D3/6-31G** = 781.2740532 H, NIMAG =0
Gibbs-correction (298.15 K, entropic quasi-harmonic correction)= 0.0014930 H
mPW1PW91/6-311+G(d,p)// B97D3/6-31G** = 781.7175094 H

C	-1.38347000	-1.73919600	-0.53815100
C	-0.96177900	-0.21648200	-0.56937600
C	-0.58729200	0.45599600	-1.90325400
C	0.13572100	1.79952000	-1.69239900
C	1.47069300	1.75333800	-0.97135300
C	2.15703900	0.58563900	-0.88851100
C	1.05448100	-2.28195900	0.09457600
C	-0.39673400	-2.52670900	0.40390700
C	-2.79007800	-1.72390800	0.11290300
C	-2.81255600	-0.49806200	1.03610200
C	-2.11809500	0.58192000	0.11425100
C	3.38952000	0.22313900	-0.11187100
C	3.33272900	-1.29192200	0.26863900
C	1.98389100	-1.66447600	0.86102800
C	-1.67251700	1.75233900	0.87336000
C	-2.46612800	3.00273700	0.86538200
C	-0.48052600	1.66758200	1.72926100
C	-1.43338800	-2.40263000	-1.92261600
C	1.77635100	-1.22237700	2.28790600
C	1.99633400	3.07396500	-0.45658900
H	-0.07355000	-0.14956600	0.06319300
H	-0.53546300	2.46589000	-1.10393300
H	-1.48466000	0.62367500	-2.51588200
H	0.04473400	-0.22183200	-2.49025400
H	0.23862100	2.33460700	-2.65091500
H	1.73660100	-0.26125700	-1.42761500
H	1.36871000	-2.58542400	-0.90820200
H	-0.63699000	-3.59710700	0.30684900
H	-0.61422600	-2.25688100	1.44596000
H	-3.56456000	-1.60926600	-0.65898500
H	-3.00884900	-2.64780500	0.66481800
H	-2.21114700	-0.66514500	1.93835500
H	-3.81546400	-0.17927300	1.34139200
H	-2.86609300	0.88448900	-0.63166400
H	3.48569400	0.84592500	0.78876100
H	4.30032000	0.40417300	-0.70566200
H	4.15061100	-1.51540700	0.96815300
H	3.51208500	-1.88298900	-0.63987400
H	-3.08250000	3.11981600	-0.03135100
H	-3.15662000	2.93113600	1.73034300
H	-1.85358100	3.89376700	1.04845900
H	-0.41408800	0.70037900	2.24271100
H	0.41269000	1.66115900	1.05982100
H	-0.38701500	2.50051200	2.43176100
H	-2.08593200	-1.85049500	-2.61109300
H	-0.44077000	-2.47608400	-2.38337200
H	-1.82989200	-3.42228700	-1.83233900
H	2.48285800	-1.74576500	2.94924600
H	0.76753200	-1.42163200	2.66617000
H	1.98885900	-0.14866100	2.40664100
H	2.34064900	3.69052400	-1.30093000
H	2.84614300	2.94913900	0.22346000
H	1.22010500	3.66096600	0.05681600

Structure 2 = TS-A_to_B
B97D3/6-31G** = 781.2692732 H, NIMAG=1, -779 cm-1
Gibbs-correction (298.15 K, entropic quasi-harmonic correction)= 0.0018130 H
mPW1PW91/6-311+G(d,p)// B97D3/6-31G** = 781.7130622 H

C	-1.12712500	-1.96651600	-0.45765700
C	-0.91948600	-0.41779600	-0.51722100
C	-0.62380800	0.34664200	-1.82873600
C	-0.13624300	1.73246700	-1.44044400
C	1.21180100	1.91094000	-0.87691700
C	2.07927400	0.85716200	-0.90164400
C	1.40800300	-2.14659200	0.03163700
C	0.02580400	-2.61079400	0.40003400
C	-2.46803900	-2.11093400	0.33099300
C	-2.60572500	-0.81494900	1.15584900
C	-2.16706000	0.24304900	0.11178800
C	3.39543800	0.66656500	-0.21911800
C	3.53610100	-0.84691300	0.14785700
C	2.25163500	-1.38135200	0.76640100
C	-1.83803100	1.64379500	0.56421900
C	-2.87646400	2.71141600	0.31823500
C	-1.00288400	1.80463800	1.81123400
C	-1.21111300	-2.63254200	-1.83758000
C	1.99789500	-0.92905500	2.18260000
C	1.55171100	3.26791500	-0.30518800
H	-0.07015200	-0.20504900	0.14115500
H	-0.90980900	1.95385500	-0.41179400
H	-1.51770000	0.41364500	-2.45999900
H	0.14188800	-0.16676300	-2.42288200
H	-0.52459900	2.58245200	-2.01790800
H	1.76727600	-0.02024100	-1.46206600
H	1.74143200	-2.42569800	-0.97273200
H	-0.05017200	-3.70335800	0.28729400
H	-0.17353200	-2.39766300	1.45793900
H	-3.30965700	-2.18022100	-0.37340700
H	-2.49033500	-3.01468600	0.95265300
H	-1.91590700	-0.82006600	2.01049800
H	-3.61933300	-0.63831000	1.53497600
H	-2.96084100	0.29808200	-0.64903700
H	3.47094700	1.29907200	0.67561900
H	4.23085300	0.96102200	-0.87563300
H	4.38913600	-0.96929700	0.82992700
H	3.76796900	-1.40836500	-0.76728600
H	-3.28746400	2.66041500	-0.69678700
H	-3.71080600	2.52658500	1.01597400
H	-2.49768000	3.72054900	0.51549600
H	-1.63282200	1.53202500	2.67264700
H	-0.12798500	1.14623000	1.82270000
H	-0.68068100	2.84047500	1.95639600
H	-1.98169200	-2.16205800	-2.46312300
H	-0.25676200	-2.57917800	-2.37826100
H	-1.47138600	-3.69367200	-1.73235500
H	2.76015200	-1.34870600	2.85613000
H	1.01708700	-1.22523400	2.56797000
H	2.08510600	0.16529800	2.27042600
H	1.82710000	3.96135700	-1.11329700
H	2.39932600	3.21924300	0.38627600
H	0.70093600	3.71538200	0.22442300

Structure 3 = B-conformer_1
B97D3/6-31G** = 781.2731450 H, NIMAG=0
Gibbs-correction (298.15 K, entropic quasi-harmonic correction)= 0.0028720 H
mPW1PW91/6-311+G(d,p)// B97D3/6-31G** = 781.7174598 H

C	-0.89583900	-1.61030200	-0.43037600
C	-0.92875700	-0.03020600	-0.62021300
C	-0.22155600	0.49683300	-1.95272300
C	0.60952000	1.63383700	-1.52613100
C	1.92116800	1.54779900	-1.04271100
C	2.59073900	0.32540500	-1.19256800
C	1.54607000	-1.53522000	0.47666800
C	0.13459200	-2.00688000	0.67429600
C	-2.33844400	-1.91676500	0.07739000
C	-3.24405200	-0.84514200	-0.54155800
C	-2.41038800	0.45967900	-0.53487500
C	3.86763600	-0.08703500	-0.53591700
C	3.63684200	-0.20141100	0.99283700
C	2.26907700	-0.76990600	1.34527900
C	-2.66136500	1.42247700	0.65700500
C	-4.03779900	2.09749400	0.53911600
C	-2.49180100	0.78324400	2.04451700
C	-0.63385500	-2.40180400	-1.72190900
C	1.79755100	-0.38090600	2.72110800
C	2.52304500	2.71412300	-0.28704700
H	-0.37672300	0.38805000	0.23263800
H	-1.89695900	2.21714300	0.56499300
H	-0.98661300	0.80638800	-2.67118900
H	0.36610400	-0.30209900	-2.40788900
H	0.09825000	2.59302200	-1.40242600
H	2.17704300	-0.36382500	-1.92474400
H	2.04747100	-1.91491800	-0.41537700
H	0.13378500	-3.10778200	0.73667000
H	-0.24874000	-1.63798800	1.63141500
H	-2.64800500	-2.93719300	-0.18099000
H	-2.36521400	-1.84019000	1.17138400
H	-4.19451300	-0.74235900	-0.00477700
H	-3.49295900	-1.11530700	-1.57711400
H	-2.65333400	1.04050300	-1.43580100
H	4.65970800	0.65310500	-0.72910500
H	4.20390700	-1.04113200	-0.95637100
H	3.69995600	0.80440500	1.42857300
H	4.44063400	-0.78060600	1.47291100
H	-4.15559300	2.60380400	-0.42857800
H	-4.85038500	1.36452500	0.63532900
H	-4.17438400	2.84454600	1.33137000
H	-3.25679900	0.01681000	2.22613300
H	-1.50539400	0.31673100	2.17491100
H	-2.60053100	1.54324000	2.82882900
H	-1.32358600	-2.10719500	-2.52335100
H	0.39149000	-2.28749100	-2.10034600
H	-0.78202400	-3.47250200	-1.53504600
H	2.62791600	-0.46796800	3.43708300
H	0.96562600	-0.98493000	3.09144100
H	1.49094700	0.67761800	2.73753200
H	3.61378800	2.74668000	-0.38430500
H	2.28088800	2.65223800	0.78266900
H	2.11993600	3.66279300	-0.65959300

Structure 4 = B-conformational_bending_TS
B97D3/6-31G** = 781.2730374 H, NIMAG=1, -20 cm-1
Gibbs-correction (298.15 K, entropic quasi-harmonic correction)= 0.0019640 H
mPW1PW91/6-311+G(d,p)// B97D3/6-31G** = 781.7183078 H

C	-0.83246200	-1.53978600	-0.47658600
C	-1.01040300	0.03770600	-0.55676100
C	-0.30070700	0.75018900	-1.79753600
C	0.55956100	1.81062500	-1.24186700
C	1.89155700	1.67115900	-0.84590400
C	2.55532100	0.48383700	-1.19628800
C	1.66391900	-1.48611500	0.30798100
C	0.25740900	-1.94596700	0.56025700
C	-2.21916000	-1.99130600	0.07397600
C	-3.24456900	-1.01301900	-0.50682600
C	-2.54144900	0.36782300	-0.51048300
C	3.89645200	0.02749500	-0.71465200
C	3.81937400	-0.26476000	0.80454300
C	2.47516400	-0.85282500	1.20635000
C	-2.91367100	1.31420700	0.66235600
C	-4.34415400	1.85446600	0.50158400
C	-2.72347100	0.69699000	2.05759700
C	-0.58121400	-2.21401200	-1.83498600
C	2.11638400	-0.62851700	2.65026300
C	2.53931300	2.73157400	0.01928200
H	-0.55173500	0.43554000	0.35972100
H	-2.22604900	2.17732300	0.58523900
H	-1.06850700	1.18354900	-2.44627600
H	0.25711900	0.01936200	-2.38603200
H	0.05587900	2.74021500	-0.96106800
H	2.09716000	-0.10521200	-1.98715900
H	2.09363200	-1.79194700	-0.64727300
H	0.25797500	-3.04889500	0.60260400
H	-0.07694200	-1.59664400	1.54358400
H	-2.43036800	-3.03841500	-0.17762200
H	-2.21582700	-1.91657000	1.16921500
H	-4.18140300	-1.00781100	0.06251100
H	-3.50175000	-1.29944000	-1.53540600
H	-2.82342600	0.90339900	-1.42725600
H	4.65583400	0.80215200	-0.90406800
H	4.19859600	-0.86544700	-1.27292100
H	3.94574500	0.67917100	1.35077800
H	4.64986200	-0.90812500	1.13348500
H	-4.48022500	2.34635600	-0.47111500
H	-5.08537000	1.04699200	0.57582000
H	-4.57538800	2.58564900	1.28664600
H	-3.41766200	-0.13796400	2.22133100
H	-1.70188400	0.32409900	2.21916400
H	-2.92493600	1.44479800	2.83513300
H	-1.33071400	-1.91352500	-2.57816800
H	0.41006900	-1.99164800	-2.25346100
H	-0.64008500	-3.30412700	-1.72620800
H	3.00481200	-0.77565200	3.28118900
H	1.32531300	-1.28827600	3.01556200
H	1.79843600	0.41474800	2.81055200
H	3.61550600	2.81380800	-0.17035100
H	2.40192300	2.50255800	1.08472600
H	2.08525600	3.71139800	-0.16666700

Structure 5 = B-conformer_2
B97D3/6-31G** = 781.2765819 H, NIMAG=0
Gibbs-correction (298.15 K, entropic quasi-harmonic correction)= 0.0026830 H
mPW1PW91/6-311+G(d,p)// B97D3/6-31G** = 781.7240273 H

C	-0.63637700	-1.28852000	-0.27878100
C	-1.26437500	0.14063800	-0.12357900
C	-0.64405600	1.31975300	-0.94437900
C	0.41569900	2.02431300	-0.17259200
C	1.77565300	1.79881400	-0.18807600
C	2.29270000	0.81838900	-1.09058900
C	1.98786100	-1.15107200	-0.11771000
C	0.67505000	-1.51891900	0.52118400
C	-1.76154000	-2.12751500	0.39233000
C	-3.08959200	-1.52519200	-0.10203600
C	-2.77741600	-0.03672400	-0.44197400
C	3.75932400	0.50228300	-1.32304400
C	4.31131900	-0.25612200	-0.09518100
C	3.16222700	-0.95922700	0.59040400
C	-3.69640500	1.00497500	0.24353500
C	-5.13068200	0.89738900	-0.30249800
C	-3.69241000	0.92977600	1.77975000
C	-0.48662100	-1.73010200	-1.74541000
C	3.27965400	-1.25958600	2.05158800
C	2.68424600	2.48806400	0.80396600
H	-1.17346800	0.39060000	0.94450700
H	-3.31669100	2.00416100	-0.03702700
H	-1.44793500	2.04138800	-1.14241800
H	-0.30194100	0.95349900	-1.91905500
H	0.04782200	2.74483900	0.56336600
H	1.62477400	0.56746700	-1.91251100
H	2.08360700	-1.37076800	-1.18261300
H	0.73399500	-2.60179300	0.73486300
H	0.58623700	-1.02819600	1.50098400
H	-1.65713500	-3.19762100	0.16899100
H	-1.68126400	-2.01353200	1.48420000
H	-3.87386600	-1.62288300	0.65622000
H	-3.45353200	-2.05196200	-0.99228600
H	-2.90092600	0.10777300	-1.52717700
H	4.32433600	1.42696000	-1.50756300
H	3.84519600	-0.10919000	-2.22901100
H	4.74164400	0.45324300	0.62492800
H	5.12853400	-0.95273200	-0.34055800
H	-5.15033800	0.99491200	-1.39657900
H	-5.58124500	-0.07061900	-0.04312500
H	-5.77010900	1.68311600	0.11955400
H	-4.08252300	-0.03205900	2.13912900
H	-2.69006700	1.06641500	2.20861400
H	-4.33387900	1.71604500	2.19748900
H	-1.44558300	-1.68946600	-2.27310400
H	0.21500500	-1.10932600	-2.31880500
H	-0.12678500	-2.76654800	-1.79701800
H	2.77804400	-2.19653500	2.32174800
H	2.79591700	-0.46181600	2.64053900
H	4.32887400	-1.29858400	2.36618500
H	3.59985900	2.86052900	0.32670900
H	2.98985800	1.79797100	1.60265100
H	2.17803700	3.33620800	1.27671100

Structure 6 = TS-B-to-C
B97D3/6-31G** = 781.2760808 H, NIMAG=1, -330 cm-1
Gibbs-correction (298.15 K, entropic quasi-harmonic correction)= 0.0022040 H
mPW1PW91/6-311+G(d,p)// B97D3/6-31G** = 781.7271550 H

C	-0.60808600	-1.23447000	-0.25155300
C	-1.29266600	0.16613700	-0.09949500
C	-0.69546000	1.37122100	-0.88742100
C	0.38189400	2.04465100	-0.10268100
C	1.72762700	1.78123300	-0.11897600
C	2.23483400	0.78799200	-1.04061300
C	2.01646300	-1.02499900	-0.15563600
C	0.72379900	-1.39777600	0.53189800
C	-1.69055500	-2.11452600	0.43526900
C	-3.04720000	-1.56577400	-0.05004900
C	-2.78992700	-0.07738000	-0.43865700
C	3.71611600	0.57555700	-1.35565400
C	4.34839300	-0.20985100	-0.18573100
C	3.24144300	-0.94094600	0.52346200
C	-3.76823100	0.94605000	0.18973800
C	-5.19158100	0.72861100	-0.35289200
C	-3.76695000	0.95356200	1.72770600
C	-0.45574300	-1.68453100	-1.71610200
C	3.41728600	-1.36948500	1.93914200
C	2.65308300	2.41100400	0.89322200
H	-1.23099600	0.40725000	0.97319300
H	-3.44543600	1.94854200	-0.14321900
H	-1.50111600	2.09843700	-1.05308700
H	-0.36536000	1.04057900	-1.87974800
H	0.03104100	2.76671100	0.63972300
H	1.56813700	0.60433300	-1.88209200
H	2.10546200	-1.35415400	-1.19653700
H	0.81887700	-2.46595300	0.79557900
H	0.64591800	-0.86048800	1.48830800
H	-1.54690000	-3.18087600	0.21506600
H	-1.60385800	-1.99293500	1.52582100
H	-3.81088100	-1.66347600	0.72902700
H	-3.41538400	-2.13002000	-0.91516900
H	-2.89774600	0.02372700	-1.53063900
H	4.22404500	1.53421800	-1.52184000
H	3.79472400	0.00661100	-2.29003400
H	4.81010300	0.47466900	0.54064200
H	5.15827700	-0.89541500	-0.48499900
H	-5.20947600	0.75799900	-1.45098600
H	-5.59235300	-0.24411700	-0.03596700
H	-5.87406900	1.50386700	0.01804500
H	-4.08211500	-0.01523200	2.13876600
H	-2.78052200	1.19227400	2.14843100
H	-4.47012000	1.70850800	2.10173000
H	-1.42395200	-1.70829400	-2.22722300
H	0.19580000	-1.02916100	-2.30969300
H	-0.03832200	-2.69983100	-1.76245600
H	3.02467300	-2.38327400	2.10019100
H	2.82694400	-0.71423500	2.60188100
H	4.46280000	-1.31650500	2.25916000
H	3.56360000	2.81021600	0.42646400
H	2.97304700	1.67092300	1.64266200
H	2.15793300	3.22743100	1.42913900

Structure 7 = C
B97D3/6-31G** = 781.2857408 H, NIMAG=0
Gibbs-correction (298.15 K, entropic quasi-harmonic correction)= 0.0021080 H
mPW1PW91/6-311+G(d,p)// B97D3/6-31G** = 781.7444617 H

C	-0.54305100	-1.13415700	-0.19981700
C	-1.33236800	0.20935900	-0.07140200
C	-0.78978700	1.45701600	-0.81776100
C	0.29285300	2.09619200	0.00099100
C	1.60673700	1.75675900	0.02028700
C	2.12581300	0.71933000	-0.92654200
C	2.06043500	-0.71850400	-0.22449800
C	0.81473500	-1.17285900	0.55412100
C	-1.54494200	-2.07829100	0.52343500
C	-2.94871000	-1.63481800	0.05292700
C	-2.79376400	-0.16094100	-0.43679800
C	3.61862200	0.79484000	-1.33970300
C	4.37748400	-0.00656900	-0.26164100
C	3.37044200	-0.87068500	0.39804100
C	-3.87186700	0.82145700	0.08322900
C	-5.26316300	0.40876100	-0.42928400
C	-3.88063100	0.99308100	1.61134700
C	-0.37636900	-1.61599300	-1.65384400
C	3.67170000	-1.73878500	1.54947600
C	2.52029100	2.24244400	1.10986800
H	-1.31766200	0.44544900	1.00493100
H	-3.64781900	1.80876000	-0.35633600
H	-1.61097400	2.17344400	-0.94544600
H	-0.46027100	1.18017000	-1.82784900
H	-0.04933400	2.81689900	0.74851100
H	1.48978100	0.68587500	-1.81270000
H	2.17627000	-1.37617800	-1.11907500
H	0.99459700	-2.21578300	0.85660200
H	0.73704300	-0.58719500	1.48115800
H	-1.32992700	-3.13542100	0.31614800
H	-1.44723700	-1.93361800	1.61004400
H	-3.67516700	-1.72000500	0.86851500
H	-3.31803500	-2.27484100	-0.75711400
H	-2.87135500	-0.14535600	-1.53595900
H	3.98172600	1.82282200	-1.43291400
H	3.74466400	0.31322900	-2.31778600
H	4.81934800	0.63815900	0.51943900
H	5.23236100	-0.60595500	-0.62015000
H	-5.27169800	0.30597700	-1.52316700
H	-5.57694900	-0.55215800	0.00127800
H	-6.01791400	1.15637600	-0.15371100
H	-4.06834100	0.04107100	2.12709200
H	-2.93530500	1.40234000	1.99293400
H	-4.67791300	1.68595300	1.90919000
H	-1.34657700	-1.74979700	-2.14342700
H	0.19788600	-0.92065000	-2.28038800
H	0.12919500	-2.59236100	-1.67730700
H	3.36560600	-2.77518600	1.32958000
H	3.04115300	-1.44275100	2.40438200
H	4.72489200	-1.71564800	1.84278900
H	3.46007000	2.66564400	0.72534200
H	2.80575700	1.40007200	1.77022900
H	2.03536500	2.99483000	1.74054500

Structure 8 = TS-C-to-D
B97D3/6-31G** = 781.2750780 H, NIMAG=1, -740 cm-1
Gibbs-correction (298.15 K, entropic quasi-harmonic correction)= 0.0020790 H
mPW1PW91/6-311+G(d,p)// B97D3/6-31G** = 781.7421371 H

C	0.53182600	-1.11220400	0.15294400
C	1.34864100	0.21727500	0.03609700
C	0.81489500	1.48310300	0.74618000
C	-0.26569700	2.16285400	-0.05630600
C	-1.57717800	1.84705300	-0.08284700
C	-2.08381600	0.73115100	0.82610800
C	-2.03880900	-0.54780200	-0.01074800
C	-0.79747500	-1.12537800	-0.64715900
C	1.52831400	-2.08457200	-0.53579700
C	2.92571000	-1.66675700	-0.02385200
C	2.78732400	-0.18643900	0.45092200
C	-3.55317200	0.80759200	1.31704300
C	-4.37282600	-0.02417400	0.30723100
C	-3.36945000	-0.95366400	-0.33296000
C	3.90600400	0.76639100	-0.03928600
C	5.27013500	0.32046100	0.51615700
C	3.96403600	0.93179200	-1.56694300
C	0.29947700	-1.59235800	1.59939800
C	-3.77555800	-2.03155300	-1.28424300
C	-2.54623700	2.48723500	-1.04599100
H	1.37164300	0.44151700	-1.04261500
H	3.69408200	1.76043300	0.39032600
H	1.64764700	2.18649000	0.87002000
H	0.48293000	1.23250600	1.76474100
H	0.07297700	2.94841700	-0.73651100
H	-1.40035900	0.64924400	1.67273900
H	-2.62603000	-1.44394600	0.71328000
H	-1.01454000	-2.16161300	-0.94231600
H	-0.66464100	-0.56884600	-1.58827800
H	1.28558000	-3.13592500	-0.32888500
H	1.46849100	-1.94516600	-1.62593100
H	3.67400700	-1.77331400	-0.81675100
H	3.25555000	-2.30993100	0.80053300
H	2.83034500	-0.16606400	1.55205100
H	-3.91305900	1.83551400	1.41036900
H	-3.62263400	0.34571800	2.31088800
H	-4.76791900	0.59453700	-0.51427000
H	-5.22806100	-0.55703900	0.73936500
H	5.24282500	0.22034300	1.61001200
H	5.57359400	-0.64888000	0.09714200
H	6.05120000	1.04854800	0.26257100
H	4.13882600	-0.02720200	-2.07443000
H	3.04276200	1.36749000	-1.97680000
H	4.78963000	1.59981600	-1.84358800
H	1.24909600	-1.77343300	2.11293000
H	-0.26210600	-0.88040000	2.21599500
H	-0.24928400	-2.54595200	1.59898300
H	-2.97357300	-2.74007400	-1.50968600
H	-4.05793600	-1.53561600	-2.22618200
H	-4.66007300	-2.56779200	-0.92292800
H	-3.40907900	2.93807500	-0.53537900
H	-2.94533300	1.75628800	-1.76875800
H	-2.05343600	3.27621100	-1.62433600

Structure 9 = D
B97D3/6-31G** = 781.2917608 H, NIMAG=0
Gibbs-correction (298.15 K, entropic quasi-harmonic correction)= 0.0021300 H
mPW1PW91/6-311+G(d,p)// B97D3/6-31G** = 781.7522081 H

C	-0.60528400	-1.15988100	-0.25522900
C	-1.34085300	0.19952900	-0.07132600
C	-0.75525700	1.42359300	-0.81133700
C	0.39490300	2.02923200	-0.06680400
C	1.70068800	1.64592500	-0.05046400
C	2.14002900	0.49955600	-1.02879700
C	1.97741100	-0.57362100	-0.03427100
C	0.75206000	-1.24366400	0.47284600
C	-1.61685900	-2.10003400	0.45502000
C	-3.01142600	-1.59491700	0.01344300
C	-2.81916200	-0.10878100	-0.42397100
C	3.62445300	0.49787300	-1.44267400
C	4.37206100	-0.11222400	-0.23703700
C	3.32313100	-1.02792800	0.43755100
C	-3.85280700	0.88987900	0.15222600
C	-5.26321700	0.55473400	-0.36411300
C	-3.84180200	0.99082900	1.68666900
C	-0.46325800	-1.61356700	-1.72287200
C	3.51779500	-1.34229700	1.92863900
C	2.71555900	2.36266200	0.80847600
H	-1.29992700	0.41698300	1.00796000
H	-3.59331800	1.88699700	-0.24451600
H	-1.53783500	2.18482300	-0.91275300
H	-0.46722400	1.13719200	-1.83444100
H	0.15319200	2.90348400	0.54384700
H	1.43282500	0.48271100	-1.85944200
H	1.03315900	-2.30748500	0.57813300
H	0.63811800	-0.91989100	1.52434300
H	-1.44096400	-3.15627000	0.21165400
H	-1.50529700	-1.99387000	1.54486900
H	-3.73154200	-1.68892300	0.83334500
H	-3.40541800	-2.19398900	-0.81560200
H	-2.91342400	-0.05059800	-1.52022800
H	3.97971000	1.49490600	-1.72245600
H	3.72725000	-0.14598400	-2.32623900
H	4.70487100	0.65602300	0.46807800
H	5.25979900	-0.67902000	-0.53629200
H	-5.28640200	0.50636100	-1.46145500
H	-5.60828100	-0.41400000	0.02278400
H	-5.98663500	1.31504000	-0.04354200
H	-4.07265400	0.02652800	2.16001400
H	-2.87596300	1.33677800	2.07972900
H	-4.60254700	1.70665500	2.02276700
H	-1.44377200	-1.71851800	-2.19817700
H	0.12176000	-0.92794600	-2.34576700
H	0.02539400	-2.59652100	-1.76575100
H	2.80054500	-2.08592100	2.29369500
H	3.41007800	-0.43071000	2.52896700
H	4.52648500	-1.73775500	2.08945200
H	3.57884800	2.70351900	0.22418600
H	3.09331400	1.74196800	1.63021000
H	2.24976800	3.24637900	1.25857500
H	3.34710100	-2.00033500	-0.11179700
